# On the Effect of Fast Rotation and Vertical Viscosity on the Lifespan of the 3*D* Primitive Equations

**DOI:** 10.1007/s00021-022-00705-3

**Published:** 2022-06-15

**Authors:** Quyuan Lin, Xin Liu, Edriss S. Titi

**Affiliations:** 1 Department of Mathematics, University of California, Santa Barbara, CA 93106 USA; 2 Weierstrass-Institut für Angewandte Analysis und Stochastik, Leibniz-Institut im Forschungsverbund Berlin, Mohrenstr. 39, 10117 Berlin, Germany; 3 Department of Applied Mathematics and Theoretical Physics, University of Cambridge, Cambridge, CB3 0WA UK; 4 Department of Mathematics, Texas A &M University, College Station, Texas TX 77840 USA; 5 Department of Computer Science and Applied Mathematics, Weizmann Institute of Science, Rehovot, 76100 Israel

**Keywords:** Anisotropic vertically viscous primitive equations, Fast rotation, Well-posedness theory, Hydrostatic Navier-Stokes equations, 35Q35, 35Q86, 86A10, 76E07

## Abstract

We study the effect of the fast rotation and vertical viscosity on the lifespan of solutions to the three-dimensional primitive equations (also known as the hydrostatic Navier-Stokes equations) with impermeable and stress-free boundary conditions. Firstly, for a short time interval, independent of the rate of rotation $|\Omega |$, we establish the local well-posedness of solutions with initial data that is analytic in the horizontal variables and only $L^2$ in the vertical variable. Moreover, it is shown that the solutions immediately become analytic in all the variables with increasing-in-time (at least linearly) radius of analyticity in the vertical variable for as long as the solutions exist. On the other hand, the radius of analyticity in the horizontal variables might decrease with time, but as long as it remains positive the solution exists. Secondly, with fast rotation, i.e., large $|\Omega |$, we show that the existence time of the solution can be prolonged, with “well-prepared” initial data. Finally, in the case of two spatial dimensions with $\Omega =0$, we establish the global well-posedness provided that the initial data is small enough. The smallness condition on the initial data depends on the vertical viscosity and the initial radius of analyticity in the horizontal variables.

## Introduction

We consider the following 3*D* viscous primitive equations (PEs) with only vertical viscosity for the large-scale oceanic and atmospheric dynamics:

1.1a $$\begin{aligned}&\partial _t \mathcal {V} + \mathcal {V}\cdot \nabla \mathcal {V} + w\partial _z \mathcal {V} - \nu \partial _{zz} \mathcal {V} +\Omega \mathcal {V}^\perp + \nabla p = 0 , \end{aligned}$$

1.1b $$\begin{aligned}&\partial _z p = 0, \end{aligned}$$

1.1c $$\begin{aligned}&\nabla \cdot \mathcal {V} + \partial _z w =0, \end{aligned}$$

in the horizontal channel $\mathcal {D}:=\big \{(\varvec{x},z)^\top = (x,y,z)^\top : \varvec{x}^\top \in \mathbb {T}^2,z \in (0,1) \big \}$, subject to the following initial and boundary conditions:

1.2 $$\begin{aligned} \mathcal {V}|_{t=0} =&\mathcal {V}_0, \end{aligned}$$

1.3 $$\begin{aligned} (\partial _z \mathcal {V}, w)|_{z=0,1}=&0, \;\text {and} \; (\mathcal {V}, w)\;\text {are periodic in} \; \varvec{x} \; \text {with period} \; 1. \end{aligned}$$

Here the horizontal velocity field $\mathcal {V}=(u,v)^\top$, the vertical velocity *w*, and the pressure *p* are the unknowns of the initial-boundary value problem. The 2*D* horizontal gradient is denoted by $\nabla = (\partial _x, \partial _y)^\top$. The positive constant $\nu$ is the vertical viscosity coefficient. $\Omega \mathcal {V}^\perp = \Omega (-v, u)^\top$ represents the Coriolis force with magnitude $|\Omega | \in {\mathbb {R}}^+$. As one will see later, the Coriolis force induces linear rotation waves with rotating rate $|\Omega |$. The 3*D* viscous PEs can be derived as the asymptotic limit of the small aspect ratio between the vertical and horizontal length scales from the Boussinesq system, which is justified rigorously first in [1] in a weak sense, then later in [41] in a strong sense with error estimates (see also a recent paper [42] for the PEs with anisotropic horizontal viscosity). Notice that we have omitted the coupling with temperature in (1.1) for the sake of simple and clear presentation. System (1.1) is also referred to as the anisotropic vertically viscous hydrostatic Navier-Stokes equations.

The global well-posedness of strong solutions to the 3*D* PEs with full viscosity was first established in [15], and later in [32]. See also [37, 38] for different boundary conditions, and [27] for solutions with less regular initial data. In [11–13], the authors consider global well-posedness of strong solutions to the 3*D* PEs with only horizontal viscosity.

In the inviscid case without rotation ($\Omega = 0$), the linear ill-posedness of solutions in Sobolev spaces has been established in [47]. Later on, the nonlinear ill-posedness of the inviscid PEs without rotation was established in [26]. Moreover, without rotation, it was proved that smooth solutions to the inviscid PEs can develop singularity in finite time [10, 48]. It is shown later in [29] that these results can be extended to the case with rotation, i.e., $\Omega \ne 0$. Recently, the stability of the blowup is studied in [17]. Under some structural (local Rayleigh condition) or analyticity assumption of the initial data, the well-posedness theory was studied in [8, 9, 23, 24, 35, 36, 44]. In particular, it has been shown that the lifespan of solutions to the 3*D* inviscid PEs can be prolonged provided that the rate of rotation is fast enough and the initial data is “well-prepared” in [23]. Similar results have been studied in the case of the 3*D* fast rotating Euler, Navier-Stokes, and Boussinesq equations in [3–6, 16, 18, 19, 30, 33] (see also [2, 25, 34, 43] for some explicit examples demonstrating the mechanism).

For the PEs with only vertical viscosity, it has been shown in [47] that system (1.1) is ill-posed in any Sobolev space. This ill-posedness can be overcome by considering additional linear (Rayleigh-like friction) damping, see [14] for the reduced 3*D* case. On the other hand, with Gevrey regularity and some convex conditions on the initial data, the local well-posedness is established in [22]. When the initial data is analytic in the horizontal variables $\varvec{x}$ and is sufficiently small, the global well-posedness is proved in [46] in 2*D*, with $\Omega = 0$ and Dirichlet boundary condition. In this paper, we consider (1.1) in 3*D*, with arbitrary $\Omega \in \mathbb {R}$ and subject to impermeable and stress-free boundary conditions.

The main results of this paper are roughly summarized as follows:

1. Local well-posedness (see Theorem 3.1): Assume that $\mathcal {V}_0$ is analytic in the horizontal variables $\varvec{x}$ and only $L^2$ in the vertical variable *z*. Let $\Omega \in \mathbb {R}$ be arbitrary but fixed. Then there exists a positive time ${\mathcal {T}} >0$, independent of $\Omega$, such that there exists a unique Leray-Hopf type weak solution ${\mathcal {V}}$ to system (1.1) (see Definition 3.1, below). Moreover the weak solution ${\mathcal {V}}$ depends continuously on the initial data and in particular it is unique.
2. Instantaneous analyticity in the vertical variable (see Theorem 3.2): With the same assumptions as in R1 above, the unique Leray-Hopf type weak solution ${\mathcal {V}}$ immediately becomes analytic in *z* for $t>0$. Moreover, thanks to the viscous effect the radius of analyticity in *z* increases in time, at least linearly, for as long as the solution exists. On the other hand, the radius of analyticity in the horizontal variables might decrease with time, but as long as it remains positive the solution exists.
3. Long-time existence (see Theorem 5.1): Let $|\Omega |\ge |\Omega _0|$ with $|\Omega _0|$ large enough, in particular $|\Omega _0|>1$. Assume that the analytic-Sobolev norm (see (2.3), below) of both the barotropic mode $\overline{\mathcal {V}}_0$ and baroclinic mode $\widetilde{\mathcal {V}}_0$ are ${\mathcal {O}}(1)$, and that some Sobolev norm of $\widetilde{\mathcal {V}}_0$ is ${\mathcal {O}} (\frac{1}{|\Omega _0|})$, as $|\Omega _0| \rightarrow \infty$. Then a lower bound, ${\mathcal {T}}$, of the existence time of the Leray-Hopf type weak solution to system (1.1) with $|\Omega |\ge |\Omega _0|$ satisfies
  1.4 $$\begin{aligned} {\mathcal {T}} = {\mathcal {O}}( \log [\log [\log (\log (|\Omega _0|) )]] ) \rightarrow \infty \text { as } |\Omega _0|\rightarrow \infty . \end{aligned}$$
  Moreover, as a corollary of R2, the solution is analytic in all variables (see Remark 11, below).
4. Long-time existence with small barotropic mode (see Theorem 5.2): Let $|\Omega |\ge |\Omega _0| >1$ and $|\Omega _0|$ be large enough.
  - Under the assumption that the solution $\overline{V}$ to the 2*D* Euler equations with initial data $\overline{\mathcal {V}}_0$ is uniformly-in-time bounded in the analytic space norm, (1.4) can be improved to ${\mathcal {T}} = {\mathcal {O}}( \log (\log (|\Omega _0|) ) )$. Let us note that this result is parallel to a similar one in the inviscid case [23].
  - Moreover, under the assumption that $\overline{V}$ is uniformly-in-time small enough (the smallness condition is independent of ${|\Omega _0|}$) in the analytic space norm, the smallness requirement on the Sobolev norm of $\widetilde{V}_0$ can be relaxed and is independent of $\Omega _0$, and (1.4) can be improved to ${\mathcal {T}} = {\mathcal {O}}( \log (|\Omega _0| ) )$, as $|\Omega _0| \rightarrow \infty$. In view of work reported in [31] about the growth of solutions of 2D Euler equations, we observe that the above assumptions about the smallness of $\overline{V}$ might not be valid for all initial data.
  - If the analytic norm of $\overline{\mathcal {V}}_0$ is of order ${\mathcal {O}} ( \frac{1}{|\Omega _0|} )$, as $|\Omega _0| \rightarrow \infty$, then the smallness requirement on the Sobolev norm of $\widetilde{V}_0$ can be relaxed and independent of $\Omega _0$; moreover, (1.4) can be improved to ${\mathcal {T}} = {\mathcal {O}}( |\Omega _0|^{\frac{1}{2}} )$.
5. Global well-posedness in 2*D* with $\Omega =0$ (see Theorem 6.1): In the 2*D* case with $\Omega =0$, suppose that the initial data $\mathcal {V}_0$ is analytic only in the horizontal variable with small analytic-Sobolev norm (the smallness condition depends on $\nu$ and the initial radius of analyticity $\tau _0$). Then the unique Leray-Hopf type weak solution exists globally in time. Furthermore, R2 implies that the solution is analytic in all variables.

Compared to the inviscid case [23], this paper investigates the combined effect of the fast rotation and the vertical viscosity. The main differences are the following:

- With analytic initial data in all variables, aside from the fast rotation, we do not observe the effect of the vertical viscosity in prolonging the lifespan in comparison to the inviscid case in [23].
- However, with a larger class of the initial data, namely with initial data analytic in the horizontal *xy*-variables and only Sobolev in the *z*-variable, the vertical viscosity allows us to establish the local well-posedness, which is not possible for the inviscid case (see [47]). Moreover, the existence time is proportional to $\nu$ and shrinks to zero as $\nu \rightarrow 0$ (see (3.6)), which is consistent with the ill-posedness result in the inviscid case.
- Such a regularizing effect of the vertical viscosity can also be seen in the proof of Theorem 5.1 in (5.32) and the absorbing argument (5.36).

Compared to the work [46], which studies the 2*D* model subject to Dirichlet boundary condition without rotation, we investigate here both the 2*D* and 3*D* models subject to the impermeable and stress-free boundary conditions. While recognizing the subtle difference between the imposed boundary conditions and their mathematical and physical implications, the result reported in [46] is, roughly speaking, along the lines of the statement in R5, above, focusing on the 2*D* case. Meanwhile, our main objective in this contribution is to study the combined effect of the fast rotation and viscosity in the 3*D* case, as it has been summarized in R1 – R4 above.

The paper is organized as follows. In section 2, we introduce the notations and some preliminary results which will be used throughout this paper. In section 3, we establish the local well-posedness of system (1.1) and instantaneous analytic regularity in the vertical variable by proving Theorem 3.1 (i.e., R1) and Theorem 3.2 (i.e., R2). In section 4, we derive the formal limit resonant system of (1.1) when $|\Omega |\rightarrow \infty$ and establish some properties about the limit system. Section 5 is the centerpiece of this paper and is devoted to studying the effect of rotation, where we prove Theorem 5.1 (i.e., R3) and Theorem 5.2 (i.e., R4). In section 6, we prove the global well-posedness in the 2*D* case with $\Omega =0$, i.e., Theorem 6.1 (i.e., R5).

## Preliminaries

In this section, we introduce the notations and collect some preliminary results that will be used in this paper. The generic constant *C* appearing in this paper may change from line to line. We use subscript, e.g., $C_r$, to emphasize the dependence of the constant on *r*.

### Functional Settings

We use the notation $(\varvec{x},z) = (x,y,z) \in \mathcal {D}=\mathbb {T}^2\times [0,1]$, where $\varvec{x}$ and *z* represent the horizontal and vertical variables, respectively. $\mathbb {T}^2$ is the two-dimensional torus with unit length. Denote by $L^2(\mathcal {D})$, the Lebesgue space of complex/real valued functions $f(\varvec{x},z)$ satisfying $\int _{\mathcal {D}} |f(\varvec{x},z)|^2 d\varvec{x} dz < \infty$, endowed with the norm

$$\begin{aligned} \Vert f \Vert :=\Vert f\Vert _{L^2(\mathcal {D})} = (\int _{\mathcal {D}} |f(\varvec{x},z)|^2 d\varvec{x} dz)^{\frac{1}{2}}, \end{aligned}$$

and the inner product

2.1 $$\begin{aligned} \langle f,g\rangle := \int _{\mathcal {D}} f(\varvec{x},z)g^*(\varvec{x},z) \;d\varvec{x} dz \end{aligned}$$

for $f,g \in L^2(\mathcal {D})$. Here $g^*$ represents the complex conjugate of *g*. Given any time $\mathcal {T}>0$, $L^p(0,\mathcal {T};X)$ represents the space of functions $f: [0,T]\rightarrow X$ satisfying $\int _0^\mathcal {T} \Vert f(t)\Vert _X^p dt < \infty$, where *X* is a Banach space with norm $\Vert \cdot \Vert _X$. For a function $f \in L^2(\mathcal {D})$, we use $\hat{f}_{\varvec{k}}(z), \varvec{k} \in 2\pi \mathbb {Z}^2$, to denote its Fourier coefficients in the $\varvec{x}$-variables, i.e.,

2.2 $$\begin{aligned}&\hat{f}_{\varvec{k}}(z) := \int _{\mathbb {T}^2} e^{- i\varvec{k}\cdot \varvec{x}} f(\varvec{x},z) d\varvec{x}, \qquad \text {and hence} \qquad f(\varvec{x},z) = \sum \limits _{\varvec{k}\in 2\pi \mathbb {Z}^2} \hat{f}_{\varvec{k}}(z) e^{ i\varvec{k}\cdot \varvec{x}}. \end{aligned}$$

Let $A := \sqrt{-\Delta _h}$, where $\Delta _h = \partial _{xx} + \partial _{yy}$ is the horizontal Laplacian, defined by, in terms of the Fourier coefficients,

$$\begin{aligned} \widehat{Af}_{\varvec{k}} (z) := |\varvec{k}| \hat{f}_{\varvec{k}}(z), \qquad {\varvec{k}} \in 2\pi \mathbb {Z}^2. \end{aligned}$$

For $r \ge 0$, we define

$$\begin{aligned} H^r(\mathcal {D}) : = \{ f\in L^2(\mathcal {D}): \Vert f\Vert _{H^r} <\infty \}, \end{aligned}$$

with

$$\begin{aligned} \Vert f\Vert _{H^r} : = \sum \limits _{0 \le m\le r, m \in \mathbb {Z}} \big (\Vert A^{r-m} \partial _z^m f\Vert ^2 + \Vert \partial _z^m f\Vert ^2\big )^{\frac{1}{2}}. \end{aligned}$$

Notice that, with (2.2), we have

$$\begin{aligned} \Vert \partial _z^m f\Vert ^2 =\int _0^1 \Big ( \sum \limits _{\varvec{k}\in 2\pi \mathbb {Z}^2} |\partial ^m_{z}\hat{f}_{\varvec{k}}(z)|^2 \Big ) dz \quad \text {and} \quad \Vert A^{r-m} \partial _z^m f\Vert ^2 =\int _0^1 \Big ( \sum \limits _{\varvec{k}\in 2\pi \mathbb {Z}^2} |\varvec{k}|^{2(r-m)} |\partial ^m_{z}\hat{f}_{\varvec{k}}(z)|^2 \Big ) dz. \end{aligned}$$

In addition, given any $r\ge 0$ and $s \ge 0$ with $s \in {\mathbb {Z}}$, we define the anisotropic Sobolev space

$$\begin{aligned} H^r_{\varvec{x}}H^s_z(\mathcal {D}) : = \{ f\in L^2(\mathcal {D}): \Vert f\Vert _{H^r_{\varvec{x}}H^s_z} <\infty \}, \end{aligned}$$

where the anisotropic Sobolev norm is given by

$$\begin{aligned} \Vert f\Vert _{H^r_{\varvec{x}}H^s_z} := \sum \limits _{m\le s}\big (\Vert A^r \partial _z^m f\Vert ^2 + \Vert \partial _z^mf\Vert ^2\big )^{\frac{1}{2}}. \end{aligned}$$

On the other hand, given any $r \ge 0$, $s\ge 0$, and $\tau \ge 0$, with $s \in {\mathbb {Z}}$, we define the analytic-Sobolev space

$$\begin{aligned} \mathcal {S}_{r,s,\tau } := \{ f\in L^2(\mathcal {D}): \Vert f\Vert _{r,s,\tau } <\infty \}, \end{aligned}$$

where the norm is given by

2.3 $$\begin{aligned} \Vert f\Vert _{r,s,\tau } := \sum \limits _{m\le s}(\Vert A^r e^{\tau A} \partial _z^m f\Vert ^2 + \Vert \partial _z^mf\Vert ^2)^{\frac{1}{2}}, \end{aligned}$$

with, recalling (2.2),

$$\begin{aligned} \Vert A^r e^{\tau A} \partial _z^m f\Vert ^2 :=\int _0^1 \Big ( \sum \limits _{\varvec{k}\in 2\pi \mathbb {Z}^2} |\varvec{k}|^{2r} e^{2\tau |\varvec{k}|} |\partial ^m_{z}\hat{f}_{\varvec{k}}(z)|^2 \Big ) dz. \end{aligned}$$

Roughly speaking, $\mathcal {S}_{r,s,\tau }$ is the space of functions that are analytic with radius $\tau$ in the $\varvec{x}$-variables, and $H^s$ in the *z*-variable. The space of analytic functions is a special case of Gevrey class. For more details about Gevrey class, we refer readers to [20, 21, 23, 40]. Notice that when $\tau = 0$, one has $\mathcal {S}_{r,s,0} = H^r_{\varvec{x}} H^s_z (\mathcal {D})$.

#### Remark 1

With abuse of notation, we also write $f\in \mathcal {S}_{r,0,\tau }$ for $f = f(\varvec{x})$ depending only on the horizontal variables.

The following lemma summarizes the algebraic property of functions with analyticity in the horizontal variables:

#### Lemma 2.1

For $\tau \ge 0$ and $r>1$, we have

$$\begin{aligned} \Vert A^r e^{\tau A} (fg)(z)\Vert _{L^2_{\varvec{x}}} \le C_{r} \Big (|\hat{f}_0(z)|+\Vert A^r e^{\tau A} f(z)\Vert _{L^2_{\varvec{x}}} \Big ) \Big (|\hat{g}_0(z)|+ \Vert A^r e^{\tau A} g(z)\Vert _{L^2_{\varvec{x}}} \Big ), \end{aligned}$$

provided that the right hand side is bounded, where, according to (2.2),

$$\begin{aligned} \hat{f}_0(z) = \int _{\mathbb {T}^2} f(\varvec{x},z) d \varvec{x}. \end{aligned}$$

The proof of Lemma 2.1 is standard. We refer to [20, 23, 45] for details.

With $\varvec{k} = (k_1,k_2,k_3) \in 2\pi \bigl ({\mathbb {Z}}^2 \times ( {\mathbb {Z}}_+ \cup \lbrace 0 \rbrace ) \bigr )$ , we define

2.4 $$\begin{aligned} \phi _{\varvec{k}} = \phi _{k_1,k_2,k_3} := {\left\{ \begin{array}{ll} \sqrt{2}e^{ i\left( k_1 x_1 + k_2 x_2 \right) }\cos (\frac{1}{2} k_3 z) &{} \text {if} \; k_3\ne 0,\\ e^{i\left( k_1 x_1 + k_2 x_2 \right) } &{} \text {if} \; k_3=0, \end{array}\right. } \end{aligned}$$

and

2.5 $$\begin{aligned}&\mathscr {V}:= \{ \phi \in C^\infty (\mathcal {D}) \; \Big | \; \phi = \sum \limits _{\varvec{k}\in 2\pi \bigl ({\mathbb {Z}}^2 \times ({\mathbb {Z}}_+ \cup \lbrace 0 \rbrace ) \bigr )} a_{\varvec{k}} \phi _{\varvec{k}}, \; a_{-k_1, -k_2,k_3}=a_{k_1,k_2,k_3}^{*}, \; \int _0^1 \nabla \cdot \phi =0 \}. \end{aligned}$$

Here $a^*$ denotes the complex conjugate of *a*. Let

$H :=$ the closure of $\mathscr {V}$ in $L^2(\mathcal {D})$ and $V:=$ the closure of $\mathscr {V}$ in $H^1(\mathcal {D})$,

with norms given by

$$\begin{aligned} \Vert \cdot \Vert _H := \Vert \cdot \Vert _{L^2(\mathcal {D})} \text { and } \Vert \cdot \Vert _V := \Vert \cdot \Vert _{H^1(\mathcal {D})}, \hbox { respectively}. \end{aligned}$$

Then one has

$$\begin{aligned} V \subset H \equiv H' \subset V', \quad V \hookrightarrow \hookrightarrow H \hookrightarrow \hookrightarrow V'. \end{aligned}$$

### Projections and Reformulation of the Problem

In this paper, we assume that $\int _{\mathcal {D}} \mathcal {V}_0(\varvec{x},z) d\varvec{x} dz = 0$. This assumption is made to simplify the mathematical presentation. In fact, integrating (1.1a) in $\mathcal {D}$ leads to, after applying integration by parts, (1.1c), and (1.3),

2.6 $$\begin{aligned} \partial _t \int _{\mathcal {D}} \mathcal {V} d\varvec{x}dz + \Omega \int _{\mathcal {D}} \mathcal {V}^\perp d\varvec{x}dz =0. \end{aligned}$$

Therefore, under our assumption, one has

2.7 $$\begin{aligned} \int _{\mathcal {D}} \mathcal {V}(t) d\varvec{x}dz = \int _{\mathcal {D}} \mathcal {V}_0(\varvec{x},z) d\varvec{x} dz = 0. \end{aligned}$$

With slight modifications, our result applies to the case when $\int _{\mathcal {D}} \mathcal {V}_0(\varvec{x},z) d\varvec{x} dz \ne 0$.

Let

$$\begin{aligned} \dot{L}^2 : = \Big \{\varphi \in L^2(\mathcal {D},\mathbb {R}^2) : \int _{\mathcal {D}} \varphi (\varvec{x},z)d\varvec{x}dz = 0 \Big \}. \end{aligned}$$

Denote the barotropic mode and the baroclinic mode of $\mathcal {V}$ by

2.8 $$\begin{aligned} \overline{\mathcal {V}}(\varvec{x}):= \int _0^1 \mathcal {V}(\varvec{x},z)dz \quad \text {and} \quad \widetilde{\mathcal {V}}(\varvec{x},z):= \mathcal {V}-\overline{\mathcal {V}}, \qquad \text {respectively}. \end{aligned}$$

From (1.3) and (1.1c), we have

2.9 $$\begin{aligned} \nabla \cdot \overline{\mathcal {V}} = \int _0^1 \nabla \cdot \mathcal {V}(\varvec{x},z)dz = -\int _0^1 \partial _z w(\varvec{x},z)dz =0, \end{aligned}$$

and

2.10 $$\begin{aligned} w(\varvec{x},z) = -\int _0^z \nabla \cdot \widetilde{\mathcal {V}}(\varvec{x},s)ds. \end{aligned}$$

#### Remark 2

In the remaining of this paper, we will substitute *w* by its representation (2.10) without explicitly pointing it out.

Since $\nabla \cdot \overline{\mathcal {V}} =0$, and $\overline{\mathcal {V}}$ has zero mean over $\mathbb {T}^2$ thanks to (2.7), there exists a stream function $\psi (\varvec{x})$ such that $\overline{\mathcal {V}} = \nabla ^{\perp }\psi = (-\partial _{y} \psi , \partial _{x}\psi )^\top .$ Therefore, the space of solutions to (1.1) is given by

2.11 $$\begin{aligned} \begin{aligned} \mathcal {S}:= \dot{L}^2 \cap H = \Big \{\varphi \in \dot{L}^2: \nabla \cdot \overline{\varphi } = 0 \Big \} = \Big \{\varphi \in \dot{L}^2: \varphi = \nabla ^\perp \psi (\varvec{x}) + \widetilde{\varphi }(\varvec{x},z), \\ \text {for some} ~ \psi , ~ \int _{{\mathbb {T}}^2 }\psi (\varvec{x}) \,d \varvec{x} = 0 \Big \}. \end{aligned} \end{aligned}$$

Indeed, ${\mathcal {S}}$ is the analogy of “incompressible function space” for the PEs. Here ${\overline{\varphi }}$ and ${\widetilde{\varphi }}$ are the barotropic and baroclinic modes of $\varphi$, respectively, as in (2.8).

For $\varphi \in \dot{L}^2$, let the rotating operator be $\mathcal {J}\varphi := \varphi ^\perp = (-\varphi _2 , \varphi _1)^\top .$ Denote the Leray projection in ${\mathbb {T}}^2$ by

2.12 $$\begin{aligned} \mathfrak {P}_h \overline{\varphi } := \overline{\varphi } - \nabla \Delta _h^{-1} \nabla \cdot \overline{\varphi }. \end{aligned}$$

Here, $\Delta _h^{-1}$ represents the inverse of Laplacian operator in ${\mathbb {T}}^2$ with zero mean value. We define the analogy of the Leray projection for the PEs ${\mathfrak {P}}_p : \dot{L}^2\rightarrow \mathcal {S}$ as

$$\begin{aligned} {\mathfrak {P}}_p \varphi := \widetilde{\varphi } + \mathfrak {P}_h \overline{\varphi }. \end{aligned}$$

Moreover, let ${\mathfrak {R}}: \mathcal {S}\rightarrow \mathcal {S}$ be defined as

$$\begin{aligned} {\mathfrak {R}} \varphi := {\mathfrak {P}}_p (\mathcal {J}\varphi ) . \end{aligned}$$

With notations as above, a direct computation shows that

$$\begin{aligned} {\mathfrak {R}} \varphi = \widetilde{\varphi }^\perp \qquad \text {for} \quad \varphi \in {\mathcal {S}}. \end{aligned}$$

Indeed, owing to (2.11), $\varphi = \nabla ^\perp \psi (\varvec{x}) + \widetilde{\varphi } \in {\mathcal {S}}$ for some $\psi (\varvec{x})$. Then

$$\begin{aligned} \begin{aligned} {\mathfrak {R}} \varphi =&{\mathfrak {P}}_p({\mathcal {J}} {\widetilde{\varphi }} ) + {\mathfrak {P}}_p({\mathcal {J}} \nabla ^\perp \psi (\varvec{x})) \\ =&\widetilde{\varphi }^\perp - \underbrace{{\mathfrak {P}}_h \nabla \psi (\varvec{x})}_{\equiv 0} = \widetilde{\varphi }^\perp . \end{aligned} \end{aligned}$$

Therefore, the kernel of ${\mathfrak {R}}$ is given by

2.13 $$\begin{aligned} \ker {\mathfrak {R}} = \Big \{\varphi \in \mathcal {S}: \widetilde{\varphi }^\perp = 0 \Big \} = \Big \{\varphi \in \mathcal {S}: \varphi = \overline{\varphi } \Big \}. \end{aligned}$$

One can define the projection ${\mathfrak {P}}_0: \mathcal {S} \rightarrow \ker {\mathfrak {R}}$ by

2.14 $$\begin{aligned} {\mathfrak {P}}_0 \varphi := \overline{\varphi } = \int _0^1 \varphi (\varvec{x},z) dz. \end{aligned}$$

Notice that ${\mathfrak {P}}_0$ can be interpreted as projection to the barotropic mode. The fact that $\ker {\mathfrak {R}}$ coincides with the space of functions with only the barotropic mode plays an important role in our analysis.

Furthermore, let

2.15 $$\begin{aligned} {\mathfrak {P}}_+ \varphi := \frac{1}{2}(\widetilde{\varphi } + i\widetilde{\varphi }^\perp ), \qquad \text {and} \qquad {\mathfrak {P}}_- \varphi := \frac{1}{2}(\widetilde{\varphi } - i\widetilde{\varphi }^\perp ). \end{aligned}$$

Then it is easy to verify that

$$\begin{aligned} {\mathfrak {R}} {\mathfrak {P}}_\pm \varphi = \mp i {\mathfrak {P}}_\pm \varphi , \end{aligned}$$

i.e., ${\mathfrak {P}}_\pm$ are the projection operators to eigenspaces of ${\mathfrak {R}}$ with eigenvalues $\mp i$, respectively.

Similarly to [18, 23, 33], Lemma 2.2–2.3, below, summarize projection properties of ${\mathfrak {P}}_0, {\mathfrak {P}}_\pm$. For the proof, we refer readers to [23] for details.

#### Lemma 2.2

For any $\varphi \in L^2(\mathcal {D})$, we have the following decomposition:

2.16 $$\begin{aligned} \varphi = {\mathfrak {P}}_0 \varphi + {\mathfrak {P}}_+ \varphi + \mathfrak P_- \varphi . \end{aligned}$$

Moreover, we have the following properties:

$$\begin{aligned} {\mathfrak {P}}_\pm {\mathfrak {P}}_\pm \varphi = {\mathfrak {P}}_\pm \varphi , \qquad {\mathfrak {P}}_0 {\mathfrak {P}}_0 \varphi = {\mathfrak {P}}_0 \varphi ,\qquad \text {and} \qquad 0 \equiv {\mathfrak {P}}_\pm \mathfrak P_\mp \varphi = {\mathfrak {P}}_0 {\mathfrak {P}}_\pm \varphi = \mathfrak P_\pm {\mathfrak {P}}_0 \varphi . \end{aligned}$$

#### Lemma 2.3

For $f,g \in L^2(\mathcal {D})$, we have

$$\begin{aligned} \langle {\mathfrak {P}}_0 f, g \rangle = \langle f, {\mathfrak {P}}_0 g\rangle = \langle {\mathfrak {P}}_0 f, {\mathfrak {P}}_0 g\rangle \qquad \text {and} \qquad \langle {\mathfrak {P}}_\pm f, g\rangle = \langle f, {\mathfrak {P}}_\pm g\rangle . \end{aligned}$$

Here the $L^2$ inner product is defined as (2.1). Moreover, if $f\in \mathcal {S}_{r,s,\tau }$ with $r, s, \tau \ge 0$, $s \in {\mathbb {Z}}$, we have

$$\begin{aligned} A^{r}e^{\tau A} \partial _z^s {\mathfrak {P}}_0 f = {\mathfrak {P}}_0 A^{r}e^{\tau A} \partial _z^s f \;\;\; \text {and}\;\;\; A^{r}e^{\tau A} \partial _z^s {\mathfrak {P}}_\pm f = {\mathfrak {P}}_\pm A^{r}e^{\tau A} \partial _z^s f. \end{aligned}$$

Let ${\mathfrak {I}}$ be the identity operator. A direct corollary of Lemma 2.3 is the following:

#### Corollary 2.1

Consider $r\ge 0, \tau \ge 0$, and $s \in {\mathbb {Z}}_+$. Since $\mathcal {V} = {\mathfrak {P}}_0 \mathcal {V} + ({\mathfrak {I}}-\mathfrak P_0)\mathcal {V} = \overline{\mathcal {V}} + \widetilde{\mathcal {V}}$, we have

$$\begin{aligned} \Vert \mathcal {V}\Vert ^2 = \Vert \overline{\mathcal {V}}\Vert ^2 + \Vert \widetilde{\mathcal {V}}\Vert ^2, \qquad \Vert \partial _z^s \mathcal {V}\Vert ^2 = \Vert \partial _z^s \widetilde{\mathcal {V}}\Vert ^2, \end{aligned}$$

and

$$\begin{aligned} \Vert A^r e^{\tau A}\mathcal {V}\Vert ^2 = \Vert A^r e^{\tau A}\overline{\mathcal {V}}\Vert ^2 + \Vert A^r e^{\tau A} \widetilde{\mathcal {V}}\Vert ^2, \qquad \Vert A^r e^{\tau A} \partial _z^s \mathcal {V}\Vert ^2 = \Vert A^r e^{\tau A}\partial _z^s \widetilde{\mathcal {V}}\Vert ^2. \end{aligned}$$

Moreover, after applying ${\mathfrak {P}}_0$ and ${\mathfrak {I}}-\mathfrak P_0$ to equation (1.1a), thanks to (1.3), (2.9), and (2.10), one can derive the evolutionary equations for $\overline{\mathcal {V}}$ and $\widetilde{\mathcal {V}}$ as follows:

2.17a $$\begin{aligned}&\partial _t \overline{\mathcal {V}} + \overline{\mathcal {V}}\cdot \nabla \overline{\mathcal {V}} + {\mathfrak {P}}_0 \Big ((\nabla \cdot \widetilde{\mathcal {V}}) \widetilde{\mathcal {V}} + \widetilde{\mathcal {V}}\cdot \nabla \widetilde{\mathcal {V}} \Big ) + \nabla p = 0, \end{aligned}$$

2.17b $$\begin{aligned}&\partial _t \widetilde{\mathcal {V}} + \widetilde{\mathcal {V}} \cdot \nabla \widetilde{\mathcal {V}} + \widetilde{\mathcal {V}} \cdot \nabla \overline{\mathcal {V}} + \overline{\mathcal {V}} \cdot \nabla \widetilde{\mathcal {V}} - {\mathfrak {P}}_0\Big (\widetilde{\mathcal {V}} \cdot \nabla \widetilde{\mathcal {V}} + (\nabla \cdot \widetilde{\mathcal {V}}) \widetilde{\mathcal {V}} \Big )\nonumber \\&\qquad - \Big (\int _0^z \nabla \cdot \widetilde{\mathcal {V}}(\varvec{x},s)ds \Big ) \partial _z \widetilde{\mathcal {V}} + \Omega \widetilde{\mathcal {V}}^{\perp } - \nu \partial _{zz} \widetilde{\mathcal {V}} = 0. \end{aligned}$$

Here, we have abused the notation by denoting $p - \Omega \psi$ with $\nabla ^\perp \psi (\varvec{x},t) = \overline{\mathcal V}(\varvec{x},t)$ as *p*, where $\psi$ is the stream function of ${\mathcal {V}}$ (see (2.11)).

#### Remark 3

According to (2.13), (2.17) can be viewed as the orthogonal decomposition of (1.1) into $\ker {\mathfrak {R}}$ and $(\ker {\mathfrak {R}})^\perp$. As $|\Omega | \rightarrow \infty$, formal asymptotic analysis of (2.17b) assures that, for well-prepared data (i.e., data ensuring that (2.17b) makes sense), $\widetilde{{\mathcal {V}}} \rightarrow 0$ in some functional space. Therefore, in the limiting equations, (2.17) converge to the 2*D* Euler equations at leading order. In particular, in [23], it has been shown that the lifespan of the solutions can be prolonged with well-prepared initial data in the inviscid case.

According to (2.15), one has $\widetilde{\mathcal V}^\perp = -i {\mathfrak {P}}_+ {\mathcal {V}} + i {\mathfrak {P}}_- {\mathcal {V}}$. Therefore, after applying ${\mathfrak {P}}_\pm$ to (2.17b), we arrive at

2.18 $$\begin{aligned} \begin{aligned} \partial _t {\mathfrak {P}}_\pm \mathcal {V} + {\mathfrak {P}}_\pm \Big (&\widetilde{\mathcal {V}} \cdot \nabla \widetilde{\mathcal {V}} + \widetilde{\mathcal {V}} \cdot \nabla \overline{\mathcal {V}} + \overline{\mathcal {V}} \cdot \nabla \widetilde{\mathcal {V}} - {\mathfrak {P}}_0(\widetilde{\mathcal {V}} \cdot \nabla \widetilde{\mathcal {V}} + (\nabla \cdot \widetilde{\mathcal {V}}) \widetilde{\mathcal {V}} )\\&- (\int _0^z \nabla \cdot \widetilde{\mathcal {V}}(\varvec{x},s)ds ) \partial _z \widetilde{\mathcal {V}} \Big ) \mp i\Omega {\mathfrak {P}}_\pm \mathcal {V} - \nu \partial _{zz} {\mathfrak {P}}_\pm \mathcal {V} = 0 . \end{aligned} \end{aligned}$$

Let

2.19 $$\begin{aligned} \mathcal {V}_+ := e^{-i\Omega t}{\mathfrak {P}}_+ \mathcal {V} \qquad \text {and} \qquad \mathcal {V}_- := e^{i\Omega t} {\mathfrak {P}}_- \mathcal {V} . \end{aligned}$$

Then, for $r \ge 0, \tau \ge 0, s \ge 0$, and $s \in {\mathbb {Z}}$, it is straightforward to check that,

2.20 $$\begin{aligned} \Vert A^r e^{\tau A} \partial _z^s \mathcal {V}_+\Vert ^2 = \Vert A^r e^{\tau A} \partial _z^s \mathcal {V}_-\Vert ^2 = \frac{1}{2}\Vert A^r e^{\tau A} \partial _z^s \widetilde{\mathcal {V}}\Vert ^2. \end{aligned}$$

One can derive from (2.18) that

2.21 $$\begin{aligned} \begin{aligned} \partial _t \mathcal {V}_\pm + e^{\mp i\Omega t}{\mathfrak {P}}_\pm \Big (&\widetilde{\mathcal {V}} \cdot \nabla \widetilde{\mathcal {V}} + \widetilde{\mathcal {V}} \cdot \nabla \overline{\mathcal {V}} + \overline{\mathcal {V}} \cdot \nabla \widetilde{\mathcal {V}} - {\mathfrak {P}}_0(\widetilde{\mathcal {V}} \cdot \nabla \widetilde{\mathcal {V}} + (\nabla \cdot \widetilde{\mathcal {V}}) \widetilde{\mathcal {V}} )\\&- \left( \int _0^z \nabla \cdot \widetilde{\mathcal {V}}(\varvec{x},s)ds \right) \partial _z \widetilde{\mathcal {V}} \Big ) - \nu \partial _{zz} \mathcal {V}_\pm = 0. \end{aligned} \end{aligned}$$

Thanks to Lemma 2.2 and (2.15), we have

$$\begin{aligned}&{\mathfrak {P}}_+(\widetilde{\mathcal {V}} \cdot \nabla \widetilde{\mathcal {V}})=\frac{1}{2} (\widetilde{\mathcal {V}} \cdot \nabla \widetilde{\mathcal {V}} + i \widetilde{\mathcal {V}} \cdot \nabla \widetilde{\mathcal {V}}^\perp ) - \frac{1}{2} {\mathfrak {P}}_0\Big ( \widetilde{\mathcal {V}} \cdot \nabla \widetilde{\mathcal {V}} + i \widetilde{\mathcal {V}} \cdot \nabla \widetilde{\mathcal {V}}^\perp \Big )\\&= \frac{1}{2} \widetilde{\mathcal {V}} \cdot \nabla (\widetilde{\mathcal {V}} + i\widetilde{\mathcal {V}}^\perp ) - \frac{1}{2}{\mathfrak {P}}_0\Big ( \widetilde{\mathcal {V}} \cdot \nabla (\widetilde{\mathcal {V}} + i\widetilde{\mathcal {V}}^\perp ) \Big ) = e^{i\Omega t}\Big (\widetilde{\mathcal {V}} \cdot \nabla \mathcal {V}_+ - {\mathfrak {P}}_0(\widetilde{\mathcal {V}} \cdot \nabla \mathcal {V}_+) \Big ) ,\\&{\mathfrak {P}}_+(\widetilde{\mathcal {V}} \cdot \nabla \overline{\mathcal {V}}) = \frac{1}{2} (\widetilde{\mathcal {V}} \cdot \nabla \overline{\mathcal {V}} + i \widetilde{\mathcal {V}} \cdot \nabla \overline{\mathcal {V}}^\perp ) = \frac{1}{2} \widetilde{\mathcal {V}} \cdot \nabla (\overline{\mathcal {V}} + i\overline{\mathcal {V}}^\perp ) ,\\&{\mathfrak {P}}_+(\overline{\mathcal {V}} \cdot \nabla \widetilde{\mathcal {V}}) = \frac{1}{2} (\overline{\mathcal {V}} \cdot \nabla \widetilde{\mathcal {V}} + i \overline{\mathcal {V}} \cdot \nabla \widetilde{\mathcal {V}}^\perp ) = e^{i\Omega t}(\overline{\mathcal {V}} \cdot \nabla \mathcal {V}_+ ),\\&{\mathfrak {P}}_+{\mathfrak {P}}_0\Big (\widetilde{\mathcal {V}} \cdot \nabla \widetilde{\mathcal {V}} +(\nabla \cdot \widetilde{\mathcal {V}}) \widetilde{\mathcal {V}}\Big ) = 0. \end{aligned}$$

After applying integration by parts, one has

$$\begin{aligned} \begin{aligned} {\mathfrak {P}}_+\Big ((\int _0^z \nabla \cdot \widetilde{\mathcal {V}}(\varvec{x},s)ds) \partial _z \widetilde{\mathcal {V}}\Big ) =&\frac{1}{2} \Big ((\int _0^z \nabla \cdot \widetilde{\mathcal {V}}(\varvec{x},s)ds ) \partial _z \widetilde{\mathcal {V}} + i (\int _0^z \nabla \cdot \widetilde{\mathcal {V}}(\varvec{x},s)ds ) \partial _z \widetilde{\mathcal {V}}^\perp \Big ) \\&- \frac{1}{2} {\mathfrak {P}}_0\Big ((\int _0^z \nabla \cdot \widetilde{\mathcal {V}}(\varvec{x},s)ds ) \partial _z \widetilde{\mathcal {V}} + i (\int _0^z \nabla \cdot \widetilde{\mathcal {V}}(\varvec{x},s)ds ) \partial _z \widetilde{\mathcal {V}}^\perp \Big )\\ =&e^{i\Omega t} (\int _0^z \nabla \cdot \widetilde{\mathcal {V}}(\varvec{x},s)ds ) \partial _z \mathcal {V}_+ + e^{i\Omega t} {\mathfrak {P}}_0 \Big ( (\nabla \cdot \widetilde{\mathcal {V}}) \mathcal {V}_+ \Big ). \end{aligned} \end{aligned}$$

Moreover, thanks to (2.15) and (2.19), $\widetilde{{\mathcal {V}}} = {\mathcal {V}}_+ e^{i\Omega t} + \mathcal V_-e^{-i\Omega t}$. Therefore, the $\mathcal {V}_+$ part of (2.21) can be written as

2.22 $$\begin{aligned} \begin{aligned} \partial _t \mathcal {V}_+ =&-e^{i\Omega t} \Big (\mathcal {V}_+ \cdot \nabla \mathcal {V}_+ - {\mathfrak {P}}_0( \mathcal {V}_+ \cdot \nabla \mathcal {V}_+ + (\nabla \cdot \mathcal {V}_+) \mathcal {V}_+) - (\int _0^z \nabla \cdot \mathcal {V}_+(\varvec{x},s)ds ) \partial _z \mathcal {V}_+ \Big ) \\&- \Big (\overline{\mathcal {V}} \cdot \nabla \mathcal {V}_+ + \frac{1}{2}(\mathcal {V}_+ \cdot \nabla )(\overline{\mathcal {V}} +i\overline{\mathcal {V}}^\perp ) \Big ) + \nu \partial _{zz} \mathcal {V}_+ - e^{-2i\Omega t} \frac{1}{2} (\mathcal {V}_- \cdot \nabla )(\overline{\mathcal {V}} +i\overline{\mathcal {V}}^\perp ) \\&- e^{-i\Omega t} \Big (\mathcal {V}_- \cdot \nabla \mathcal {V}_+ - {\mathfrak {P}}_0( \mathcal {V}_- \cdot \nabla \mathcal {V}_+ + (\nabla \cdot \mathcal {V}_-) \mathcal {V}_+) - (\int _0^z \nabla \cdot \mathcal {V}_-(\varvec{x},s)ds ) \partial _z \mathcal {V}_+ \Big ). \end{aligned} \end{aligned}$$

Similarly, the $\mathcal {V}_-$ part of (2.21) can be written as

2.23 $$\begin{aligned} \begin{aligned} \partial _t \mathcal {V}_- =&-e^{-i\Omega t} \Big (\mathcal {V}_- \cdot \nabla \mathcal {V}_- - {\mathfrak {P}}_0( \mathcal {V}_- \cdot \nabla \mathcal {V}_- + (\nabla \cdot \mathcal {V}_-) \mathcal {V}_-) - (\int _0^z \nabla \cdot \mathcal {V}_-(\varvec{x},s)ds ) \partial _z \mathcal {V}_- \Big ) \\&- \Big (\overline{\mathcal {V}} \cdot \nabla \mathcal {V}_- + \frac{1}{2}(\mathcal {V}_- \cdot \nabla )(\overline{\mathcal {V}} -i\overline{\mathcal {V}}^\perp ) \Big ) + \nu \partial _{zz} \mathcal {V}_- - e^{2i\Omega t} \frac{1}{2} (\mathcal {V}_+ \cdot \nabla )(\overline{\mathcal {V}} -i\overline{\mathcal {V}}^\perp ) \\&- e^{i\Omega t} \Big (\mathcal {V}_+ \cdot \nabla \mathcal {V}_- - {\mathfrak {P}}_0( \mathcal {V}_+ \cdot \nabla \mathcal {V}_- + (\nabla \cdot \mathcal {V}_+) \mathcal {V}_-) - (\int _0^z \nabla \cdot \mathcal {V}_+(\varvec{x},s)ds ) \partial _z \mathcal {V}_- \Big ). \end{aligned} \end{aligned}$$

In addition, (2.17a) can be written as

$$\begin{aligned} \begin{aligned} \partial _t \overline{\mathcal {V}} + \overline{\mathcal {V}}\cdot \nabla \overline{\mathcal {V}} + e^{2i\Omega t} {\mathfrak {P}}_0 \Big (\mathcal {V}_+ \cdot \nabla \mathcal {V}_+ + (\nabla \cdot \mathcal {V}_+) \mathcal {V}_+\Big ) + e^{-2i\Omega t} {\mathfrak {P}}_0 \Big (\mathcal {V}_- \cdot \nabla \mathcal {V}_- + (\nabla \cdot \mathcal {V}_-) \mathcal {V}_-\Big ) \\ + \nabla p + {\mathfrak {P}}_0 \Big (\mathcal {V}_+ \cdot \nabla \mathcal {V}_- + \mathcal {V}_- \cdot \nabla \mathcal {V}_+ + (\nabla \cdot \mathcal {V}_+) \mathcal {V}_- + (\nabla \cdot \mathcal {V}_-) \mathcal {V}_+ \Big ) = 0. \end{aligned} \end{aligned}$$

Recalling (2.15) and (2.19), i.e., $\mathcal {V}_\pm = e^{\mp i\Omega t} {\mathfrak {P}}_\pm \mathcal {V} = \frac{1}{2} e^{\mp i\Omega t} (\widetilde{\mathcal {V}} \pm i \widetilde{\mathcal {V}}^\perp )$, we rewrite the last term of the above equation as

$$\begin{aligned}&{\mathfrak {P}}_0 \Big (\mathcal {V}_+ \cdot \nabla \mathcal {V}_- + \mathcal {V}_- \cdot \nabla \mathcal {V}_+ + (\nabla \cdot \mathcal {V}_+) \mathcal {V}_- + (\nabla \cdot \mathcal {V}_-) \mathcal {V}_+ \Big ) \\&= \frac{1}{2} {\mathfrak {P}}_0 \Big (\widetilde{\mathcal {V}} \cdot \nabla \widetilde{\mathcal {V}} + \widetilde{\mathcal {V}}^\perp \cdot \nabla \widetilde{\mathcal {V}}^\perp + (\nabla \cdot \widetilde{\mathcal {V}}) \widetilde{\mathcal {V}} + (\nabla \cdot \widetilde{\mathcal {V}}^\perp ) \widetilde{\mathcal {V}}^\perp \Big ) = \frac{1}{2} {\mathfrak {P}}_0 (\nabla |\widetilde{\mathcal {V}}|^2) = \nabla (\frac{1}{2} {\mathfrak {P}}_0 |\widetilde{\mathcal {V}}|^2), \end{aligned}$$

which can be combined with $\nabla p$. Therefore, with abuse of notation, one can rewrite (2.17a) as

2.24 $$\begin{aligned} \begin{aligned} \partial _t \overline{\mathcal {V}} + (\overline{\mathcal {V}}\cdot \nabla \overline{\mathcal {V}}) + \nabla p&+ e^{2i\Omega t} {\mathfrak {P}}_0 \Big (\mathcal {V}_+ \cdot \nabla \mathcal {V}_+ + (\nabla \cdot \mathcal {V}_+) \mathcal {V}_+ \Big )\\ +&e^{-2i\Omega t} {\mathfrak {P}}_0\Big (\mathcal {V}_- \cdot \nabla \mathcal {V}_- + (\nabla \cdot \mathcal {V}_-) \mathcal {V}_-\Big ) = 0. \end{aligned} \end{aligned}$$

## Local Well-posedness

In sections 3.1 and 3.2, below, we will establish the local well-posedness, i.e., the existence, the uniqueness, and the continuous dependency on initial data, of weak solutions to system (1.1), defined as below:

### Definition 3.1

Let $\mathcal {T}>0$, $r>2$, $\tau _0>0$, and suppose that the initial data $\mathcal {V}_0\in \mathcal {S}_{r,0,\tau _0}\cap H$. We say $\mathcal {V}$ is a Leray-Hopf type weak solution to system (1.1) with initial and boundary conditions (1.2)–(1.3) if

1. there exists $\tau (t) > 0$, for $t \in [0,{\mathcal {T}}]$, such that
  $$\begin{aligned}&\mathcal {V}\in L^\infty \big (0,\mathcal {T}; \mathcal {S}_{r,0,\tau (t)} \big ) \cap L^2\big (0,\mathcal {T}; V\cap \mathcal {S}_{r,1,\tau (t)}\cap \mathcal {S}_{r+\frac{1}{2},0,\tau (t)}\big ),\\&\partial _t {\mathcal {V}} , A^{r-\frac{1}{2}} e^{\tau A} \partial _t \mathcal {V} \in L^2\big (0,\mathcal {T}; V'\big ), \end{aligned}$$
2. system (1.1) is satisfied in the distribution sense,
3. and moreover, the following energy inequality holds:
  $$\begin{aligned} \Vert \mathcal {V}(t)\Vert _{r,0,\tau (t)}^2 + 2 \int _0^t \Big ( \nu \Vert \partial _z \mathcal {V}(s)\Vert _{r,0,\tau (s)}^2 + \Vert A^{r+\frac{1}{2}} e^{\tau (s) A} \mathcal {V}(s)\Vert ^2 \Big ) ds\le \Vert \mathcal {V}_0\Vert _{r,0,\tau _0}^2. \end{aligned}$$

The following theorem is the main result in this section.

### Theorem 3.1

Assume $\mathcal {V}_0\in \mathcal {S}_{r,0,\tau _0}\cap H$ with $r>2$ and $\tau _0>0$. Let $\Omega \in \mathbb {R}$ be arbitrary and fixed. Then there exist a positive time ${\mathcal {T}}>0$ and a positive function $\tau (t) >0$ given in (3.6) and (3.5), below, respectively,

such that ${\mathcal {V}}$ is a Leray-Hopf type weak solution, as in Definition 3.1, to system (1.1) with (1.2) and (1.3) in $[0,\mathcal {T}]$. In particular, $\tau (t)$ and ${\mathcal {T}}$ are independent of $\Omega$. Moreover, $\mathcal {V}$ is unique and depends continuously on the initial data, in the sense of (3.21), below.

Notice that we do not need to assume (2.7) in Theorem 3.1. Throughout the rest of this section, we assume that $({\mathcal {V}}, p)$ satisfies (1.1)–(1.3) and is smooth enough such that the following calculation makes sense. The rigid justification can be established through Galerkin approximation arguments (see, e.g., [23, 39]). In particular, in section 3.1, we establish the *a priori* estimates of solutions to system (1.1) with (1.3). In section 3.2, we finish the proof of Theorem 3.1 by establishing the uniqueness and continuous dependency on initial data. In section 3.3, we show that the weak solution immediately becomes analytic in *z*, and the radius of analyticity in *z* increases as long as the solution exists.

### *A Priori* Estimates

Direct calculation of $\langle (1.1a), {\mathcal {V}} \rangle + \langle A^r e^{\tau A} (1.1a),A^r e^{\tau A} {\mathcal {V}}\rangle$, after applying integration by parts, (1.1c), and (1.3), shows that

3.1 $$\begin{aligned} \begin{aligned} \frac{1}{2}\frac{d}{dt} \Vert \mathcal {V}\Vert _{r,0,\tau }^2 +&\nu \Vert \partial _z \mathcal {V}\Vert _{r,0,\tau }^2 - \dot{\tau } \Vert A^{r+\frac{1}{2}} e^{\tau A} \mathcal {V}\Vert ^2 = -\Big \langle A^r e^{\tau A}(\mathcal {V}\cdot \nabla \mathcal {V}), A^r e^{\tau A} \mathcal {V}\Big \rangle \\ +&\Big \langle A^r e^{\tau A} \Big [ \Big (\int _0^z \nabla \cdot \mathcal {V}(\varvec{x},s)ds \Big ) \partial _z \mathcal {V} \Big ], A^r e^{\tau A} \mathcal {V} \Big \rangle =: I_1 + I_2. \end{aligned} \end{aligned}$$

By virtue of Lemma A.1, the Sobolev inequality, and the Hölder inequality, we have

$$\begin{aligned} \begin{aligned} |I_1|&\le \Big |\Big \langle A^r e^{\tau A} ( \mathcal {V}\cdot \nabla \mathcal {V}), A^r e^{\tau A} \mathcal {V} \Big \rangle \Big | \\&\le \int _0^1 C_r \Big (\Vert A^r e^{\tau A} \mathcal {V}(z)\Vert _{L^2 (\mathbb {T}^2)} + \Vert \mathcal {V}(z)\Vert _{L^2(\mathbb {T}^2)} \Big ) \Vert A^{r+\frac{1}{2}} e^{\tau A} \mathcal {V}(z)\Vert _{L^2(\mathbb {T}^2)}^2 dz\\&\le C_r (\Vert \mathcal {V}\Vert _{r,0,\tau } + \Vert \partial _z \mathcal {V}\Vert _{r,0,\tau }) \Vert A^{r+\frac{1}{2}} e^{\tau A} \mathcal {V}\Vert ^2. \end{aligned} \end{aligned}$$

Applying Lemma A.2 to $I_2$ leads to

$$\begin{aligned} |I_2| \le C_r \Vert \partial _z \mathcal {V}\Vert _{r,0,\tau } \Vert A^{r+\frac{1}{2}} e^{\tau A} \mathcal {V}\Vert ^2. \end{aligned}$$

Thus from (3.1), one has

3.2 $$\begin{aligned} \begin{aligned} \frac{1}{2}\frac{d}{dt} \Vert \mathcal {V}\Vert _{r,0,\tau }^2 + \nu \Vert \partial _z \mathcal {V}\Vert _{r,0,\tau }^2 + \Vert A^{r+\frac{1}{2}} e^{\tau A} \mathcal {V}\Vert ^2 \le \Big (\dot{\tau }+1+C_r (\Vert \mathcal {V}\Vert _{r,0,\tau } + \Vert \partial _z \mathcal {V}\Vert _{r,0,\tau })\Big ) \\ \qquad \times \Vert A^{r+\frac{1}{2}} e^{\tau A} \mathcal {V}\Vert ^2 \le \Big (\dot{\tau }+C_r (1+\Vert \mathcal {V}\Vert ^2_{r,0,\tau } + \Vert \partial _z \mathcal {V}\Vert ^2_{r,0,\tau })\Big ) \Vert A^{r+\frac{1}{2}} e^{\tau A} \mathcal {V}\Vert ^2 . \end{aligned} \end{aligned}$$

Choose $\tau$ such that

3.3 $$\begin{aligned} \dot{\tau }+1+C_r (\Vert \mathcal {V}\Vert _{r,0,\tau } + \Vert \partial _z \mathcal {V}\Vert _{r,0,\tau }) = 0 . \end{aligned}$$

Then, one has

$$\begin{aligned} \frac{1}{2}\frac{d}{dt} \Vert \mathcal {V}\Vert _{r,0,\tau }^2 + \nu \Vert \partial _z \mathcal {V}\Vert _{r,0,\tau }^2 + \Vert A^{r+\frac{1}{2}} e^{\tau A} \mathcal {V}\Vert ^2 \le 0. \end{aligned}$$

For ${\mathcal {T}} > 0$, to be determined, and $t \in [0, \mathcal {T} ]$, one has, after integrating (3.2) in the *t*-variable,

3.4 $$\begin{aligned} \Vert \mathcal {V}(t)\Vert _{r,0,\tau (t)}^2 + 2 \int _0^t \Big (\nu \Vert \partial _z \mathcal {V}(s)\Vert _{r,0,\tau (s)}^2 + \Vert A^{r+\frac{1}{2}} e^{\tau (s) A} \mathcal {V}(s)\Vert ^2 \Big ) ds\le \Vert \mathcal {V}_0\Vert _{r,0,\tau _0}^2. \end{aligned}$$

On the other hand, integrating (3.3) yields

3.5 $$\begin{aligned} \begin{aligned} \tau (t) =&\tau _0 - t - C_r \int _0^t \bigl ( \Vert \mathcal {V}(s)\Vert _{r,0,\tau (s)} + \Vert \partial _z \mathcal {V}(s)\Vert _{r,0,\tau (s)} \bigr ) ds\\ \ge&\tau _0 - (1+C_r \Vert \mathcal {V}_0\Vert _{r,0,\tau _0}) t - \frac{C_r}{\sqrt{2\nu }} \Vert \mathcal {V}_0\Vert _{r,0,\tau _0} \sqrt{t}. \end{aligned} \end{aligned}$$

Consider, for $C_r > 0$ as in (3.5), that

3.6 $$\begin{aligned} \mathcal {T} := \Big (\frac{\sqrt{\frac{C_r^2 \Vert \mathcal {V}_0\Vert _{r,0,\tau _0}^2}{2\nu } + 2\tau _0(1+C_r \Vert \mathcal {V}_0\Vert _{r,0,\tau _0})} - \frac{C_r \Vert \mathcal {V}_0\Vert _{r,0,\tau _0}}{\sqrt{2\nu }}}{2(1+C_r \Vert \mathcal {V}_0\Vert _{r,0,\tau _0})}\Big )^2 > 0, \end{aligned}$$

which solves

$$\begin{aligned} (1+C_r \Vert \mathcal {V}_0\Vert _{r,0,\tau _0}) \mathcal {T} - \frac{C_r}{\sqrt{2\nu }} \Vert \mathcal {V}_0\Vert _{r,0,\tau _0} \sqrt{\mathcal {T}} = \frac{\tau _0}{2}. \end{aligned}$$

Then one has

$$\begin{aligned} \tau (t) \ge \tau _0/2 > 0 \quad \text {for} \quad t \in [0,{\mathcal {T}}]. \end{aligned}$$

Consequently, (3.4) implies that

3.7 $$\begin{aligned} \mathcal {V}\in L^\infty \big (0,\mathcal {T}; \mathcal {S}_{r,0,\tau (t)}\big ) \cap L^2\big (0,\mathcal {T}; V\cap \mathcal {S}_{r,1,\tau (t)}\cap \mathcal {S}_{r+\frac{1}{2},0,\tau (t)}\big ) \end{aligned}$$

with ${\mathcal {T}} > 0$ given as in (3.6) and $\tau (t)$ given as in (3.5) (or equivalently (3.3)).

Next, in order to obtain the estimate of $\partial _t \mathcal {V}$, testing (1.1a) with $\forall \phi \in \mathscr {V}$ (see (2.5)) leads to

3.8 $$\begin{aligned}&\Big \langle \partial _t \mathcal {V}, \phi \Big \rangle + \Big \langle \mathcal {V} \cdot \nabla \mathcal {V} - \Big (\int _0^z \nabla \cdot \mathcal {V}(\varvec{x},s)ds \Big ) \partial _z \mathcal {V} + \Omega \mathcal {V}^\perp - \nu \partial _{zz}\mathcal {V}, \phi \Big \rangle = 0 . \end{aligned}$$

where we have substituted, thanks to (1.1b) and (2.5), $\langle \nabla p , \phi \rangle = - \langle p , \nabla \cdot \phi \rangle = 0$. Since $r>2$, thanks to the Hölder inequality and the Sobolev inequality, we obtain that

$$\begin{aligned} \Big | \Big \langle \mathcal {V} \cdot \nabla \mathcal {V} , \phi \Big \rangle \Big | \le C \Vert \mathcal {V}\Vert _{L^\infty _{\varvec{x}}L^2_z} \Vert \nabla \mathcal {V}\Vert _{L^2_{\varvec{x}}L^2_z} \Vert \phi \Vert _{L^2_{\varvec{x}}L^\infty _z} \le C_r \Vert \mathcal {V}\Vert _{r,0,\tau }^2 \Vert \phi \Vert _{V} \end{aligned}$$

and

$$\begin{aligned} \begin{aligned}&\Big | \Big \langle \Big (\int _0^z \nabla \cdot \mathcal {V} (\varvec{x},s)ds \Big ) \partial _z \mathcal {V} , \phi \Big \rangle \Big | \le \int _{\mathbb {T}^2} \Big (\int _0^1 |\nabla \cdot \mathcal {V}| dz\Big ) \Big (\int _0^1 |\partial _z \mathcal {V}||\phi | dz\Big ) d \varvec{x} \\ \le C&\int _{\mathbb {T}^2} \Vert \nabla \mathcal {V}\Vert _{L^2_z} \Vert \partial _z \mathcal {V}\Vert _{L^2_z} \Vert \phi \Vert _{L^2_z} d\varvec{x} \le C \Vert \nabla \mathcal {V}\Vert _{L^2_{\varvec{x}}L^2_z}\Vert \partial _z \mathcal {V}\Vert _{L^4_{\varvec{x}}L^2_z}\Vert \phi \Vert _{L^4_{\varvec{x}}L^2_z} \le C_r \Vert \mathcal {V}\Vert _{r,1,\tau } \Vert \mathcal {V}\Vert _{r,0,\tau } \Vert \phi \Vert _{V}. \end{aligned} \end{aligned}$$

After applying integration by parts, one has

$$\begin{aligned} \Big |\Big \langle \Omega \mathcal {V}^\perp - \nu \partial _{zz}\mathcal {V}, \phi \Big \rangle \Big | = \Big |\Big \langle \Omega \mathcal {V}^\perp , \phi \Big \rangle + \nu \Big \langle \partial _z {\mathcal {V}}, \partial _z \phi \Big \rangle \Big | \le C_{\nu , \Omega } \Vert \mathcal {V}\Vert _{r,1,\tau }\Vert \phi \Vert _{V}. \end{aligned}$$

Therefore, one has

$$\begin{aligned}&\Big |\Big \langle \partial _t \mathcal {V}, \phi \Big \rangle \Big | \le C_{\nu ,r,\Omega } \Big (\Vert \mathcal {V}\Vert _{r,0,\tau }^2 + (1+\Vert \mathcal {V}\Vert _{r,0,\tau })\Vert \mathcal {V}\Vert _{r,1,\tau } \Big ) \Vert \phi \Vert _{V}. \end{aligned}$$

Since $\mathscr {V}$ is dense in *V*, one has

$$\begin{aligned} \Vert \partial _t \mathcal {V}\Vert _{V'} = \sup \limits _{\Vert \phi \Vert _{V}=1} \Big |\Big \langle \partial _t \mathcal {V}, \phi \Big \rangle \Big | \le C_{\nu ,r,\Omega } \Big (\Vert \mathcal {V}\Vert _{r,0,\tau }^2 + (1+\Vert \mathcal {V}\Vert _{r,0,\tau })\Vert \mathcal {V}\Vert _{r,1,\tau } \Big ) . \end{aligned}$$

Thanks to (3.7), we have

3.9 $$\begin{aligned} \begin{aligned}&\partial _t \mathcal {V}\in L^2(0,\mathcal {T}; V') \qquad \text {and}\\&\Vert \partial _t {\mathcal {V}} \Vert _{L^2(0,\mathcal {T}; V')} \le C_{\nu ,r,\Omega } \Big (C_{{\mathcal {T}}}\Vert \mathcal {V}\Vert _{L^\infty (0,{\mathcal {T}}; {\mathcal {S}}_{r,0,\tau })}^2 + (1+\Vert \mathcal {V}\Vert _{L^\infty (0,{\mathcal {T}}; {\mathcal {S}}_{r,0,\tau })})\Vert \mathcal {V}\Vert _{L^2(0,{\mathcal {T}}; {\mathcal {S}}_{r,1,\tau })} \Big ) < \infty . \end{aligned} \end{aligned}$$

Meanwhile, for $A^{r-\frac{1}{2}} e^{\tau A}\partial _t \mathcal {V}$, one has, similarly as in (3.8),

$$\begin{aligned} \begin{aligned} \Big \langle A^{r-\frac{1}{2}} e^{\tau A}\partial _t \mathcal {V}, \phi \Big \rangle + \Big \langle&A^{r-\frac{1}{2}} e^{\tau A}\big (\mathcal {V} \cdot \nabla \mathcal {V}\big ) - A^{r-\frac{1}{2}} e^{\tau A} \Big (\big (\int _0^z \nabla \cdot \mathcal {V}(\varvec{x},s)ds \big ) \partial _z \mathcal {V}\Big ) \\&+ \Omega A^{r-\frac{1}{2}} e^{\tau A}\mathcal {V}^\perp - \nu \partial _{zz}A^{r-\frac{1}{2}} e^{\tau A}\mathcal {V} , \phi \Big \rangle = 0 . \end{aligned} \end{aligned}$$

With $r>2$, thanks to Lemma 2.1, the Hölder inequality, and the Sobolev inequality, we obtain that

$$\begin{aligned} \begin{aligned} \Big | \Big \langle A^{r-\frac{1}{2}} e^{\tau A}\big (\mathcal {V} \cdot \nabla \mathcal {V}\big ), \phi \Big \rangle \Big |&\le \Vert A^{r-\frac{1}{2}} e^{\tau A}\mathcal {V} \cdot \nabla \mathcal {V}\Vert _{L^2_{\varvec{x}}L^1_z} \Vert \phi \Vert _{L^2_{\varvec{x}} L^\infty _z} \\&\le C_r \Vert \mathcal {V}\Vert _{r+\frac{1}{2},0,\tau } \Vert \mathcal {V} \Vert _{r,0,\tau } \Vert \phi \Vert _{V}. \end{aligned} \end{aligned}$$

After applying integration by parts in the *z*-variable and the Hölder inequality, one has

$$\begin{aligned} \begin{aligned}&\Big | \Big \langle A^{r-\frac{1}{2}} e^{\tau A} \Big (\big (\int _0^z \nabla \cdot \mathcal {V}(\varvec{x},s)ds \big ) \partial _z \mathcal {V}\Big ) , \phi \Big \rangle \Big | \le \Big | \Big \langle A^{r-\frac{1}{2}} e^{\tau A} \Big ( (\nabla \cdot \mathcal {V}) \mathcal {V} \Big ), \phi \Big \rangle \Big |\\&\qquad \qquad + \Big | \Big \langle A^{r-\frac{1}{2}} e^{\tau A} \Big ( \big (\int _0^z \nabla \cdot \mathcal {V}(\varvec{x},s)ds \big ) \mathcal {V} \Big ) , \partial _z \phi \Big \rangle \Big | \le C_r \Vert \mathcal {V}\Vert _{r+\frac{1}{2},0,\tau } \Vert \mathcal {V}\Vert _{r,0,\tau } \Vert \phi \Vert _{V}, \end{aligned} \end{aligned}$$

and similarly,

$$\begin{aligned} \begin{aligned} \Big | \Big \langle \Omega A^{r-\frac{1}{2}} e^{\tau A}\mathcal {V}^\perp - \nu \partial _{zz}A^{r-\frac{1}{2}} e^{\tau A}\mathcal {V} , \phi \Big \rangle \Big | \le C_{\nu , \Omega } \Vert \mathcal {V}\Vert _{r,1,\tau }\Vert \phi \Vert _{V}. \end{aligned} \end{aligned}$$

Therefore, one has

$$\begin{aligned}&\Big |\Big \langle A^{r-\frac{1}{2}} e^{\tau A}\partial _t \mathcal {V}, \phi \Big \rangle \Big | \le C_{\nu ,r,\Omega } \Big ( \Vert \mathcal {V}\Vert _{r+\frac{1}{2},0,\tau } \Vert \mathcal {V}\Vert _{r,0,\tau } + \Vert \mathcal {V}\Vert _{r,1,\tau } \Big ) \Vert \phi \Vert _{V}. \end{aligned}$$

Since $\mathscr {V}$ is dense in *V*, one has

$$\begin{aligned} \Vert A^{r-\frac{1}{2}} e^{\tau A}\partial _t \mathcal {V}\Vert _{V'} = \sup \limits _{\Vert \phi \Vert _{V}=1} \Big |\Big \langle A^{r-\frac{1}{2}} e^{\tau A}\partial _t \mathcal {V}, \phi \Big \rangle \Big | \le C_{\nu ,r,\Omega } \Big ( \Vert \mathcal {V}\Vert _{r+\frac{1}{2},0,\tau } \Vert \mathcal {V}\Vert _{r,0,\tau } + \Vert \mathcal {V}\Vert _{r,1,\tau } \Big ). \end{aligned}$$

Thanks to (3.7), we have

3.10 $$\begin{aligned} \begin{aligned} A^{r-\frac{1}{2}} e^{\tau A} \partial _t \mathcal {V}\in L^2\big (0,\mathcal {T}; V'\big ) \qquad \text {and}\qquad \\ \Vert A^{r-\frac{1}{2}} e^{\tau A} \partial _t \mathcal {V}\Vert _{L^2\big (0,\mathcal {T}; V'\big )} \le C_{\nu ,r,\Omega } \Big ( \Vert \mathcal {V}\Vert _{L^\infty (0,{\mathcal {T}}; {\mathcal {S}}_{r,0,\tau })} \Vert \mathcal {V}\Vert _{L^2(0,{\mathcal {T}}; {\mathcal {S}}_{r+\frac{1}{2},0,\tau })} + \Vert \mathcal {V}\Vert _{L^2(0,{\mathcal {T}}; {\mathcal {S}}_{r,1,\tau })} \Big )< \infty . \end{aligned} \end{aligned}$$

### Uniqueness and Continuous Dependence on the Initial Data

In this section, we show the uniqueness of solutions and the continuous dependence on the initial data. Let $\mathcal {V}_1$ and $\mathcal {V}_2$ be two weak solutions with initial data $(\mathcal {V}_0)_1$ and $(\mathcal {V}_0)_2$, respectively. Assume the radius of analyticity of $(\mathcal {V}_0)_1$ and $(\mathcal {V}_0)_2$ is $\tau _0$. By virtue of (3.5) and (3.6), for $i=1,2$, let

3.11 $$\begin{aligned} \begin{aligned} \tau _i(t) := \tau _0 - t - C_{r,i} \int _0^t \bigl ( \Vert \mathcal {V}_i(s) \Vert _{r,0,\tau _i(s)} + \Vert \partial _z \mathcal {V}_i(s)\Vert _{r,0,\tau _i(s)} \bigr ) ds, \\ \text {and} \qquad \mathcal {T}_i := \Big (\frac{\sqrt{\frac{C_{r,i} ^2 \Vert (\mathcal {V}_0)_i\Vert _{r,0,\tau _0}^2}{2\nu } + 2\tau _0(1+C_{r,i} \Vert (\mathcal {V}_0)_i\Vert _{r,0,\tau _0})} - \frac{C_{r,i} \Vert (\mathcal {V}_0)_i\Vert _{r,0,\tau _0}}{\sqrt{2\nu }}}{2(1+C_{r,i} \Vert (\mathcal {V}_0)_i\Vert _{r,0,\tau _0})}\Big )^2 \end{aligned} \end{aligned}$$

such that, according to (3.4), (3.7), (3.9), and (3.10),

$$\begin{aligned} \Vert \mathcal {V}_i(t)\Vert _{r,0,\tau _i(t)}^2 + 2 \int _0^t \Big ( \nu \Vert \partial _z \mathcal {V}_i(s)\Vert _{r,0,\tau _i(s)}^2 + \Vert A^{r+\frac{1}{2}} e^{\tau _i(s) A} \mathcal {V}_i(s)\Vert ^2 \Big ) ds \le \Vert (\mathcal {V}_0)_i\Vert ^2_{r,0,\tau _{i0}}, \end{aligned}$$

for $$t\in [0,\mathcal {T}_i]$$, and

$$\begin{aligned} \mathcal {V}_i\in L^\infty \big (0,\mathcal {T}_i; \mathcal {S}_{r,0,\tau _i(t)}\big ) \cap L^2\big (0,\mathcal {T}_i; V\cap \mathcal {S}_{r,1, \tau _i(t)}\cap \mathcal {S}_{r+\frac{1}{2},0,\tau _i(t)}\big ),\\ \partial _t \mathcal {V}_i \quad \text {and} \quad A^{r-\frac{1}{2}} e^{\tau _i A} \partial _t \mathcal {V}_i\in L^2\big (0,\mathcal {T}_i; V'\big ). \end{aligned}$$

We remind readers that $$ C_{r,i}, i =1,2, $$ are independent of $$ \Omega $$ and $$ \tau _0 $$.

Let

3.12 $$\begin{aligned} M := \max \Big \{ \Vert (\mathcal {V}_0)_1\Vert _{r,0,\tau _{0}}, \Vert (\mathcal {V}_0)_2\Vert _{r,0,\tau _{0}}\Big \}. \end{aligned}$$

Denote by $\delta \mathcal {V} := \mathcal {V}_1- \mathcal {V}_2$ and $\delta p := p_1 -p_2$. Let

3.13 $$\begin{aligned} \begin{aligned} \widetilde{\tau }(t) := \tau _0 - t - C_r \sum \limits _{i=1}^2\int _0^t \bigl ( \Vert \mathcal {V}_i(s)\Vert _{r,0,\tau _i(s)} + \Vert \mathcal {V}_i(s)\Vert _{r,0,\tau _i(s)}^2 + \Vert \partial _z \mathcal {V}_i(s)\Vert _{r,0,\tau _i(s)} \bigr ) ds, \\ \text {and} \quad \widetilde{\mathcal {T}} := \Big (\frac{\sqrt{\frac{2C_{r}^2 M^2}{\nu } + 2\tau _0\big (1+2C_{r} (M^2+M)\big )} - \frac{ \sqrt{2}C_{r} M}{\sqrt{\nu }}}{2\big (1+2C_{r} (M^2+M)\big )}\Big )^2, \end{aligned} \end{aligned}$$

where $C_r$ is a positive constant, to be determined later, satisfying

3.14 $$\begin{aligned} C_r \ge \max \lbrace C_{r,1}, C_{r,2}\rbrace . \end{aligned}$$

In particular, (3.13) and (3.14) imply that $\tilde{\tau }(t) \le \tau _i(t)$ and $\widetilde{{\mathcal {T}}} \le \widetilde{{\mathcal {T}}}_i$ for $i \in \lbrace 1, 2 \rbrace$ and $t \in (0,\widetilde{{\mathcal {T}}} ]$. Therefore, for $i=1,2$,

3.15 $$\begin{aligned} \delta \mathcal {V} \quad \text {and} \quad \mathcal {V}_i\in L^\infty \big (0, \widetilde{\mathcal {T}}; \mathcal {S}_{r,0,\widetilde{\tau }(t)}) \cap L^2\big (0,\widetilde{\mathcal {T}}; V\cap \mathcal {S}_{r,1,\widetilde{\tau }(t)}\cap \mathcal {S}_{r+\frac{1}{2},0,\widetilde{\tau }(t)}),\end{aligned}$$

3.16 $$\begin{aligned} \partial _t \delta \mathcal {V} \quad \text {and} \quad A^{r-\frac{1}{2}} e^{\widetilde{\tau } A} \partial _t \delta \mathcal {V}\in L^2\big (0, \widetilde{\mathcal {T}}; V'\big ), \end{aligned}$$

and

$$\begin{aligned} \Vert \mathcal {V}_i(t)\Vert _{r,0,\widetilde{\tau }(t)}^2 + 2 \int _0^t \Big ( \nu \Vert \partial _z \mathcal {V}_i(s)\Vert _{r,0,\widetilde{\tau }(s)}^2 + \Vert A^{r+\frac{1}{2}} e^{\widetilde{\tau }(s) A} \mathcal {V}_i(s)\Vert ^2 \Big ) ds \le M^2, \end{aligned}$$

for $$t\in [0,\widetilde{\mathcal {T}}]$$.

From system (1.1), it is clear that

$$\begin{aligned} \begin{aligned} \partial _t \delta \mathcal {V} + \delta \mathcal {V}\cdot \nabla \mathcal {V}_1 + \mathcal {V}_2\cdot \nabla \delta \mathcal {V} - \Big (\int _0^z \nabla \cdot \delta \mathcal {V}(\varvec{x},s)ds \Big ) \partial _z \mathcal {V}_1 - \Big (\int _0^z \nabla \cdot \mathcal {V}_2(\varvec{x},s)ds \Big ) \partial _z \delta \mathcal {V}\\ + \Omega \delta \mathcal {V}^\perp -\nu \partial _{zz} \delta \mathcal {V} + \nabla \delta p =0 \qquad \text {and} \qquad \partial _z \delta p = 0. \end{aligned} \end{aligned}$$

Notice that from (3.15), one has that $A^{r-\frac{1}{2}} e^{\widetilde{\tau } A} \delta \mathcal {V} \in L^2\big (0,\widetilde{\mathcal {T}}; V\big )$. Thanks to (3.16), similar calculation as in (3.1) leads to

3.17 $$\begin{aligned} \begin{aligned}&\frac{1}{2}\frac{d}{dt} \Vert \delta \mathcal {V}\Vert _{r-\frac{1}{2},0,\widetilde{\tau }}^2 + \nu \Vert \partial _z \delta \mathcal {V}\Vert _{r-\frac{1}{2},0,\widetilde{\tau }}^2 - \dot{\widetilde{\tau }} \Vert A^{r} e^{\widetilde{\tau } A} \delta \mathcal {V}\Vert ^2 \\&\quad = -\Big \langle \delta \mathcal {V}\cdot \nabla \mathcal {V}_1 + \mathcal {V}_2\cdot \nabla \delta \mathcal {V} - \Big (\int _0^z \nabla \cdot \delta \mathcal {V}(\varvec{x},s)ds \Big ) \partial _z \mathcal {V}_1 - \Big (\int _0^z \nabla \cdot \mathcal {V}_2(\varvec{x},s)ds \Big ) \partial _z \delta \mathcal {V} ,\delta \mathcal {V} \Big \rangle \\&\qquad -\Big \langle A^{r-\frac{1}{2}} e^{\widetilde{\tau } A}(\delta \mathcal {V}\cdot \nabla \mathcal {V}_1), A^{r-\frac{1}{2}} e^{\widetilde{\tau } A} \delta \mathcal {V}\Big \rangle +\Big \langle A^{r-\frac{1}{2}} e^{\widetilde{\tau } A} \Big [ \Big (\int _0^z \nabla \cdot \delta \mathcal {V}(\varvec{x},s)ds \Big ) \partial _z \mathcal {V}_1 \Big ], A^{r-\frac{1}{2}} e^{\widetilde{\tau } A} \delta \mathcal {V} \Big \rangle \\&\qquad -\Big \langle A^{r-\frac{1}{2}} e^{\widetilde{\tau } A}(\mathcal {V}_2\cdot \nabla \delta \mathcal {V}), A^{r-\frac{1}{2}} e^{\widetilde{\tau } A} \delta \mathcal {V}\Big \rangle +\Big \langle A^{r-\frac{1}{2}} e^{\widetilde{\tau } A} \Big [ \Big (\int _0^z \nabla \cdot \mathcal {V}_2(\varvec{x},s)ds \Big ) \partial _z \delta \mathcal {V} \Big ], A^{r-\frac{1}{2}} e^{\widetilde{\tau } A} \delta \mathcal {V} \Big \rangle . \end{aligned}\nonumber \\ \end{aligned}$$

After applying integration by parts, the Hölder inequality, the Young inequality, and the Sobolev inequality, since $r>2$, one has

$$\begin{aligned} \begin{aligned}&\Big | \Big \langle \delta \mathcal {V}\cdot \nabla \mathcal {V}_1 + \mathcal {V}_2\cdot \nabla \delta \mathcal {V} - \Big (\int _0^z \nabla \cdot \delta \mathcal {V}(\varvec{x},s)ds \Big ) \partial _z \mathcal {V}_1 - \Big (\int _0^z \nabla \cdot \mathcal {V}_2(\varvec{x},s)ds \Big ) \partial _z \delta \mathcal {V} ,\delta \mathcal {V} \Big \rangle \Big | \\&\quad = \Big | \Big \langle \delta \mathcal {V}\cdot \nabla \mathcal {V}_1 - \Big (\int _0^z \nabla \cdot \delta \mathcal {V}(\varvec{x},s)ds \Big ) \partial _z \mathcal {V}_1 ,\delta \mathcal {V} \Big \rangle \Big | \le C_{r-\frac{1}{2}} \Vert \mathcal {V}_1\Vert _{r,1,\widetilde{\tau }} \Vert \delta \mathcal {V}\Vert _{r-\frac{1}{2},0,\widetilde{\tau }}^2. \end{aligned} \end{aligned}$$

Thanks to Lemmas A.1 and A.2, the Hölder inequality, the Young inequality, and the Sobolev inequality, since $r>2$, one has

$$\begin{aligned}&\begin{aligned}&\Big | \Big \langle A^{r-\frac{1}{2}} e^{\widetilde{\tau } A}(\delta \mathcal {V}\cdot \nabla \mathcal {V}_1), A^{r-\frac{1}{2}} e^{\widetilde{\tau } A} \delta \mathcal {V}\Big \rangle \Big | \\&\quad \le \int _0^1 C_{r-\frac{1}{2}}\Big [ (\Vert A^{r-\frac{1}{2}} e^{\widetilde{\tau } A} \delta \mathcal {V}(z)\Vert _{L^2(\mathbb {T}^2)} + \Vert \delta \mathcal {V}(z)\Vert _{L^2(\mathbb {T}^2)} ) \Vert A^{r} e^{\widetilde{\tau } A} \mathcal {V}_1(z)\Vert _{L^2(\mathbb {T}^2)} \Vert A^{r} e^{\widetilde{\tau } A} \delta \mathcal {V}(z)\Vert _{L^2(\mathbb {T}^2)} \\&\qquad + \Vert A^{r} e^{\widetilde{\tau } A} \delta \mathcal {V}(z)\Vert _{L^2 (\mathbb {T}^2)} \Vert A^{r} e^{\widetilde{\tau } A} \mathcal {V}_1(z) \Vert _{L^2(\mathbb {T}^2)} \Vert A^{r-\frac{1}{2}} e^{\widetilde{\tau } A} \delta \mathcal {V}(z)\Vert _{L^2(\mathbb {T}^2)}\Big ] dz\\&\quad \le C_{r-\frac{1}{2}} \Vert \mathcal {V}_1\Vert _{r,1,\widetilde{\tau }} (\Vert \delta \mathcal {V}\Vert _{r-\frac{1}{2},0,\widetilde{\tau }}^2+ \Vert A^{r} e^{\widetilde{\tau } A}\delta \mathcal {V}\Vert ^2 ), \end{aligned}\\&\begin{aligned}&\Big | \Big \langle A^{r-\frac{1}{2}} e^{\widetilde{\tau } A} (\mathcal {V}_2\cdot \nabla \delta \mathcal {V}), A^{r-\frac{1}{2}} e^{\widetilde{\tau } A} \delta \mathcal {V}\Big \rangle \Big | \\&\quad \le \int _0^1 C_{r-\frac{1}{2}}\Big [ (\Vert A^{r-\frac{1}{2}} e^{\widetilde{\tau } A} \mathcal {V}_2(z)\Vert _{L^2(\mathbb {T}^2)} + \Vert \mathcal {V}_2(z)\Vert _{L^2(\mathbb {T}^2)} ) \Vert A^{r} e^{\widetilde{\tau } A} \delta \mathcal {V}(z)\Vert _{L^2(\mathbb {T}^2)} \Vert A^{r} e^{\widetilde{\tau } A} \delta \mathcal {V}(z)\Vert _{L^2(\mathbb {T}^2)} \\&\qquad + \Vert A^{r} e^{\widetilde{\tau } A} \mathcal {V}_2(z) \Vert _{L^2(\mathbb {T}^2)} \Vert A^{r} e^{\widetilde{\tau } A} \delta \mathcal {V}(z)\Vert _{L^2(\mathbb {T}^2)} \Vert A^{r-\frac{1}{2}} e^{\widetilde{\tau } A} \delta \mathcal {V}(z)\Vert _{L^2(\mathbb {T}^2)}\Big ] dz\\&\quad \le C_{r-\frac{1}{2}} \Vert \mathcal {V}_2\Vert _{r,1,\widetilde{\tau }} \Vert A^{r} e^{\widetilde{\tau } A}\delta \mathcal {V}\Vert ^2, \end{aligned}\\&\Big | \Big \langle A^{r-\frac{1}{2}} e^{\widetilde{\tau } A} \Big [ \Big (\int _0^z \nabla \cdot \delta \mathcal {V}(\varvec{x},s)ds \Big ) \partial _z \mathcal {V}_1 \Big ], A^{r-\frac{1}{2}} e^{\widetilde{\tau } A} \delta \mathcal {V} \Big \rangle \Big | \le C_{r-\frac{1}{2}} \Vert \mathcal {V}_1\Vert _{r,1,\widetilde{\tau }} \Vert A^{r} e^{\widetilde{\tau } A}\delta \mathcal {V}\Vert ^2, \end{aligned}$$

and

3.18 $$\begin{aligned} \begin{aligned}&\Big | \Big \langle A^{r-\frac{1}{2}} e^{\widetilde{\tau } A} \Big [ \Big (\int _0^z \nabla \cdot \mathcal {V}_2(\varvec{x},s)ds \Big ) \partial _z \delta \mathcal {V} \Big ], A^{r-\frac{1}{2}} e^{\widetilde{\tau } A} \delta \mathcal {V} \Big \rangle \Big | \\&\quad \le C_{r-\frac{1}{2}} \Vert A^{r} e^{\widetilde{\tau } A}\mathcal {V}_2\Vert \Vert \partial _z\delta \mathcal {V}\Vert _{r-\frac{1}{2},0,\widetilde{\tau }} \Vert A^{r} e^{\widetilde{\tau } A}\delta \mathcal {V}\Vert \le \frac{\nu }{2} \Vert \partial _z\delta \mathcal {V}\Vert _{r-\frac{1}{2},0,\widetilde{\tau }}^2 + C_{\nu ,r-\frac{1}{2}} \Vert \mathcal {V}_2\Vert _{r,0,\widetilde{\tau }}^2 \Vert A^{r} e^{\widetilde{\tau } A}\delta \mathcal {V}\Vert ^2. \end{aligned}\nonumber \\ \end{aligned}$$

Consequently, combining the calculations between (3.17) and (3.18) yields

$$\begin{aligned} \begin{aligned}&\frac{1}{2}\frac{d}{dt} \Vert \delta \mathcal {V}\Vert _{r-\frac{1}{2},0,\widetilde{\tau }}^2 + \frac{1}{2} \nu \Vert \partial _z \delta \mathcal {V}\Vert _{r-\frac{1}{2},0,\widetilde{\tau }}^2 \\ \le&\Big (\dot{\widetilde{\tau }} + C_{\nu ,r-\frac{1}{2}} \Vert \mathcal {V}_2\Vert _{r,0,\widetilde{\tau }}^2 + C_{r-\frac{1}{2}}(\Vert \mathcal {V}_1\Vert _{r,1,\widetilde{\tau }} + \Vert \mathcal {V}_2\Vert _{r,1,\widetilde{\tau }}) \Big ) \Vert A^{r} e^{\widetilde{\tau } A} \delta \mathcal {V}\Vert ^2 + C_{r-\frac{1}{2}} \Vert \mathcal {V}_1\Vert _{r,1,\widetilde{\tau }} \Vert \delta \mathcal {V}\Vert _{r-\frac{1}{2},0,\widetilde{\tau }}^2 . \end{aligned} \end{aligned}$$

In addition, from (3.13), and (3.14), and the fact that $$\tau _i(t) \ge \widetilde{\tau }(t)$$, $$ i=1,2$$, one can derive that

$$\begin{aligned} \begin{aligned}&\dot{\widetilde{\tau }} + C_{\nu ,r-\frac{1}{2}} \Vert \mathcal {V}_2\Vert _{r,0,\widetilde{\tau }}^2 + C_{r-\frac{1}{2}}(\Vert \mathcal {V}_1\Vert _{r,1,\widetilde{\tau }} + \Vert \mathcal {V}_2\Vert _{r,1,\widetilde{\tau }}) \\ =&- 1 - C_r \sum \limits _{i=1}^2 \bigl (\Vert \mathcal {V}_i(t)\Vert _{r,0,\tau _i(t)}+ \Vert \mathcal {V}_i(t)\Vert ^2_{r,0,\tau _i(t)} + \Vert \partial _z \mathcal {V}_i(t)\Vert _{r,0,\tau _i(t)} \bigr ) \\&\qquad \qquad + C_{\nu ,r-\frac{1}{2}} \Vert \mathcal {V}_2\Vert _{r,0,\widetilde{\tau }}^2 + C_{r-\frac{1}{2}}(\Vert \mathcal {V}_1\Vert _{r,1,\widetilde{\tau }} + \Vert \mathcal {V}_2\Vert _{r,1,\widetilde{\tau }})\\ \le&\big (\widetilde{C}_{\nu ,r-\frac{1}{2}}-C_r \big ) \sum \limits _{i=1}^2 \bigl (\Vert \mathcal {V}_i(t)\Vert _{r,0,\widetilde{\tau }(t)}+ \Vert \mathcal {V}_i(t)\Vert ^2_{r,0,\widetilde{\tau }(t)} + \Vert \partial _z \mathcal {V}_i(t)\Vert _{r,0,\widetilde{\tau }(t)} \bigr ) \le 0, \end{aligned} \end{aligned}$$

where we have chosen

3.19 $$\begin{aligned} C_r := \max \lbrace \widetilde{C}_{\nu ,r-\frac{1}{2}}, C_{r,1}, C_{r,2} \rbrace . \end{aligned}$$

In conclusion, with $C_r$ satisfying (3.19), one has

3.20 $$\begin{aligned} \begin{aligned} \frac{1}{2}\frac{d}{dt} \Vert \delta \mathcal {V}\Vert _{r-\frac{1}{2},0,\widetilde{\tau }}^2 + \frac{1}{2} \nu \Vert \partial _z\delta \mathcal {V}\Vert _{r-\frac{1}{2},0,\widetilde{\tau }}^2 \le C_{r-\frac{1}{2}} \Vert \mathcal {V}_1\Vert _{r,1,\widetilde{\tau }} \Vert \delta \mathcal {V}\Vert _{r-\frac{1}{2},0,\widetilde{\tau }}^2 . \end{aligned} \end{aligned}$$

Applying the Grönwall inequality to (3.20) results in

3.21 $$\begin{aligned} \Vert \delta \mathcal {V}(t)\Vert _{r-\frac{1}{2},0,\widetilde{\tau }(t)}^2 \le \Vert \delta \mathcal {V}(0)\Vert _{r-\frac{1}{2},0,\tau _0}^2 \exp (\int _0^t 2C_{r-\frac{1}{2}} \Vert \mathcal {V}_1(s)\Vert _{r,1,\widetilde{\tau }(s)}ds) \end{aligned}$$

for $t\in [0,\widetilde{\mathcal {T}}]$, which establishes the continuous dependence on the initial data as well as the uniqueness of the weak solutions. This, together with section 3.1, finishes the proof of Theorem 3.1.

### Instantaneous Analyticity in the *z*-Variable

In this section, we will show that the weak solution obtained in Theorem 3.1 immediately becomes analytic in the *z*-variable (and thus analytic in all variables) when $t>0$. Moreover, the radius of analyticity in the *z*-variable increases as long as the solution exists. For simplicity, we consider the even extension for ${\mathcal {V}}$ in the *z*-variable, which is compatible with (1.3), and work in the unit three-dimensional torus $\mathbb {T}^3$ instead of ${\mathcal {D}}$. With abuse of notations, we use ${\mathcal {V}}$ to represent both ${\mathcal {V}}$ in ${\mathcal {D}}$ and its even extension with respect to the *z*-variable in ${\mathbb {T}}^3$.

We first introduce the following notations that are only used in this subsection. For $f\in L^2(\mathbb {T}^3)$ even with respect to the *z*-variable, we consider the following functional space

$$\begin{aligned} \mathcal {S}_{r,s,\tau ,\eta } := \Big \{ f\in L^2(\mathbb {T}^3), \Vert f\Vert _{r,s,\tau ,\eta } < \infty , ~ f ~ \text {even with respect to the { z}-variable} \Big \}, \end{aligned}$$

where

$$\begin{aligned} \Vert f\Vert _{r,s,\tau ,\eta }^2 := \sum \limits _{\varvec{k}\in 2\pi \mathbb {Z}^2, k_3 \in 2\pi {\mathbb {Z}}} \Big (1 + (|\varvec{k}|^{2r} + |k_3|^{2s}) e^{2\tau |\varvec{k}|} e^{2\eta |k_3|}\Big ) |\hat{f}_{\varvec{k},k_3}|^2 \\ \text {and} \qquad \hat{f}_{\varvec{k},k_3} :=\int _{{\mathbb {T}}^3} e^{-i\varvec{k} \cdot \varvec{x}- ik_3 z} f(\varvec{x},z)\, d\varvec{x} dz. \end{aligned}$$

Denote by

$$\begin{aligned} A_h := \sqrt{-\Delta _h}, \quad A_z :=\sqrt{-\partial _{zz}}, \end{aligned}$$

subject to periodic boundary condition, defined by, in terms of the Fourier coefficients,

$$\begin{aligned} (\widehat{A_h^r f})_{\varvec{k},k_3} := |\varvec{k}|^r \hat{f}_{\varvec{k},k_3}, \qquad (\widehat{A_z^sf})_{\varvec{k},k_3} := |k_3|^s \hat{f}_{\varvec{k},k_3}, \qquad ({\varvec{k}} ,k_3) \in 2\pi ({\mathbb {Z}}^2 \times {\mathbb {Z}}), ~ r,s\ge 0. \end{aligned}$$

Accordingly, one has

$$\begin{aligned} \Vert f\Vert _{r,s,\tau ,\eta }^2 = \Vert f\Vert ^2 + \Vert A_h^r e^{\tau A_h} e^{\eta A_z} f\Vert ^2 + \Vert A_z^s e^{\tau A_h} e^{\eta A_z} f\Vert ^2. \end{aligned}$$

With such notations, we establish the following theorem:

#### Theorem 3.2

Assume $\mathcal {V}_0\in \mathcal {S}_{r,0,\tau _0,0}$ with $r>2$ and $\tau _0>0$. Let $\Omega \in \mathbb {R}$ be arbitrary and fixed. Then there exist ${\mathcal {T}}>0$ defined in (3.24), $\tau (t)>0$ given in (3.23), below, and $\eta (t) = \frac{\nu }{2}t$, such that there exists a unique solution ${\mathcal {V}}$ to system (1.1) with (1.2) and (1.3) in $[0,\mathcal {T}]$ satisfying

$$\begin{aligned} \mathcal {V}\in L^\infty \big (0,\mathcal {T}; \mathcal {S}_{r,0,\tau (t),\eta (t)}\big ) \cap L^2\big (0,\mathcal {T}; \mathcal {S}_{r,1,\tau (t),\eta (t)}\big ), \end{aligned}$$

and depending continuously on the initial data. In particular, ${\mathcal {V}}$ immediately becomes analytic in all spatial variables for $t>0$.

#### Remark 4

After restricting ${\mathcal {V}}_0$ and ${\mathcal {V}}$ in ${\mathbb {T}}^2 \times (0,1)$, the solutions in Theorem 3.2 are the same to the ones in Theorem 3.1, thanks to the uniqueness of solutions. Therefore, the gain of analyticity in the *z*-variable of Theorem 3.2 can be regarded as a property to solutions in Theorem 3.1.

#### Remark 5

Theorem 3.2 states the gain of analyticity in the *z*-variable for solutions to system (1.1). One can then apply the result from [23] to study the effect of rotation on the lifespan of solutions after a initial time layer. However, in order to achieve a longer lifespan, the result from [23] requests smallness of Sobolev norms in the baroclinic mode. In this paper, thanks to the effect of viscosity, we are able to relax the condition by requiring smallness in a larger functional space. See Theorem 5.1 and remark 9, below, for more details.

#### Proof

(Sketch of proof) Here we only show the *a priori* estimates. Direct calculation of

$$\begin{aligned} \langle (1.1a), {\mathcal {V}} \rangle + \langle A_h^r e^{\tau A_h}e^{\eta A_z} (1.1a),A_h^r e^{\tau A_h}e^{\eta A_z} {\mathcal {V}}\rangle + \langle e^{\tau A_h}e^{\eta A_z} (1.1a),e^{\tau A_h} e^{\eta A_z} {\mathcal {V}}\rangle , \end{aligned}$$

after applying integration by parts, (1.1c), and (1.3), shows that

$$\begin{aligned} \begin{aligned}&\frac{1}{2} \frac{d}{dt} \Vert {\mathcal {V}}\Vert _{r,0,\tau ,\eta }^2 + \nu \Vert \partial _z {\mathcal {V}}\Vert _{r,0,\tau ,\eta }^2 - \dot{\tau }\Big ( \Vert A_h^{r+\frac{1}{2}} e^{\tau A_h} e^{\eta A_z} {\mathcal {V}}\Vert ^2 + \Vert A_h^{\frac{1}{2}} e^{\tau A_h} e^{\eta A_z} {\mathcal {V}} \Vert ^2\Big )\\&\quad - \dot{\eta } \Big (\Vert A_z^{\frac{1}{2}} A_h^r e^{\tau A_h} e^{\eta A_z} {\mathcal {V}}\Vert ^2 + \Vert A_z^{\frac{1}{2}} e^{\tau A_h} e^{\eta A_z} {\mathcal {V}}\Vert ^2 \Big )\\&\quad + \Big \langle A_h^r e^{\tau A_h} e^{\eta A_z}({\mathcal {V}} \cdot \nabla {\mathcal {V}}), A_h^r e^{\tau A_h} e^{\eta A_z} {\mathcal {V}} \Big \rangle + \Big \langle e^{\tau A_h} e^{\eta A_z}({\mathcal {V}} \cdot \nabla {\mathcal {V}}), e^{\tau A_h} e^{\eta A_z} {\mathcal {V}} \Big \rangle \\&\quad + \Big \langle A_h^r e^{\tau A_h} e^{\eta A_z} \Big ((\int _0^z \nabla \cdot {\mathcal {V}} ds) \partial _z {\mathcal {V}}\Big ), A_h^r e^{\tau A_h} e^{\eta A_z}{\mathcal {V}} \Big \rangle \\&\quad + \Big \langle e^{\tau A_h} e^{\eta A_z} \Big ((\int _0^z \nabla \cdot {\mathcal {V}} ds) \partial _z {\mathcal {V}}\Big ), e^{\tau A_h} e^{\eta A_z}{\mathcal {V}} \Big \rangle = 0. \end{aligned} \end{aligned}$$

Denote by

$$\begin{aligned} E:= & {} \Vert {\mathcal {V}}\Vert _{r,0,\tau ,\eta }^2 = \sum \limits _{(\varvec{k},k_3)\in 2\pi \mathbb {Z}^3} \Big (1 + (|\varvec{k}|^{2r} +1) e^{2\tau |\varvec{k}|} e^{2\eta |k_3|} \Big ) |\hat{{\mathcal {V}}}_{\varvec{k},k_3}|^2, \\ F:= & {} \Vert \partial _z {\mathcal {V}}\Vert _{r,0,\tau ,\eta }^2 = \sum \limits _{(\varvec{k},k_3)\in 2\pi \mathbb {Z}^3} |k_3|^2 \Big (1 + (|\varvec{k}|^{2r} +1) e^{2\tau |\varvec{k}|} e^{2\eta |k_3|} \Big ) |\hat{{\mathcal {V}}}_{\varvec{k},k_3}|^2, \\ G:= & {} \Vert A_h^{r+\frac{1}{2}} e^{\tau A_h} e^{\eta A_z} {\mathcal {V}}\Vert ^2 + \Vert A_h^{\frac{1}{2}} e^{\tau A_h} e^{\eta A_z} {\mathcal {V}} \Vert ^2= \sum \limits _{(\varvec{k},k_3)\in 2\pi \mathbb {Z}^3} (|\varvec{k}|^{2r+1} + |\varvec{k}|^{\frac{1}{2}}) e^{2\tau |\varvec{k}|} e^{2\eta |k_3|} |\hat{{\mathcal {V}}}_{\varvec{k},k_3}|^2, \\ H:= & {} \Vert A_z^{\frac{1}{2}} A_h^r e^{\tau A_h} e^{\eta A_z} {\mathcal {V}}\Vert ^2 + \Vert A_z^{\frac{1}{2}} e^{\tau A_h} e^{\eta A_z} {\mathcal {V}}\Vert ^2 = \sum \limits _{(\varvec{k},k_3)\in 2\pi \mathbb {Z}^3} (|k_3| |\varvec{k}|^{2r} + |k_3|) e^{2\tau |\varvec{k}|} e^{2\eta |k_3|} |\hat{{\mathcal {V}}}_{\varvec{k},k_3}|^2. \end{aligned}$$

Observe that $H\le F$. After setting $\dot{\eta } = \frac{\nu }{2}$, one obtains that

$$\begin{aligned} \begin{aligned}&\frac{1}{2} \frac{d}{dt} E + \frac{1}{2}\nu G - \dot{\tau } G \\&\quad + \Big \langle A_h^r e^{\tau A_h} e^{\eta A_z}({\mathcal {V}} \cdot \nabla {\mathcal {V}}), A_h^r e^{\tau A_h} e^{\eta A_z} {\mathcal {V}} \Big \rangle + \Big \langle e^{\tau A_h} e^{\eta A_z}({\mathcal {V}} \cdot \nabla {\mathcal {V}}), e^{\tau A_h} e^{\eta A_z} {\mathcal {V}} \Big \rangle \\&\quad + \Big \langle A_h^r e^{\tau A_h} e^{\eta A_z} \Big ((\int _0^z \nabla \cdot {\mathcal {V}} ds) \partial _z {\mathcal {V}}\Big ), A_h^r e^{\tau A_h} e^{\eta A_z}{\mathcal {V}} \Big \rangle \\&\quad + \Big \langle e^{\tau A_h} e^{\eta A_z} \Big ((\int _0^z \nabla \cdot {\mathcal {V}} ds) \partial _z {\mathcal {V}}\Big ), e^{\tau A_h} e^{\eta A_z}{\mathcal {V}} \Big \rangle \le 0. \end{aligned} \end{aligned}$$

For the nonlinear terms, by applying similar calculations as in Lemma A.1 and Lemma A.2 (we also refer the readers to [23] for detailed calculations in $\mathbb {T}^3$), one can obtain that

$$\begin{aligned} \begin{aligned} \Big | \Big \langle A_h^r e^{\tau A_h} e^{\eta A_z}({\mathcal {V}} \cdot \nabla {\mathcal {V}}), A_h^r e^{\tau A_h} e^{\eta A_z} {\mathcal {V}} \Big \rangle \Big | + \Big | \Big \langle e^{\tau A_h} e^{\eta A_z}({\mathcal {V}} \cdot \nabla {\mathcal {V}}), e^{\tau A_h} e^{\eta A_z} {\mathcal {V}} \Big \rangle \Big | \le C_r \Big (E^{\frac{1}{2}}+F^{\frac{1}{2}} \Big )G, \end{aligned} \\ \begin{aligned} \Big |\Big \langle A_h^r e^{\tau A_h} e^{\eta A_z} \Big ((\int _0^z \nabla \cdot {\mathcal {V}} ds) \partial _z {\mathcal {V}}\Big ), A_h^r e^{\tau A_h} e^{\eta A_z}{\mathcal {V}} \Big \rangle \Big | \le C_r F^{\frac{1}{2}} G, \end{aligned} \end{aligned}$$

and thanks to the Young inequality,

$$\begin{aligned} \Big | \Big \langle e^{\tau A_h} e^{\eta A_z} \Big ((\int _0^z \nabla \cdot {\mathcal {V}} ds) \partial _z {\mathcal {V}}\Big ), e^{\tau A_h} e^{\eta A_z}{\mathcal {V}} \Big \rangle \Big | \le C_r F^{\frac{1}{2}}E^{\frac{1}{2}}G^{\frac{1}{2}} \le \frac{C_r}{\nu }E G + \frac{\nu }{4}F. \end{aligned}$$

Therefore, combining all the estimates above leads to

3.22 $$\begin{aligned} \begin{aligned}&\frac{d}{dt} E + \frac{1}{2}\nu F \le \Big (\dot{\tau }+ C_{r} (E^{\frac{1}{2}} + F^{\frac{1}{2}} + \frac{1}{\nu }E) \Big )G . \end{aligned} \end{aligned}$$

By taking $\dot{\tau }+ C_{r}(E^{\frac{1}{2}} + F^{\frac{1}{2}} + \frac{1}{\nu }E)=0$, one obtains

$$\begin{aligned} E(t) + \frac{1}{2}\nu \int _0^t F(s) ds \le E(0). \end{aligned}$$

Integrating in time for $\dot{\tau }+ C_{r}(E^{\frac{1}{2}} + F^{\frac{1}{2}} + \frac{1}{\nu }E)=0$, we have

3.23 $$\begin{aligned} \tau (t) = \tau _0 - \int _0^t C_r (E^{\frac{1}{2}}(s) + F^{\frac{1}{2}}(s) + \frac{1}{\nu }E(s)) ds \ge \tau _0 - C_r \Big ( E^{\frac{1}{2}}(0) (t + \sqrt{\frac{2 t}{\nu }} ) + E(0)\frac{t}{\nu } \Big ). \end{aligned}$$

Since $E(0) = \Vert {\mathcal {V}}\Vert _{r,0,\tau ,0}^2$, we denote by

3.24 $$\begin{aligned} {\mathcal {T}} := \Big ( \frac{\sqrt{\frac{2}{\nu }\Vert {\mathcal {V}} \Vert _{r,0,\tau ,0}^2 + \frac{2\tau _0}{C_r}(\frac{\Vert {\mathcal {V}} \Vert _{r,0,\tau ,0}^2}{\nu } + \Vert {\mathcal {V}}\Vert _{r,0,\tau ,0})} - \sqrt{\frac{2}{\nu }} \Vert {\mathcal {V}}\Vert _{r,0,\tau ,0}}{2(\frac{\Vert {\mathcal {V}} \Vert _{r,0,\tau ,0}^2}{\nu } + \Vert {\mathcal {V}}\Vert _{r,0,\tau ,0})} \Big )^{\frac{1}{2}}>0, \end{aligned}$$

which solves

$$\begin{aligned} \Vert {\mathcal {V}}\Vert _{r,0,\tau ,0}({\mathcal {T}} + \sqrt{{\mathcal {T}}}) + \frac{1}{\nu } \Vert {\mathcal {V}}\Vert _{r,0,\tau ,0}^2 {\mathcal {T}} = \frac{\tau _0}{2C_r}. \end{aligned}$$

Then one has

$$\begin{aligned} \tau (t) \ge \tau _0/2 > 0 \quad \text {for} \quad t \in [0,{\mathcal {T}}]. \end{aligned}$$

Notice that the radius of analyticity in the *z* variable satisfies $\eta = \frac{\nu }{2}t$. Therefore, (3.22) implies that

$$\begin{aligned} \mathcal {V}\in L^\infty \big (0,\mathcal {T}; \mathcal {S}_{r,0,\tau (t), \eta (t)}\big ) \cap L^2\big (0,\mathcal {T}; \mathcal {S}_{r,1,\tau (t),\eta (t)}\big ). \end{aligned}$$

Based on the estimates above, one is able to show the existence, uniqueness, and continuous dependence on the initial data of the solution ${\mathcal {V}}$. We omit the details. $\square$

## The Limit Resonant System

In this section, we derive the formal limit resonant system, i.e., the limit system of system (1.1) (or, equivalently, system (2.17)) as $|\Omega |\rightarrow \infty$, and discuss some properties of the limit resonant system. Recall that from (2.22), we have

4.1 $$\begin{aligned} \begin{aligned} \partial _t \mathcal {V}_+ =&-e^{i\Omega t} \Big (\underbrace{\mathcal {V}_+ \cdot \nabla \mathcal {V}_+ - {\mathfrak {P}}_0( \mathcal {V}_+ \cdot \nabla \mathcal {V}_+ + (\nabla \cdot \mathcal {V}_+) \mathcal {V}_+) - (\int _0^z \nabla \cdot \mathcal {V}_+(\varvec{x},s)ds ) \partial _z \mathcal {V}_+}_{=:I_1} \Big ) \\&- \Big [\underbrace{\Big (\overline{\mathcal {V}} \cdot \nabla \mathcal {V}_+ + \frac{1}{2}(\mathcal {V}_+ \cdot \nabla )(\overline{\mathcal {V}}+i\overline{\mathcal {V}}^\perp ) \Big ) - \nu \partial _{zz} \mathcal {V}_+}_{=:I_0}\Big ] \\&- e^{-i\Omega t} \Big (\underbrace{\mathcal {V}_- \cdot \nabla \mathcal {V}_+ - {\mathfrak {P}}_0( \mathcal {V}_- \cdot \nabla \mathcal {V}_+ + (\nabla \cdot \mathcal {V}_-) \mathcal {V}_+) - (\int _0^z \nabla \cdot \mathcal {V}_-(\varvec{x},s)ds ) \partial _z \mathcal {V}_+}_{=:I_{-1}} \Big ) \\&- e^{-2i\Omega t} \underbrace{\frac{1}{2} (\mathcal {V}_- \cdot \nabla )(\overline{\mathcal {V}}+i\overline{\mathcal {V}}^\perp )}_{=:I_{-2}}. \end{aligned} \end{aligned}$$

We can further rewrite (4.1) as

$$\begin{aligned}&\partial _t \Big [\mathcal {V}_+ - \frac{i}{\Omega } \Big (e^{i\Omega t}I_1 - e^{-i\Omega t} I_{-1} - \frac{1}{2} e^{-2i\Omega t} I_{-2} \Big ) \Big ] = - \frac{i}{\Omega } \Big (e^{i\Omega t}\partial _t I_1 - e^{-i\Omega t}\partial _t I_{-1} - \frac{1}{2}e^{-2i\Omega t}\partial _t I_{-2}\Big ) - I_0. \end{aligned}$$

Denote by the formal limits of $\mathcal {V}_+, \mathcal {V}_-$, and $\overline{\mathcal {V}}$ to be $V_+, V_-$, and $\overline{V}$, respectively. By taking limit $\Omega \rightarrow \infty$, we obtain the limit resonant equation for $\mathcal {V}_+$ is

4.2 $$\begin{aligned} \partial _t V_+ = - (\overline{V} \cdot \nabla )V_+ - \frac{1}{2} (V_+ \cdot \nabla )(\overline{V}+i\overline{V}^\perp ) + \nu \partial _{zz} V_+ . \end{aligned}$$

Similarly, one has

4.3 $$\begin{aligned} \partial _t V_- = - (\overline{V} \cdot \nabla )V_- - \frac{1}{2} (V_- \cdot \nabla )(\overline{V}-i\overline{V}^\perp ) + \nu \partial _{zz} V_- , \end{aligned}$$

and

4.4 $$\begin{aligned} \partial _t \overline{V} + \overline{V}\cdot \nabla \overline{V} + \nabla p =0, \qquad \nabla \cdot \overline{V} = 0, \qquad \partial _z p = 0. \end{aligned}$$

Notice that (4.4) is nothing but the 2D Euler equations. Accordingly, we consider the initial conditions

4.5 $$\begin{aligned} (\overline{V}_0, (V_+)_0, (V_-)_0) = (\overline{\mathcal {V}}_0, \frac{1}{2}(\widetilde{\mathcal {V}}_0 + i\widetilde{\mathcal {V}}_0^\perp ), \frac{1}{2}(\widetilde{\mathcal {V}}_0 - i\widetilde{\mathcal {V}}_0^\perp ) ) \end{aligned}$$

for equations (4.2)–(4.4). Since $\overline{V}_0$, $\overline{{\mathcal {V}}}_0$, and $\widetilde{{\mathcal {V}}}_0$ are real valued, one has that $(V_+)_0 = (V_-)_0^*$, $(V_+)_0+ (V_-)_0 = i ((V_+)_0 - (V_-)_0)^\perp = \widetilde{{\mathcal {V}}}_0$, and, thanks to (4.4), $\overline{V}$ is real valued. Thanks to (4.2) and (4.3), one has

4.6 $$\begin{aligned}&\partial _t ( V_+ - V_-^* ) = - ( \overline{V} \cdot \nabla ) ( V_+ - V_-^* ) - \dfrac{1}{2} \bigl [( V_+ - V_-^* ) \cdot \nabla \bigr ] ( {\overline{V}} + i {\overline{V}}^\perp ) + \nu \partial _{zz} ( V_+ - V_-^* ), \end{aligned}$$

4.7 $$\begin{aligned}&\partial _t \bigl [(V_+ + V_-) - i ( V_+ - V_-)^\perp \bigr ] = - ( {\overline{V}} \cdot \nabla ) \bigl [(V_+ + V_-) - i ( V_+ - V_-)^\perp \bigr ] - \dfrac{1}{2} \nonumber \\&+ \nu \partial _{zz} \bigl [(V_+ + V_-) - i ( V_+ - V_-)^\perp \bigr ]. \end{aligned}$$

Therefore, provided solutions exist and are well-posed, one has $V_+ \equiv V_-^*$ and $V_+ + V_- \equiv i ( V_+ - V_-)^\perp$. Let

4.8 $$\begin{aligned} {\widetilde{V}} := V_+ + V_- . \end{aligned}$$

Notice that, according to (2.19), ${\widetilde{V}}$ is the formal limit of ${\mathcal {V}}_+ + {\mathcal {V}}_- = e^{-i\Omega t} {\mathfrak {P}}_+ {\mathcal {V}} + e^{i\Omega t} {\mathfrak {P}}_- {\mathcal {V}}$, as $\Omega \rightarrow \infty$. It is easy to verify that

4.9 $$\begin{aligned} V_\pm = \dfrac{1}{2} ( {\widetilde{V}} \pm i {\widetilde{V}}^\perp ), \end{aligned}$$

and

$$\begin{aligned} \partial _t \widetilde{V} + (\overline{V}\cdot \nabla )\widetilde{V} + \frac{1}{2}(\widetilde{V}\cdot \nabla \overline{V} - \widetilde{V}^\perp \cdot \nabla \overline{V}^\perp ) - \nu \partial _{zz} \widetilde{V} =0, \end{aligned}$$

or, thanks to $\nabla \cdot \overline{V} = 0$, equivalently,

4.10 $$\begin{aligned} \partial _t \widetilde{V} + \overline{V} \cdot \nabla \widetilde{V} + \frac{1}{2} \widetilde{V}^\perp (\nabla ^\perp \cdot \overline{V}) - \nu \partial _{zz} \widetilde{V} = 0. \end{aligned}$$

In summary, to solve the limit equations (4.2)–(4.4) with (4.5) is equivalent to solve the following equations:

4.11a $$\begin{aligned}&\partial _t \overline{V} + \overline{V}\cdot \nabla \overline{V} + \nabla p = 0, \end{aligned}$$

4.11b $$\begin{aligned}&\nabla \cdot \overline{V} = 0, \qquad \partial _z p = 0 , \end{aligned}$$

4.11c $$\begin{aligned}&\partial _t \widetilde{V} + \overline{V} \cdot \nabla \widetilde{V} + \frac{1}{2} \widetilde{V}^\perp (\nabla ^\perp \cdot \overline{V}) - \nu \partial _{zz} \widetilde{V} = 0 , \end{aligned}$$

4.11d $$\begin{aligned}&\partial _z\widetilde{V}\big |_{z=0,1} = 0, \qquad \overline{V}(0) = \overline{\mathcal {V}}_0, \quad \text {and} \quad \widetilde{V}(0) \nonumber \\&= \widetilde{{\mathcal {V}}}_0. \end{aligned}$$

Notice that, thanks to our choice of $\overline{ {\mathcal {V}}}_0$ and $\widetilde{{\mathcal {V}}}_0$, one has ${\mathfrak {P}}_0 \overline{V} = \overline{V}$ and ${\mathfrak {P}}_0 \widetilde{V} = 0$. In addition, (4.11a)–(4.11b) is the 2*D* Euler system, and (4.11c) is a linear transport equation with a stretching term and vertical dissipation. In the rest of this section, we summarize the well-posedness theory of (4.11).

### Well-posedness Theory of (4.11a) and (4.11b)

The global well-posedness of solutions to the 2*D* Euler system (4.11a)–(4.11b) in Sobolev spaces $H^r(\mathbb {T}^2) = \mathcal {S}_{r,0,0}$ with $r > 3$ is a classical result (see, e.g., [7]). Moreover, from equation (3.84) in [7], for $r > 3$, we have

4.12 $$\begin{aligned} \frac{d}{dt}\Vert \overline{V}\Vert _{r,0,0} \le C_r \Vert \overline{V}\Vert _{r,0,0}(1+\ln ^+\Vert \overline{V}\Vert _{r,0,0}). \end{aligned}$$

Let $\Vert \overline{V}_0\Vert _{r,0,0} \le M$ for some $M\ge 0$. Denote by $W(t):=\Vert \overline{V}(t)\Vert _{r,0,0} + e$. Thanks to $\ln ^+ x + 1 \le 2\ln (x+e)$, from (4.12), we have

$$\begin{aligned} \frac{d}{dt} W \le C_r W \ln W. \end{aligned}$$

Therefore, one can obtain that

4.13 $$\begin{aligned} \Vert \overline{V}(t)\Vert _{r,0,0} \le W(t) \le W(0)^{e^{C_r t}} = (\Vert \overline{V}_0\Vert _{r,0,0} +e)^{e^{C_r t}} \le (M+e)^{e^{C_r t}} =: \theta _{M,r}(t). \end{aligned}$$

The authors in [40] proved the global existence of solutions to system (4.11a)–(4.11b) for initial data in the space of analytic functions. For completion, we state it here, with slight modifications to meet our settings. See also [23].

#### Proposition 4.1

Assume $\overline{V}_0 \in \mathcal {S}\cap \mathcal {S}_{r,0,\tau _0}$ with $r>3$ and $\tau _0 >0$, and suppose that $\Vert \overline{V}_0\Vert _{r,0,\tau _0} \le M$ for some $M\ge 0$. Let

$$\begin{aligned} \tau (t) := \tau _0 \exp \Big ( -C_r \int _0^t h(s)ds\Big ), \end{aligned}$$

where

$$\begin{aligned} h^2(t) := \Vert \overline{V}_0\Vert _{r,0,\tau _0}^2 + C_r\int _0^t \theta _{M,r}^3(s)ds, \end{aligned}$$

with $\theta _{M,r}(t)$ defined in (4.13). Then for any given time $\mathcal {T}>0$, there exists a unique solution

$$\begin{aligned} \overline{V}\in L^\infty (0, \mathcal {T}; \mathcal {S}\cap \mathcal {S}_{r,0,\tau (t)} ) \end{aligned}$$

to system (4.11a)–(4.11b). Moreover, there exist constants $C_M>1$ and $C_r>1$ such that

$$\begin{aligned} \Vert \overline{V}(t)\Vert _{r,0,\tau (t)}^2 \le h^2(t) \le C_M^{\exp (C_r t)}. \end{aligned}$$

The solution is continuously depending on the initial data.

### Global Well-posedness of System (4.11)

In this subsection, we establish the global well-posedness of limit resonant system (4.11) in both Sobolev spaces $\mathcal {S}_{r,s,0}$ and analytic-Sobolev spaces $\mathcal {S}_{r,s,\tau }$.

#### Proposition 4.2

Let $r > 2$ and $s \in \lbrace 0,1 \rbrace$. Assume that $\overline{V}_0 \in \mathcal {S}\cap \mathcal {S}_{r+1,0,0}$ and $\widetilde{V}_0 \in \mathcal {S}\cap \mathcal {S}_{r,s,0}$. Let $M\ge 0$ be the constant such that $\Vert \overline{V}_0\Vert _{r+1,0,0} \le M$. Then there exists a function $K(t) := C_M^{\exp (C_r t)}$ with constants $C_M>1$ and $C_r>1$, such that for any given time $\mathcal {T}>0$, there exists a unique solution $( \overline{V}, \widetilde{V} )\in L^\infty (0,\mathcal {T}; \mathcal {S} \cap \mathcal {S}_{r+1,0,0}) \times L^\infty (0,\mathcal {T}; \mathcal {S}\cap \mathcal {S}_{r,s,0})$ of system (4.11), which satisfies

4.14 $$\begin{aligned} \Vert \overline{V}(t)\Vert _{r+1,0,0} \le K(t) \qquad \text {and} \qquad \Vert \widetilde{V}(t)\Vert ^2_{r,s,0} + 2\nu \int _0^t \Vert \partial _z \widetilde{V}(\xi )\Vert ^2_{r,s,0} d\xi \le \Vert \widetilde{V}_0\Vert ^2_{r,s,0} \; e^{\int _0^t K(s) \,ds}. \end{aligned}$$

On the other hand, suppose that $\overline{V}_0 \in \mathcal {S}\cap \mathcal {S}_{r+1,0,\tau _0}$ and $\widetilde{V}_0 \in \mathcal {S}\cap \mathcal {S}_{r,s,\tau _0}$ with $\tau _0 >0$, and that $\Vert \overline{V}_0\Vert _{r+1,0,\tau _0}\le M$. Let

4.15 $$\begin{aligned} \tau (t) := \tau _0 \exp (-\int _0^t K(s)ds). \end{aligned}$$

Then for any given time $\mathcal {T}>0$, there exists a unique solution $(\overline{V},\widetilde{V}) \in L^\infty (0,\mathcal {T}; \mathcal {S} \cap \mathcal {S}_{r+1,0,\tau }) \times L^\infty (0,\mathcal {T}; \mathcal {S} \cap \mathcal {S}_{r,s,\tau })$ of system (4.11) such that

4.16 $$\begin{aligned} \Vert \overline{V}(t)\Vert _{r+1,0,\tau (t)} \le K(t) \quad \text {and} \quad \Vert \widetilde{V}(t)\Vert ^2_{r,s,\tau (t)} + 2\nu \int _0^t \Vert \partial _z \widetilde{V}(\xi )\Vert ^2_{r,s,\tau (\xi )} d\xi \le \Vert \widetilde{V}_0\Vert ^2_{r,s,\tau _0} e^{\int _0^t K(s)\,ds}. \end{aligned}$$

The solutions continuously depend on the initial data.

#### Proof

(Sketch of proof) We will consider the case when $s=1$ and only show the *a priori* estimates. The construction of solutions, uniqueness, and continuous dependency of solutions on initial data, as well as the case when $s=0$, are left to readers as exercises. The global well-posedness of the 2*D* Euler equations in Sobolev spaces and corresponding growth estimate have been reviewed in the previous subsection. From (4.13), we obtain that

4.17 $$\begin{aligned} \Vert \overline{V}\Vert _{r+1,0,0} \le K_1(t) \end{aligned}$$

for some function $K_1(t) := C_{M,1}^{\exp (C_{r,1}t)}$ with some constants $C_{M,1}, C_{r,1} > 1$.

Denote by ${\mathfrak {I}}$ the identity map. For the growth of $\Vert \widetilde{V}\Vert _{H^{r}}$, after calculating $2 \langle (4.11\mathrm{c}), ({\mathfrak {I}} - \partial _{zz}) {\widetilde{V}} \rangle + 2 \langle A^r (4.11\mathrm{c}), ({\mathfrak {I}} - \partial _{zz}) A^r {\widetilde{V}} \rangle$ and applying integration by parts to the resultant, one has, thanks to $\partial _z \overline{V} = 0$, $\nabla \cdot \overline{V}=0$, and $r>\frac{5}{2}$, for some constant $C_{r,s} > 0$,

4.18 $$\begin{aligned} \frac{d}{dt}\Vert \widetilde{V}\Vert ^2_{r,1,0} + 2\nu \Vert \partial _z \widetilde{V}\Vert ^2_{r,1,0} \le C_{r,s}\Vert \overline{V}\Vert _{r+1,0,0} \Vert \widetilde{V}\Vert ^2_{r,1,0}. \end{aligned}$$

After applying the Grönwall inequality to the above, by virtue of (4.17), we obtain

4.19 $$\begin{aligned} \Vert \widetilde{V}(t)\Vert ^2_{r,1,0} + 2\nu \int _0^t \Vert \partial _z \widetilde{V}(\xi )\Vert ^2_{r,1,0} d\xi \le \Vert \widetilde{V}_0\Vert ^2_{r,1,0}\exp \Big (C_{r,s} \int _0^t K_1(\xi )d\xi \Big ). \end{aligned}$$

On the other hand, the global well-posedness of the 2*D* Euler equations in the space of analytic functions and the corresponding growth estimate are summarized in Proposition 4.1. We can first choose some suitable function $K_2(t) := C_{M,2}^{\exp (C_{r,2}t)}$, with $C_{M,2}, C_{r,2} > 1$, such that $\Vert \overline{V}(t)\Vert _{r+1,0,\tau _E(t)} \le K_2(t)$ with $\tau _E(t) := \tau _0 \exp (-\int _0^t K_2(s)ds)$.

Let $\tau = \tau (t)$ to be determined. For $\widetilde{V}$, after calculating $\langle (4.11\mathrm{c}), ({\mathfrak {I}} - \partial _{zz}) {\widetilde{V}} \rangle + \langle A^r e^{\tau A} (4.11\mathrm{c}), ({\mathfrak {I}} - \partial _{zz}) A^r e^{\tau A} {\widetilde{V}} \rangle$ and applying integration by parts, the Hölder inequality, the Sobolev inequality, Lemma 2.1, and Lemma A.4 to the resultant, since $r>2$, one has, for some constant $C_{r,a} >0$,

4.20 $$\begin{aligned} \begin{aligned}&\frac{1}{2} \frac{d}{dt} \Vert \widetilde{V} \Vert _{r,1,\tau }^2 + \nu \Vert \partial _z \widetilde{V} \Vert _{r,1,\tau }^2 - \dot{\tau } \Big ( \Vert A^{r+\frac{1}{2}} e^{\tau A} \widetilde{V} \Vert ^2 + \Vert A^{r+\frac{1}{2}} e^{\tau A} \partial _z \widetilde{V} \Vert ^2 \Big )\\ =&\underbrace{ - \Big \langle \overline{V}\cdot \nabla \widetilde{V}, \widetilde{V} \Big \rangle - \frac{1}{2} \Big \langle (\nabla ^\perp \cdot \overline{V}) \widetilde{V}^\perp , \widetilde{V} \Big \rangle - \Big \langle \overline{V}\cdot \nabla \partial _z \widetilde{V}, \partial _z \widetilde{V} \Big \rangle - \frac{1}{2} \Big \langle (\nabla ^\perp \cdot \overline{V}) \partial _z \widetilde{V}^\perp , \partial _z \widetilde{V} \Big \rangle }_{=0}\\&- \Big \langle A^r e^{\tau A} (\overline{V}\cdot \nabla \widetilde{V}), A^r e^{\tau A} \widetilde{V} \Big \rangle - \frac{1}{2} \Big \langle A^r e^{\tau A} \Big ( (\nabla ^\perp \cdot \overline{V}) \widetilde{V}^\perp \Big ), A^r e^{\tau A} \widetilde{V} \Big \rangle \\&- \Big \langle A^r e^{\tau A} (\overline{V}\cdot \nabla \partial _z \widetilde{V}), A^r e^{\tau A} \partial _z \widetilde{V} \Big \rangle - \frac{1}{2} \Big \langle A^r e^{\tau A} \Big ( (\nabla ^\perp \cdot \overline{V}) \partial _z \widetilde{V}^\perp \Big ), A^r e^{\tau A} \partial _z \widetilde{V} \Big \rangle \\ =&- \Big (\Big \langle A^r e^{\tau A} (\overline{V}\cdot \nabla \widetilde{V}), A^r e^{\tau A} \widetilde{V} \Big \rangle - \underbrace{\Big \langle \overline{V}\cdot \nabla A^r e^{\tau A} \widetilde{V}, A^r e^{\tau A} \widetilde{V} \Big \rangle }_{=0}\Big )\\&- \Big (\Big \langle A^r e^{\tau A} (\overline{V}\cdot \nabla \partial _z \widetilde{V}), A^r e^{\tau A} \partial _z \widetilde{V} \Big \rangle - \underbrace{\Big \langle \overline{V}\cdot \nabla A^r e^{\tau A} \partial _z \widetilde{V}, A^r e^{\tau A} \partial _z \widetilde{V} \Big \rangle }_{=0}\Big )\\&- \frac{1}{2} \Big \langle A^r e^{\tau A} \Big ( (\nabla ^\perp \cdot \overline{V}) \widetilde{V}^\perp \Big ) , A^r e^{\tau A} \widetilde{V} \Big \rangle - \frac{1}{2} \Big \langle A^r e^{\tau A} \Big ( (\nabla ^\perp \cdot \overline{V}) \partial _z \widetilde{V}^\perp \Big ), A^r e^{\tau A}\partial _z \widetilde{V} \Big \rangle \\ \le&C_{r,a}\tau \Vert \overline{V} \Vert _{r+1,0,\tau } \Big ( \Vert A^{r+\frac{1}{2}} e^{\tau A} \widetilde{V} \Vert ^2 + \Vert A^{r+\frac{1}{2}} e^{\tau A} \partial _z \widetilde{V} \Vert ^2 \Big ) + C_{r,a}\Vert \overline{V} \Vert _{r+1,0,\tau } \Vert \widetilde{V} \Vert _{r,1,\tau }^2 . \end{aligned} \end{aligned}$$

Now, let

4.21 $$\begin{aligned} K(t) := \max \lbrace (1 + C_{r,s} ) K_1(t), (1 + C_{r,a}) K_2(t) \rbrace \qquad \text {and} \qquad \tau = \tau (t) := \tau _0 \exp \left( - \int _0^t K(s) \,ds \right) . \nonumber \\ \end{aligned}$$

Then $$ \tau (t) $$ satisfies

$$\begin{aligned} \tau (t) \le \tau _E(t) \qquad \text {and} \qquad \dot{\tau } + C_{r,a} \tau \Vert \overline{V} \Vert _{r+1,0,\tau } \le \dot{\tau } + C_{r,a} \tau \Vert \overline{V} \Vert _{r+1,0,\tau _E} \le {\dot{\tau }} + C_{r,a}\tau K_2 \le 0. \end{aligned}$$

Therefore,

4.22 $$\begin{aligned} \Vert \overline{V} (t)\Vert _{r+1,0,\tau (t)} \le \Vert \overline{V} (t)\Vert _{r+1,0,\tau _E(t)} \le K_2(t) \le K(t), \end{aligned}$$

and, after applying the Grönwall inequality to (4.20), we have

4.23 $$\begin{aligned} \begin{aligned} \Vert \widetilde{V}(t) \Vert _{r,1,\tau (t)}^2 + 2 \nu \int _0^t \Vert \partial _z \widetilde{V}(\xi ) \Vert _{r,1,\tau (\xi )}^2 d \xi \le \Vert \widetilde{V}_0 \Vert _{r,1,\tau _0}^2 \exp \Big (\int _0^t C_{r,a} \Vert \overline{V} (\xi )\Vert _{r+1,0,\tau (\xi )}d\xi \Big )\\ \le \Vert \widetilde{V}_0 \Vert _{r,1,\tau _0}^2 e^{\int _0^t C_{r,a} K_2(s)\,ds} \le \Vert \widetilde{V}_0 \Vert _{r,1,\tau _0}^2 e^{\int _0^t K(s)\,ds}. \end{aligned} \end{aligned}$$

Consequently, according to (4.17), (4.19), (4.22), and (4.23), *K* and $\tau$ as in (4.21) verify (4.14) and (4.16).

$$\square $$

#### Remark 6

From Proposition 4.2, one can see that the growth of $\Vert \overline{V}(t)\Vert _{r+1,0,0}$ and $\Vert \overline{V}(t)\Vert _{r+1,0,\tau (t)}$ are double exponential in time, while the growth of $\Vert \widetilde{V}(t)\Vert _{r,s,0}$ and $\Vert \widetilde{V}(t)\Vert _{r,s,\tau (t)}$ are triple exponential in time.

#### Remark 7

Thanks to (4.9), similarly as in (2.20), we have

$$\begin{aligned} \Vert V_+\Vert _{r,s,\tau }^2 = \Vert V_-\Vert _{r,s,\tau }^2 = \frac{1}{2}\Vert \widetilde{V}\Vert _{r,s,\tau } ^2, \end{aligned}$$

whose growths are also triple exponential.

#### Remark 8

Proposition 4.2 is for the general initial data. However, by considering special solutions to the 2D Euler equations, one has the following:

- Supposed that $\overline{V}$ is uniformly-in-time bounded in $\mathcal {S}_{r+1,0,\tau }$, i.e., $\sup _{0\le t < \infty }\Vert \overline{V}(t)\Vert _{r+1,0,\tau } \le C_{M,r}$ for some positive constant $C_{M,r}$, then one can control the growth of $\Vert \widetilde{V}(t)\Vert _{r,1,\tau }$ by one exponential in time.
- Supposed that $\sup _{0\le t < \infty } \Vert \overline{V}(t)\Vert _{r+1,0,\tau } \le \frac{\nu }{4C_{r,\alpha }}$ is small enough, by applying the Poincaré inequality and with $\tau$ chosen suitably, from (4.20) one can derive that
  $$\begin{aligned} \frac{d}{dt}\Vert \widetilde{V}\Vert ^2_{r,1,\tau } + \frac{1}{2}\nu \Vert \partial _z \widetilde{V}\Vert ^2_{r,1,\tau } \le -\nu \Vert \widetilde{V}\Vert ^2_{r,1,\tau }. \end{aligned}$$
  After applying the Grönwall inequality to the above, we obtain
  $$\begin{aligned} \Vert \widetilde{V}(t)\Vert _{r,1,\tau (t)}^2 e^{\nu t} + \frac{1}{2}\nu \int _0^t \Vert \partial _z \widetilde{V}(\xi )\Vert ^2_{r,1,\tau (\xi )} e^{\nu \xi } d\xi \le \Vert \widetilde{V}_0\Vert _{r,1,\tau _0}^2. \end{aligned}$$
  In particular, the estimate above holds when $\overline{V} \equiv 0$, i.e., zero solutions to the 2D Euler equations.

## Effect of Fast Rotation

In this section, we investigate the effect of rotation on the lifespan $\mathcal {T}$ of solutions to system (1.1). We show that the existing time of the solution in $\mathcal {S}_{r,0,\tau (t)}$ can be prolonged for large $|\Omega |$ provided that the Sobolev norm $\Vert \widetilde{\mathcal {V}}_0\Vert _{\frac{5}{2}+\delta ,1,0}$ is small, while the analytic-Sobolev norm $\Vert \widetilde{\mathcal {V}}_0\Vert _{r,0,\tau _0}$ can be large. Such initial data is referred to as “well-prepared” initial data.

### Theorem 5.1

Let $\delta \in (0,\frac{1}{2})$ be a constant. Let $|\Omega | \ge |\Omega _0| > 1$ and $|\Omega _0|$ be large enough such that condition (5.3) below holds. Assume $\overline{\mathcal {V}}_0 \in \mathcal {S}\cap \mathcal {S}_{r+3,0,\tau _0}$, $\widetilde{\mathcal {V}}_0 \in \mathcal {S}\cap \mathcal {S}_{r+2,0,\tau _0}\cap \mathcal {S}_{r+1,1,\tau _0}$ with $r>2$ and $\tau _0>0$. Let $M\ge 0$ be such that

5.1 $$\begin{aligned} \Vert \overline{{\mathcal {V}}}_0 \Vert _{r+3,0,\tau _0}^2 + \Vert \widetilde{{\mathcal {V}}}_0 \Vert _{r+2,0,\tau _0}^2 + \Vert \widetilde{{\mathcal {V}}}_0 \Vert _{r+1,1,\tau _0}^2 \le M, \end{aligned}$$

and

5.2 $$\begin{aligned} \Vert \widetilde{\mathcal {V}}_0\Vert _{\frac{3}{2}+\delta ,0,0} \le \frac{M}{|\Omega _0|^{1/2}}. \end{aligned}$$

Then there exists a time $\mathcal {T} = \mathcal {T}(\tau _0, |\Omega _0|, M, r, \nu )$ satisfying

5.3 $$\begin{aligned} {\mathcal {T}} = \frac{1}{C_{\tau _0, M, r} } \log \left[ e^{-C_{\tau _0, M, r}}\log \left[ \log \left[ C_{\tau _0, M, r,\nu } \log \left( C_{\tau _0, M, r,\nu } |\Omega _0|\right) \right] \right] \right] > 0, \end{aligned}$$

for some positive constant $C_{\tau _0, M, r,\nu }>0$ depending only on $\tau _0, M$, and *r*, such that the unique solution $\mathcal {V}$ obtained in Theorem 3.1 satisfies

5.4 $$\begin{aligned} \mathcal {V} \in L^\infty (0,\mathcal {T};\mathcal {S} \cap \mathcal {S}_{r,0,\tau (t)}), \end{aligned}$$

with $\tau (t) > 0$, $t \in [0,{\mathcal {T}}]$, satisfying (5.38), below. In particular, from (5.3), $\mathcal {T} \rightarrow \infty$ as $|\Omega _0|^{1/2}\rightarrow \infty$, for any fixed $\nu$.

### Remark 9

Recall that the result in [23] requires the initial baroclinic mode $\widetilde{\mathcal {V}}_0$ to be small in the $H^{3+\delta }$ space instead of (5.2) in Theorem 5.1. This relaxation on the requirement of $\widetilde{\mathcal {V}}_0$ is due to the vertical viscosity.

In Theorem 5.1, we consider general initial data $\overline{{\mathcal {V}}}_0$ for the barotropic mode, where the vertical viscosity helps relax the requirement on the initial baroclinic mode, but does not help prolong the lifespan. By virtue of Remark 8, when the solution $\overline{V}$ to the 2*D* Euler equations with initial condition $\overline{{\mathcal {V}}}_0$ satisfies certain conditions, the smallness condition (5.2) can be relaxed and the result (5.3) can be improved. The following theorem is the summary of these results:

### Theorem 5.2

With the same assumptions as in Theorem 5.1, let $\overline{V}(t)$ be the solution to the 2*D* Euler equations with initial condition $\overline{V}_0 = \overline{{\mathcal {V}}}_0$. Then

(i) if $\Vert \overline{V}(t)\Vert _{r+3,0,\tau (t)} \le C_{M,r}$, the result (5.3) can be improved to ${\mathcal {T}} = \frac{1}{C_{\tau _0, M, r, \nu } } \log (\log (|\Omega _0|) )$;
(ii) if $\Vert \overline{V}(t)\Vert _{r+3,0,\tau (t)} \le \frac{\nu }{4C_{r,\alpha }}$ which is small enough, then (5.2) can be relaxed and replaced by $\Vert \widetilde{\mathcal {V}}_0\Vert _{\frac{3}{2}+\delta ,0,0} \le \frac{\tau _0}{C_{r,\nu ,M}}$, and (5.3) can be improved to ${\mathcal {T}} = \frac{1}{C_{\tau _0, M, r, \nu } } \log (|\Omega _0|)$;
(iii) finally, if the initial condition satisfies $\Vert \overline{{\mathcal {V}}}_0\Vert _{r+3,0,\tau _0} \le \frac{M}{|\Omega _0|}$, (5.2) can be relaxed and replaced by $\Vert \widetilde{\mathcal {V}}_0\Vert _{\frac{3}{2}+\delta ,0,0} \le \frac{\tau _0}{C_{r,\nu ,M}}$, and (5.3) can be improved to ${\mathcal {T}} = \frac{|\Omega _0|^{\frac{1}{2}}}{C_{\tau _0, M, r, \nu } }$.

### Remark 10

Compared to [23], the main improvement in Theorem 5.1 is that the initial data is analytic in the horizontal variables but only $L^2$ in the vertical variable. The main improvements in Theorem 5.2 are points (*ii*) and (*iii*), where the smallness assumption does not depend on $\Omega _0$, and the lifespan is growing faster with respect to $\Omega _0.$ For more details, we refer readers to R3 and R4 in the introduction (pages 2 and 3).

In this section, we focus on equations (2.22)–(2.24), which are equivalent to system (1.1). To prove Theorem 5.1, in section 5.1, we rewrite (2.22)–(2.24) as the perturbation of (4.2)–(4.4). In section 5.2, we establish a series of *a priori* estimates on the solutions to the perturbation system. This together with Proposition 4.2 will finish the proof of Theorem 5.1. In section 5.3, the proof of Theorem 5.2 is provided.

### Remark 11

In this section, we only focus on the long-time existence of the weak solution. By virtue of Theorem 3.2, the weak solution is analytic in all spatial variables.

### The Perturbation System

Denote by

5.5 $$\begin{aligned} \overline{\phi } := \overline{\mathcal {V}} - \overline{V} \qquad \text {and} \qquad \phi _\pm := \mathcal {V}_\pm - V_\pm . \end{aligned}$$

Calculating the difference between (2.22), (2.23) , (2.24) and (4.2), (4.3), (4.4), respectively, leads to

5.6 $$\begin{aligned}&\partial _t \phi _+ + \overline{\phi } \cdot \nabla V_+ + \overline{\phi } \cdot \nabla \phi _+ + \overline{V} \cdot \nabla \phi _+ + \frac{1}{2}(\phi _+ \cdot \nabla )(\overline{V}+i\overline{V}^\perp ) + \frac{1}{2}(\phi _+ \cdot \nabla )(\overline{\phi }+i\overline{\phi }^\perp ) \nonumber \\&\quad + \frac{1}{2}(V_+ \cdot \nabla )(\overline{\phi }+i\overline{\phi }^\perp ) - \nu \partial _{zz} \phi _+ + e^{i\Omega t}\Big (Q_{1,+,+} - {\mathfrak {P}}_0 Q_{1,+,+} - {\mathfrak {P}}_0 Q_{2,+,+} - Q_{3,+,+}\Big ) \nonumber \\&\quad + e^{-i\Omega t} \Big (Q_{1,-,+} - {\mathfrak {P}}_0 Q_{1,-,+} - {\mathfrak {P}}_0 Q_{2,-,+} - Q_{3,-,+}\Big ) + e^{-2i\Omega t} Q_{4,-,+} = 0 , \end{aligned}$$

5.7 $$\begin{aligned}&\partial _t \phi _- + \overline{\phi } \cdot \nabla V_- + \overline{\phi } \cdot \nabla \phi _- + \overline{V} \cdot \nabla \phi _- + \frac{1}{2}(\phi _- \cdot \nabla )(\overline{V}-i\overline{V}^\perp ) + \frac{1}{2}(\phi _- \cdot \nabla )(\overline{\phi }-i\overline{\phi }^\perp )\nonumber \\&\quad + \frac{1}{2}(V_- \cdot \nabla )(\overline{\phi }-i\overline{\phi }^\perp ) - \nu \partial _{zz} \phi _- + e^{-i\Omega t}\Big (Q_{1,-,-} - {\mathfrak {P}}_0 Q_{1,-,-} - {\mathfrak {P}}_0 Q_{2,-,-} - Q_{3,-,-}\Big ) \nonumber \\&\quad + e^{i\Omega t} \Big (Q_{1,+,-} - {\mathfrak {P}}_0 Q_{1,+,-} - {\mathfrak {P}}_0 Q_{2,+,-} - Q_{3,+,-}\Big ) + e^{2i\Omega t}Q_{4,+,-} = 0 , \nonumber \\&\nabla \cdot {\overline{\phi }} = 0 , \qquad \partial _z p = 0, \nonumber \\&\partial _t \overline{\phi } + \overline{\phi }\cdot \nabla \overline{V} + \overline{\phi }\cdot \nabla \overline{\phi } + \overline{V}\cdot \nabla \overline{\phi } + e^{2i\Omega t} {\mathfrak {P}}_0\Big ( Q_{1,+,+} + Q_{2,+,+} \Big )\nonumber \\&\quad + e^{-2i\Omega t} {\mathfrak {P}}_0\Big ( Q_{1,-,-} + Q_{2,-,-} \Big ) + \nabla p = 0, \end{aligned}$$

where

$$\begin{aligned} Q_{1,\pm ,\mp } :=&\phi _\pm \cdot \nabla V_\mp + \phi _\pm \cdot \nabla \phi _\mp + V_\pm \cdot \nabla \phi _\mp + V_\pm \cdot \nabla V_\mp , \\ Q_{2,\pm ,\mp } :=&(\nabla \cdot \phi _\pm ) V_\mp + (\nabla \cdot \phi _\pm ) \phi _\mp + (\nabla \cdot V_\pm ) \phi _\mp + (\nabla \cdot V_\pm ) V_\mp , \\ Q_{3,\pm ,\mp } :=&\left( \int _0^z \nabla \cdot \phi _\pm (\varvec{x},s)ds\right) \partial _z V_\mp +\left( \int _0^z \nabla \cdot \phi _\pm (\varvec{x},s)ds\right) \partial _z \phi _\mp \\&+\left( \int _0^z \nabla \cdot V_\pm (\varvec{x},s)ds\right) \partial _z \phi _\mp +\left( \int _0^z \nabla \cdot V_\pm (\varvec{x},s)ds\right) \partial _z V_\mp , \\ Q_{4,\pm ,\mp } :=&\frac{1}{2}\Big [(\phi _\pm \cdot \nabla )(\overline{V} \mp i\overline{V}^\perp ) + (\phi _\pm \cdot \nabla )(\overline{\phi } \mp i\overline{\phi }^\perp )\\&+ (V_\pm \cdot \nabla )(\overline{\phi } \mp i\overline{\phi }^\perp ) + (V_\pm \cdot \nabla )(\overline{V} \mp i\overline{V}^\perp ) \Big ]. \end{aligned}$$

Recalling that $( \overline{{\mathcal {V}}}, {\mathcal {V}}_\pm )$ and $( {\overline{V}}, V_\pm )$ are complemented with the same initial data. Hence, we have

5.8 $$\begin{aligned} \overline{\phi }|_{t=0}=0 \qquad \text {and} \qquad \phi _\pm |_{t=0} = 0. \end{aligned}$$

### Proof of Theorem 5.1

In this subsection, we prove Theorem 5.1. Thanks to Proposition 4.2, for any given ${\mathcal {T}} \in (0,\infty )$, let $V_\pm$ and $\overline{V}$ be the global solution to equations (4.2)-(4.4) in $L^\infty (0,{\mathcal {T}}; \mathcal {S}\cap \mathcal {S}_{r+2,1,\tau (t)})$ and $L^\infty (0,{\mathcal {T}}; \mathcal {S}\cap \mathcal {S}_{r+3,0,\tau (t)})$ for some $\tau = \tau (t), t \in [0,{\mathcal {T}})$, respectively. Next, we provide the energy estimate in the space $\mathcal {S}_{r,0,\tau (t)}$ for equations (5.6)–(5.7).

After applying similar calculation as in (3.1), we obtain that

5.9 $$\begin{aligned}&\frac{1}{2}\frac{d}{dt} (\Vert \phi _+ \Vert _{r,0,\tau }^2 + \Vert \phi _- \Vert _{r,0,\tau }^2) + \nu (\Vert \partial _z \phi _+ \Vert _{r,0,\tau }^2 + \Vert \partial _z \phi _- \Vert _{r,0,\tau }^2)= \dot{\tau } (\Vert A^{r+\frac{1}{2}} e^{\tau A} \phi _+ \Vert ^2 + \Vert A^{r+\frac{1}{2}} e^{\tau A} \phi _- \Vert ^2) \nonumber \\ \quad&- \Big \langle \overline{\phi } \cdot \nabla V_+ + \overline{\phi } \cdot \nabla \phi _+ + \overline{V} \cdot \nabla \phi _+ + \frac{1}{2}(\phi _+ \cdot \nabla )(\overline{V}+i\overline{V}^\perp ) + \frac{1}{2}(\phi _+ \cdot \nabla )(\overline{\phi }+i\overline{\phi }^\perp ) \nonumber \\ \quad&+ \frac{1}{2}(V_+ \cdot \nabla )(\overline{\phi }+i\overline{\phi }^\perp ) + e^{i\Omega t}\Big (Q_{1,+,+} - Q_{3,+,+}\Big ) + e^{-i\Omega t} \Big (Q_{1,-,+} - Q_{3,-,+}\Big ) + e^{-2i\Omega t}Q_{4,-,+} , \phi _+ \Big \rangle \nonumber \\ \quad&- \Big \langle \overline{\phi } \cdot \nabla V_- + \overline{\phi } \cdot \nabla \phi _- + \overline{V} \cdot \nabla \phi _- + \frac{1}{2}(\phi _- \cdot \nabla )(\overline{V}-i\overline{V}^\perp ) + \frac{1}{2}(\phi _- \cdot \nabla )(\overline{\phi }-i\overline{\phi }^\perp ) \nonumber \\ \quad&+ \frac{1}{2}(V_- \cdot \nabla )(\overline{\phi }-i\overline{\phi }^\perp ) + e^{-i\Omega t}\Big (Q_{1,-,-} - Q_{3,-,-}\Big ) + e^{i\Omega t} \Big (Q_{1,+,-} - Q_{3,+,-}\Big ) + e^{2i\Omega t}Q_{4,+,-} , \phi _- \Big \rangle \nonumber \\ \quad&- \underbrace{\Big \langle A^r e^{\tau A} (\overline{\phi }\cdot \nabla V_+), A^r e^{\tau A} \phi _+ \Big \rangle }_\mathrm {Tp2} - \underbrace{\Big \langle A^r e^{\tau A} (\overline{\phi }\cdot \nabla V_-), A^r e^{\tau A} \phi _- \Big \rangle }_\mathrm {Tp2} \nonumber \\ \quad&- \underbrace{\Big \langle A^r e^{\tau A} (\overline{\phi }\cdot \nabla \phi _+), A^r e^{\tau A} \phi _+ \Big \rangle }_\mathrm {Tp1}- \underbrace{\Big \langle A^r e^{\tau A} (\overline{\phi }\cdot \nabla \phi _-), A^r e^{\tau A} \phi _- \Big \rangle }_\mathrm {Tp1} \nonumber \\ \quad&- \underbrace{\Big \langle A^r e^{\tau A} (\overline{V}\cdot \nabla \phi _+), A^r e^{\tau A} \phi _+ \Big \rangle }_\mathrm {Tp4} - \underbrace{\Big \langle A^r e^{\tau A} (\overline{V}\cdot \nabla \phi _-), A^r e^{\tau A} \phi _- \Big \rangle }_\mathrm {Tp4} \nonumber \\ \quad&- \underbrace{\Big \langle A^r e^{\tau A} (\phi _+\cdot \nabla (\overline{V}+i\overline{V}^\perp )), A^r e^{\tau A} \phi _+ \Big \rangle }_\mathrm {Tp2} - \underbrace{\Big \langle A^r e^{\tau A} (\phi _- \cdot \nabla (\overline{V}-i\overline{V}^\perp )), A^r e^{\tau A} \phi _- \Big \rangle }_\mathrm {Tp2} \nonumber \\ \quad&- \underbrace{\Big \langle A^r e^{\tau A} (\phi _+\cdot \nabla (\overline{\phi }+i\overline{\phi }^\perp )), A^r e^{\tau A} \phi _+ \Big \rangle }_\mathrm {Tp1} - \underbrace{\Big \langle A^r e^{\tau A} (\phi _- \cdot \nabla (\overline{\phi }-i\overline{\phi }^\perp )), A^r e^{\tau A} \phi _- \Big \rangle }_\mathrm {Tp1} \nonumber \\ \quad&- \underbrace{\Big \langle A^r e^{\tau A} (V_+\cdot \nabla (\overline{\phi }+i\overline{\phi }^\perp )), A^r e^{\tau A} \phi _+ \Big \rangle }_\mathrm {Tp4} - \underbrace{\Big \langle A^r e^{\tau A} (V_- \cdot \nabla (\overline{\phi }-i\overline{\phi }^\perp )), A^r e^{\tau A} \phi _- \Big \rangle }_\mathrm {Tp4} \nonumber \\ \quad&- \underbrace{e^{i\Omega t}\Big (\Big \langle A^r e^{\tau A}(Q_{1,+,+} - Q_{3,+,+}), A^r e^{\tau A} \phi _+ \Big \rangle + \Big \langle A^r e^{\tau A}(Q_{1,+,-} - Q_{3,+,-}), A^r e^{\tau A} \phi _-\Big \rangle \Big ) }_{\mathrm {Tp1}, \cdots , \mathrm {Tp5}} \nonumber \\ \quad&- \underbrace{e^{-i\Omega t}\Big (\Big \langle A^r e^{\tau A}(Q_{1,-,+} - Q_{3,-,+}), A^r e^{\tau A} \phi _+ \Big \rangle + \Big \langle A^r e^{\tau A}(Q_{1,-,-} - Q_{3,-,-}), A^r e^{\tau A} \phi _- \Big \rangle \Big ) }_{\mathrm {Tp1} , \cdots , \mathrm {Tp5}} \nonumber \\ \quad&-\underbrace{e^{2i\Omega t}\Big \langle A^r e^{\tau A}Q_{4,+,-}, A^r e^{\tau A} \phi _- \Big \rangle }_{\mathrm {Tp1}, \mathrm {Tp2},\mathrm {Tp4},\mathrm {Tp5}} - \underbrace{e^{-2i\Omega t}\Big \langle A^r e^{\tau A}Q_{4,-,+}, A^r e^{\tau A} \phi _+ \Big \rangle }_{\mathrm {Tp1} ,\mathrm {Tp2},\mathrm {Tp4},\mathrm {Tp5}}, \end{aligned}$$

and

5.10 $$\begin{aligned} \frac{1}{2} \frac{d}{dt} \Vert A^r e^{\tau A} \overline{\phi } \Vert ^2 =&\dot{\tau } \Vert A^{r+\frac{1}{2}} e^{\tau A} \overline{\phi } \Vert ^2 \nonumber \\&- \underbrace{\Big \langle A^r e^{\tau A} (\overline{\phi }\cdot \nabla \overline{V}), A^r e^{\tau A} \overline{\phi } \Big \rangle }_{\mathrm {Tp2}} - \underbrace{\Big \langle A^r e^{\tau A} (\overline{\phi }\cdot \nabla \overline{\phi }), A^r e^{\tau A} \overline{\phi } \Big \rangle }_\mathrm {Tp1} \nonumber \\&- \underbrace{\Big \langle A^r e^{\tau A} (\overline{V}\cdot \nabla \overline{\phi }), A^r e^{\tau A} \overline{\phi } \Big \rangle }_\mathrm {Tp4} - \underbrace{e^{2i\Omega t}\Big \langle A^r e^{\tau A}(Q_{1,+,+} + Q_{2,+,+}), A^r e^{\tau A} \overline{\phi }\Big \rangle }_{\mathrm {Tp1} , \mathrm {Tp2}, \mathrm {Tp4},\mathrm {Tp5} } \nonumber \\&- \underbrace{e^{-2i\Omega t}\Big \langle A^r e^{\tau A}(Q_{1,-,-} + Q_{2,-,-}), A^r e^{\tau A} \overline{\phi }\Big \rangle }_{\mathrm {Tp1} , \mathrm {Tp2}, \mathrm {Tp4},\mathrm {Tp5} }, \end{aligned}$$

where we have applied Lemmas 2.2–2.3. It is easy to verify from (5.7) and (5.8) that

$$\begin{aligned} \int _{\mathbb {T}^2} \overline{\phi }(\varvec{x},t) \, d\varvec{x} = \int _{\mathbb {T}^2} \overline{\phi }(\varvec{x},t)|_{t=0} \, d\varvec{x} = 0, \end{aligned}$$

and therefore, applying the Poincaré inequality yields

5.11 $$\begin{aligned} \Vert \overline{\phi }\Vert \le \Vert A^r e^{\tau A}\overline{\phi }\Vert \qquad \text {and} \qquad \Vert \overline{\phi }\Vert _{r,0,\tau } \le C \Vert A^r e^{\tau A}\overline{\phi }\Vert . \end{aligned}$$

In (5.9) and (5.10), we have labeled five types of terms by $\mathrm {Tp1},\cdots , \mathrm {Tp5}$, which we will present the estimates. The rest lower order terms can be estimated in a similar manner and will be omitted. Temporally, let *V* denote $V_\pm$ and $\overline{V}$, and $\phi$ denote $\phi _\pm$ and $\overline{\phi }$. The aforementioned five types of terms are described in the following:

- Type 1 (labeled as $\mathrm {Tp1}$): terms that are trilinear in $\phi$, e.g.,
  $$\begin{aligned} e^{\mathrm {j} i \Omega t} \Big \langle A^r e^{\tau A} \bigl ( ({\phi }\cdot \nabla ) {\phi }\bigr ), A^r e^{\tau A} {\phi } \Big \rangle , \qquad e^{\mathrm {j} i \Omega t} \Big \langle A^r e^{\tau A} \bigl ( (\nabla \cdot {\phi }) \phi \bigr ), A^r e^{\tau A} {\phi } \Big \rangle ,\\ \text {and} \qquad e^{\mathrm {j} i \Omega t}\Big \langle A^r e^{\tau A} \bigl (\int _0^z (\nabla \cdot \phi (\varvec{x},s)) \,ds \partial _z \phi \bigr ) , A^r e^{\tau A} \phi \Big \rangle , \qquad \mathrm {j} = 0,\pm 1, \pm 2; \end{aligned}$$
- Type 2 (labeled as $\mathrm {Tp2}$): terms that are bilinear in $\phi$ with no derivative of $\phi$, e.g.,
  $$\begin{aligned} e^{\mathrm {j} i \Omega t} \Big \langle A^r e^{\tau A} \bigl ( ({\phi }\cdot \nabla ) {V}\bigr ), A^r e^{\tau A} {\phi } \Big \rangle \qquad \text {and} \\ e^{\mathrm {j} i \Omega t} \Big \langle A^r e^{\tau A} \bigl ( (\nabla \cdot {V}) \phi \bigr ), A^r e^{\tau A} {\phi } \Big \rangle , \qquad \mathrm {j} = 0,\pm 1, \pm 2; \end{aligned}$$
- Type 3 (labeled as $\mathrm {Tp3}$): terms that are bilinear in $\phi$ and a vertical derivative of $\phi$, e.g.,
  $$\begin{aligned} e^{\mathrm {j} i \Omega t}\Big \langle A^r e^{\tau A} \bigl ( \int _0^z (\nabla \cdot V(\varvec{x},s)) \,ds \partial _z \phi \bigr ) , A^r e^{\tau A} \phi \Big \rangle , \qquad \mathrm {j} = 0,\pm 1, \pm 2; \end{aligned}$$
- Type 4 (labeled as $\mathrm {Tp4}$): terms that are bilinear in $\phi$ and a horizontal derivative of $\phi$, e.g.,
  $$\begin{aligned} e^{\mathrm {j} i \Omega t} \Big \langle A^r e^{\tau A} \bigl ( ({V}\cdot \nabla ) {\phi }\bigr ), A^r e^{\tau A} {\phi } \Big \rangle , \qquad e^{\mathrm {j} i \Omega t} \Big \langle A^r e^{\tau A} \bigl ( (\nabla \cdot {\phi }) V \bigr ), A^r e^{\tau A} {\phi } \Big \rangle ,\\ \text {and} \qquad e^{\mathrm {j} i \Omega t}\Big \langle A^r e^{\tau A} \bigl ( \int _0^z (\nabla \cdot \phi (\varvec{x},s)) \,ds \partial _z V \bigr ) , A^r e^{\tau A} \phi \Big \rangle , \qquad \mathrm {j} = 0,\pm 1, \pm 2; \end{aligned}$$
- Type 5 (labeled as $\mathrm {Tp5}$): terms that are linear in $\phi$, e.g.,
  $$\begin{aligned} e^{\mathrm {j} i \Omega t} \Big \langle A^r e^{\tau A} \bigl ( ({V}\cdot \nabla ) {V}\bigr ), A^r e^{\tau A} {\phi } \Big \rangle , \qquad e^{\mathrm {j} i \Omega t} \Big \langle A^r e^{\tau A} \bigl ( (\nabla \cdot {V}) V \bigr ), A^r e^{\tau A} {\phi } \Big \rangle ,\\ \text {and} \qquad e^{\mathrm {j} i \Omega t} \Big \langle A^r e^{\tau A} \bigl ( \int _0^z (\nabla \cdot V(\varvec{x},s)) \,ds \partial _z V\bigr ) , A^r e^{\tau A} \phi \Big \rangle , \qquad \mathrm {j} = \pm 1, \pm 2. \end{aligned}$$

#### Estimates of Type 1 – Type 4 Terms

We start with Type 1 terms. Applying Lemmas A.1–A.3 yields

$$\begin{aligned} |\mathrm {Tp1}| \le&C_r \int _0^1 \underbrace{\Vert A^{r+\frac{1}{2}} e^{\tau A} \phi (z) \Vert _{L^2({\mathbb {T}}^2)}^2}_{L^1 ~ \text {in} ~ z} \underbrace{ \bigl (\Vert A^{r} e^{\tau A} \phi (z) \Vert _{L^2({\mathbb {T}}^2)} + \Vert \phi (z) \Vert _{L^2({\mathbb {T}}^2)} \bigr )}_{L^\infty ~ \text {in} ~ z } \,dz \\&+ C_r \Vert A^{r+\frac{1}{2}}e^{\tau A}\phi \Vert ^2 \Vert \partial _z \phi \Vert _{r,0, \tau } \le C_r \Vert A^{r+\frac{1}{2}}e^{\tau A} \phi \Vert ^2 \Vert \phi \Vert _{r,1,\tau }, \end{aligned}$$

where we have used the embedding $L^\infty _z\hookrightarrow H^1_z$ in the *z*-variable and the Hölder inequality. Notice that, for $\phi = {\overline{\phi }}$, the estimate is similar with obvious modification. Therefore, hereafter, unless pointed out explicitly, we omit the estimates in the case of $\phi = {\overline{\phi }}$ and, similarly, $V = {\overline{V}}$.

Similarly, applying Lemma 2.1 to Types 2 and 3 terms yields

$$\begin{aligned} |\mathrm {Tp2}| \le&C_r \int _0^1 \underbrace{\bigl ( \Vert A^{r+1}e^{\tau A} V (z) \Vert _{L^2({\mathbb {T}}^2)} + \Vert V (z)) \Vert _{L^2({\mathbb {T}}^2)} \bigr )}_{L^\infty ~ \text {in} ~ z} \underbrace{ \bigl (\Vert A^{r}e^{\tau A} \phi (z) \Vert _{L^2({\mathbb {T}}^2)} + \Vert \phi (z)) \Vert _{L^2({\mathbb {T}}^2)} \bigr )^2}_{L^1 ~ \text {in} ~ z} \,dz \\ \le&C_r \Vert V \Vert _{r+1, 1, \tau } \Vert \phi \Vert _{r,0,\tau }^2 \qquad \qquad \qquad \text {and}\\ |\mathrm {Tp3} | \le&C_r \int _0^1 \biggl [\underbrace{\biggl ( \int _0^z \Vert A^{r+1} e^{\tau A} V (s) \Vert _{L^2({\mathbb {T}}^2)} + \Vert V (s) \Vert _{L^2({\mathbb {T}}^2)} \,ds \biggr )}_{L^\infty ~ \text {in} ~ z } \underbrace{ \biggl ( \Vert A^{r} e^{\tau A} \partial _z \phi (z) \Vert _{L^2({\mathbb {T}}^2)} + \Vert \partial _z \phi (z) \Vert _{L^2({\mathbb {T}}^2)} \biggr )}_{L^2 ~ \text {in} ~ z} \\&\times \underbrace{\biggl ( \Vert A^{r} e^{\tau A} \phi (z) \Vert _{L^2({\mathbb {T}}^2)} + \Vert \phi (z) \Vert _{L^2({\mathbb {T}}^2)} \biggr )}_{L^2 ~ \text {in} ~ z} \biggr ]\,dz \le C_r \Vert V\Vert _{r+1,0,\tau } \Vert \partial _z \phi \Vert _{r,0,\tau } \Vert \phi \Vert _{r,0,\tau } \\ \le&\frac{\nu }{2} \Vert \partial _z \phi \Vert _{r,0,\tau }^2 + { \dfrac{C_{r}}{\nu }} \Vert V\Vert _{r+1,0,\tau }^2 \Vert \phi \Vert _{r,0,\tau } ^2. \end{aligned}$$

In order to estimate Type 4 terms, notice that $\mathrm {Tp4}$ can be written as, with abuse of notations,

$$\begin{aligned} \mathrm {Tp4} = \mathrm {Tp4}_1 + \mathrm {Tp4}_2, \end{aligned}$$

where

$$\begin{aligned}&\begin{aligned} \mathrm {Tp4}_1 :=&\, e^{\mathrm j i\Omega t} \Big \langle (V \cdot \nabla ) A^r e^{\tau A} \phi , A^r e^{\tau A} \phi \Big \rangle + e^{\mathrm j i\Omega t} \Big \langle (\nabla \cdot A^r e^{\tau A} \phi ) V, A^r e^{\tau A} \phi \Big \rangle \\&+ e^{\mathrm j i\Omega t} \Big \langle \int _0^z \bigl (\nabla \cdot A^r e^{\tau A} \phi (s)\bigr ) \,ds \partial _z V, A^r e^{\tau A} \phi \Big \rangle , \end{aligned}\\&\begin{aligned} \mathrm {Tp4}_2 := \,&e^{\mathrm j i\Omega t} \Big \langle A^r e^{\tau A} \bigl ( ( V \cdot \nabla ) \phi \bigr ) - (V \cdot \nabla ) A^r e^{\tau A} \phi , A^r e^{\tau A} \phi \Big \rangle \\&+ e^{\mathrm j i\Omega t} \Big \langle A^r e^{\tau A} \bigl ( ( \nabla \cdot \phi ) V \bigr ) - (\nabla \cdot A^r e^{\tau A} \phi ) V, A^r e^{\tau A} \phi \Big \rangle \\&+ e^{\mathrm j i\Omega t} \Big \langle A^r e^{\tau A} \bigl ( \int _0^z( \nabla \cdot \phi (s) ) \,ds \partial _z V \bigr ) - \int _0^z \bigl (\nabla \cdot A^r e^{\tau A} \phi (s)\bigr ) \,ds \partial _z V, A^r e^{\tau A} \phi \Big \rangle . \end{aligned} \end{aligned}$$

Observing from (5.9) and (5.10), only for $V = V_\pm$, $\mathrm {Tp4}_1$ is nontrivial. Therefore, after substituting the inequality $|\alpha |^{\frac{1}{2}} \le |\beta |^{\frac{1}{2}} + |\xi |^{\frac{1}{2}}$ for $\alpha + \beta = \xi$ in the Fourier representation of $\mathrm {Tp4}_1$ (see the proof of Lemma A.2 in the appendix), one can obtain that, for any $\delta \in (0,1)$,

$$\begin{aligned}&| \mathrm {Tp4}_1| \le \Big | \Big \langle (A^{\frac{1}{2}} V_\pm \cdot \nabla ) A^{r-\frac{1}{2}} e^{\tau A} \phi , A^r e^{\tau A} \phi \Big \rangle \Big | + \Big | \Big \langle (V_\pm \cdot \nabla ) A^{r-\frac{1}{2}} e^{\tau A} \phi , A^{r+\frac{1}{2}} e^{\tau A} \phi \Big \rangle \Big | \\&\quad + \Big | \Big \langle (\nabla \cdot A^{r-\frac{1}{2}} e^{\tau A} \phi ) A^{\frac{1}{2}} V_\pm , A^r e^{\tau A} \phi \Big \rangle \Big | + \Big | \Big \langle (\nabla \cdot A^{r-\frac{1}{2}} e^{\tau A} \phi ) V_\pm , A^{r+\frac{1}{2}} e^{\tau A} \phi \Big \rangle \Big | \\&\quad + \Big | \Big \langle \int _0^z \bigl (\nabla \cdot A^{r-\frac{1}{2}} e^{\tau A} \phi (s)\bigr ) \,ds \partial _z A^{\frac{1}{2}} V_\pm , A^r e^{\tau A} \phi \Big \rangle \Big | + \Big | \Big \langle \int _0^z \bigl (\nabla \cdot A^{r-\frac{1}{2}} e^{\tau A} \phi (s)\bigr ) \,ds \partial _z V_\pm , A^{r+\frac{1}{2}} e^{\tau A} \phi \Big \rangle \Big |\\&\le C_r \int _0^1 \underbrace{\bigl ( \Vert A^{\frac{1}{2}}V_\pm (z)\Vert _{H^{1+\delta }({\mathbb {T}}^2)}+\Vert V_\pm (z)\Vert _{H^{1+\delta }({\mathbb {T}}^2)} \bigr )}_{L^\infty ~ \text {in} ~ z} \underbrace{\Vert A^{r+\frac{1}{2}} e^{\tau A} \phi (z) \Vert _{L^2({\mathbb {T}}^2)}^2}_{L^1 ~ \text {in} ~ z} \,dz \\&\quad + C_r \int _0^1 \biggl [\underbrace{\int _0^z \Vert A^{r+\frac{1}{2}} e^{\tau A} \phi (s) \Vert _{L^2({\mathbb {T}}^2)} \,ds}_{L^\infty ~ \text {in} ~ z} \times \underbrace{\Vert A^{r+\frac{1}{2}} e^{\tau A} \phi (z) \Vert _{L^2({\mathbb {T}}^2)}}_{L^2 ~ \text {in} ~ z} \\&\qquad \qquad \times \underbrace{\bigl ( \Vert \partial _z A^{\frac{1}{2}} V_\pm (z) \Vert _{H^{1+\delta }({\mathbb {T}}^2)} + \Vert \partial _z V_\pm (z)\Vert _{H^{1+\delta }({\mathbb {T}}^2)} \bigr )}_{L^2 ~ \text {in} ~ z} \biggr ]\,dz \le C_r \Vert V_\pm \Vert _{\frac{3}{2}+\delta ,1,0} \Vert A^{r+\frac{1}{2}} e^{\tau A} \phi \Vert ^2, \end{aligned}$$

where we have applied the Sobolev embedding inequality and the Hölder inequality. Meanwhile, applying Lemmas A.4–A.6 to $\mathrm {Tp4}_2$ yields

$$\begin{aligned} |\mathrm {Tp4}_2| \le&C_r \int _0^1 \Vert A^r \phi (z) \Vert _{L^2({\mathbb {T}}^2)}^2 \Vert A^r V(z) \Vert _{L^2({\mathbb {T}}^2)} \,dz \\&+ C_r \tau \int _0^1 \Vert A^{r+\frac{1}{2}}e^{\tau A} \phi (z) \Vert _{L^2({\mathbb {T}}^2)}^2 \Vert A^{r+\frac{1}{2}}e^{\tau A} V(z) \Vert _{L^2({\mathbb {T}}^2)} \,dz \\&+ C_r \Vert A^r \partial _z V \Vert \Vert A^r \phi \Vert ^2 + C_r \tau \Vert A^{r+\frac{1}{2}}e^{\tau A} \partial _z V \Vert \Vert A^{r+\frac{1}{2}} e^{\tau A} \phi \Vert ^2 \\ \le&C_r \Vert V \Vert _{r,1,\tau } \Vert \phi \Vert _{r,0,\tau }^2 + C_r \tau \Vert V\Vert _{r+\frac{1}{2},1,\tau } \Vert A^{r+\frac{1}{2}} e^{\tau A} \phi \Vert ^2. \end{aligned}$$

##### Remark 12

For the interested readers, we refer to [23] for an alternative estimate of $\mathrm {Tp4}_1$, where some cancellations are taking care of. However, in this paper, such cancellations are not necessary and thus omitted. Notably, the terms $\Vert V_\pm \Vert _{\frac{3}{2}+\delta ,1,0}$ in the estimate of $\mathrm {Tp4}_1$ is the reason for the requirement (5.2).

#### Estimates of Type 5 Terms

In this case, $\mathrm j \ne 0$ and $e^{\mathrm j \Omega i t} = \dfrac{1}{\mathrm j \Omega i} \dfrac{d}{dt} e^{\mathrm j \Omega i t}$. Therefore, $\mathrm {Tp5}$ can be written as, with abuse of notations,

$$\begin{aligned} \mathrm {Tp5} = \dfrac{1}{\Omega } \partial _t N + \dfrac{1}{\Omega } R, \end{aligned}$$

with

5.12 $$\begin{aligned}&\begin{aligned} N :=&\dfrac{e^{\mathrm {j}\Omega i t}}{\mathrm j i} \biggl [\Big \langle A^r e^{\tau A} \bigl ( ( V \cdot \nabla ) V \bigr ) , A^r e^{\tau A} \phi \Big \rangle + \Big \langle A^r e^{\tau A} \bigl ( ( \nabla \cdot V ) V \bigr ) , A^r e^{\tau A} \phi \Big \rangle \\&+ \Big \langle A^r e^{\tau A} \bigl ( \int _0^z (\nabla \cdot V (s)) \,ds \partial _z V \bigr ) , A^r e^{\tau A} \phi \Big \rangle \biggr ], \end{aligned} \end{aligned}$$

5.13 $$\begin{aligned}&\begin{aligned} R :=&\dfrac{e^{\mathrm {j}\Omega i t}}{\mathrm j i} \biggl [\underbrace{\partial _t \Big \langle A^r e^{\tau A} \bigl ( ( V \cdot \nabla ) V \bigr ) , A^r e^{\tau A} \phi \Big \rangle }_{=:R_1} + \underbrace{\partial _t \Big \langle A^r e^{\tau A} \bigl ( ( \nabla \cdot V ) V \bigr ) , A^r e^{\tau A} \phi \Big \rangle }_{=:R_2} \\&+ \underbrace{\partial _t \Big \langle A^r e^{\tau A} \bigl ( \int _0^z (\nabla \cdot V (s)) \,ds \partial _z V \bigr ) , A^r e^{\tau A} \phi \Big \rangle }_{=:R_3} \biggr ]. \end{aligned} \end{aligned}$$

It is straightforward to check that

5.14 $$\begin{aligned} N \le C_r \Vert V \Vert _{r,1,\tau } \Vert V \Vert _{r+1,0,\tau }\Vert \phi \Vert _{r,0,\tau }. \end{aligned}$$

Meanwhile, one has

$$\begin{aligned} R_1 =&\,2 {\dot{\tau }} \Big \langle A^{r+1} e^{\tau A} \bigl ( (V \cdot \nabla ) V\bigr ), A^r e^{\tau A} \phi \Big \rangle + \Big \langle A^r e^{\tau A} \partial _t \bigl ( ( V\cdot \nabla ) V \bigr ) , A^r e^{\tau A}\phi \Big \rangle \\&+ \Big \langle A^r e^{\tau A} \bigl ( ( V\cdot \nabla ) V \bigr ) , A^r e^{\tau A}\partial _t\phi \Big \rangle =: R_{1,1} + R_{1,2} + R_{1,3}. \end{aligned}$$

It follows that, thanks to Lemma 2.1 and similar arguments as in section 5.2.1,

5.15 $$\begin{aligned} R_{1,1} \le C_r |{\dot{\tau }}| \Vert V \Vert _{r+1,1,\tau } \Vert V \Vert _{r+2,0,\tau } \Vert \phi \Vert _{r,0,\tau }. \end{aligned}$$

After applying the Leray projection (2.12) to (4.4), together with (4.2) and (4.3), for $V = V_\pm$ or ${\overline{V}}$, one has

5.16 $$\begin{aligned} \partial _t V - \underbrace{\nu \partial _{zz}V}_{\text {for} ~ V = V_\pm } = {\mathcal {B}}( V, \nabla V ). \end{aligned}$$

Here we use ${\mathcal {B}}$ to represent a generic bilinear term with respect to both of its arguments. With such notations, after applying integration by parts, one can derive

5.17 $$\begin{aligned} \begin{aligned} R_{1,2} =&- 2 \nu \Big \langle A^r e^{\tau A} \bigl ( (\partial _z V \cdot \nabla ) \partial _z V \bigr ) , A^r e^{\tau A} \phi \Big \rangle - \nu \Big \langle A^r e^{\tau A} \bigl ( (\partial _z V \cdot \nabla ) V +( V \cdot \nabla ) \partial _z V \bigr ) , A^r e^{\tau A} \partial _z \phi \Big \rangle \\&+ \Big \langle A^r e^{\tau A} \bigl ( ({\mathcal {B}}(V,\nabla V) \cdot \nabla ) V + (V \cdot \nabla ) {\mathcal {B}}(V, \nabla V) \bigr ) , A^r e^{\tau A} \phi \Big \rangle \\ \le&C_{r}{(1+\nu )}\Big ( \Vert V \Vert _{r,1,\tau } \Vert V \Vert _{r+1,1,\tau } \Vert \phi \Vert _{r,1,\tau } + \Vert V \Vert _{r,0,\tau } \Vert V \Vert _{r+1,1,\tau }^2 \Vert \phi \Vert _{r,0,\tau } + \Vert V\Vert _{r+1,1,\tau }^2 \Vert V\Vert _{r+2,0,\tau } \Vert \phi \Vert _{r,0,\tau }\Big ), \end{aligned} \end{aligned}$$

where we have applied Lemma 2.1 and similar arguments as in section 5.2.1. Similarly, according to (5.6)–(5.7), for $\phi = \phi _\pm$ or ${\overline{\phi }}$, one has, with abuse of notations

5.18 $$\begin{aligned} \partial _t \phi - \underbrace{\bigl ( \nu \partial _{zz} \phi + e^{\mathrm {j}\Omega i t }(\int _0^z \nabla \cdot (\phi + V)(s) \,ds) \partial _z (\phi + V) \bigr )}_{\text {for} \quad \phi = \phi _\pm \quad \text {and} \quad V = V_\pm } = \sum _{ \mathrm A, \mathrm B \in \lbrace \phi , V \rbrace }{\mathcal {B}}( \mathrm A, \nabla \mathrm B ). \end{aligned}$$

Therefore, $R_{1,3}$ can be estimated as

5.19 $$\begin{aligned} \begin{aligned} R_{1,3} =&- \nu \Big \langle A^r e^{\tau A} \bigl ( (\partial _z V \cdot \nabla ) V + (V \cdot \nabla ) \partial _z V \bigr ), A^r e^{\tau A} \partial _z \phi \Big \rangle \\&- e^{\mathrm j\Omega i t} \Big \langle A^{r+1} e^{\tau A} \bigl ( (V \cdot \nabla ) V \bigr ), A^{r-1} e^{\tau A} \bigl ( (\int _0^z \nabla \cdot (\phi + V)(s) \,ds) \partial _z ( \phi + V) \bigr ) \Big \rangle \\&- \sum _{\mathrm A, \mathrm B \in \lbrace \phi , V \rbrace } \Big \langle A^{r+1} e^{\tau A} \bigl ( (V \cdot \nabla ) V \bigr ), A^{r-1} e^{\tau A} {\mathcal {B}}( \mathrm A,\nabla \mathrm B ) \Big \rangle \\ \le&C_{r}{\nu } \Vert V \Vert _{r,1,\tau } \Vert V\Vert _{r+1,1,\tau } \Vert \partial _z \phi \Vert _{r,0,\tau } \\&+ C_r \Vert V \Vert _{r+1,1,\tau } \Vert V \Vert _{r+2,0,\tau } (\Vert \phi \Vert _{r-1,1,\tau } + \Vert V\Vert _{r-1,1,\tau } )( \Vert \phi \Vert _{r,0,\tau } + \Vert V \Vert _{r,0, \tau } ). \end{aligned} \end{aligned}$$

The estimate of $R_2$ is the same as $R_1$ (see (5.15), (5.17), and (5.19)). To estimate $R_3$, one has, after applying integration by parts,

$$\begin{aligned} R_3 =&\,2 {\dot{\tau }} \Big \langle A^{r+1} e^{\tau A} \bigl ( \int _0^z (\nabla \cdot V (s) ) \,ds \partial _z V \bigr ), A^r e^{\tau A} \phi \Big \rangle - \Big \langle A^{r} e^{\tau A} \partial _t \bigl ( (\nabla \cdot V ) V \bigr ), A^r e^{\tau A} \phi \Big \rangle \\&- \Big \langle A^{r} e^{\tau A} \partial _t \bigl ( \int _0^z (\nabla \cdot V (s) ) \,ds V \bigr ), A^r e^{\tau A} \partial _z\phi \Big \rangle + \Big \langle A^r e^{\tau A} \bigl ( \int _0^z (\nabla \cdot V (s) ) \,ds \partial _z V \bigr ), A^r e^{\tau A} \partial _t \phi \Big \rangle \\ =:&\, R_{3,1} + R_{3,2} + R_{3,3} + R_{3,4}. \end{aligned}$$

As before,

5.20 $$\begin{aligned} R_{3,1} \le C_r |{\dot{\tau }}| \Vert V \Vert _{r+2,0,\tau } \Vert V\Vert _{r+1,1,\tau } \Vert \phi \Vert _{r,0,\tau }. \end{aligned}$$

The estimate of $R_{3,2}$ is the same as that of $R_{1,2}$ in (5.17). Meanwhile, substituting representation (5.16) in $R_{3,3}$ leads to

5.21 $$\begin{aligned} \begin{aligned} R_{3,3} =&- \Big \langle A^r e^{\tau A} \bigl ( \int _0^z (\nabla \cdot \partial _t V (s)) \,ds V \bigr ), A^r e^{\tau A} \partial _z \phi \Big \rangle - \Big \langle A^r e^{\tau A} \bigl ( \int _0^z (\nabla \cdot V (s)) \,ds \partial _t V \bigr ), A^r e^{\tau A} \partial _z \phi \Big \rangle \\ =&- \Big \langle A^r e^{\tau A} \bigl ( \int _0^z (\nabla \cdot (\nu \partial _{zz}V + {\mathcal {B}}(V,\nabla V) ) (s)) \,ds V \bigr ), A^r e^{\tau A} \partial _z \phi \Big \rangle \\&- \Big \langle A^r e^{\tau A} \bigl ( \int _0^z (\nabla \cdot V (s)) \,ds (\nu \partial _{zz}V + {\mathcal {B}}(V,\nabla V) ) \bigr ), A^r e^{\tau A} \partial _z \phi \Big \rangle \\ \le&C_{r} \bigl ( {\nu } \Vert V\Vert _{r+1,0,\tau } \Vert V\Vert _{r,2,\tau } + \Vert V\Vert _{r+1,0,\tau } \Vert V\Vert _{r,1,\tau } \Vert V\Vert _{r+2,0,\tau } \bigr ) \Vert \partial _z \phi \Vert _{r,0,\tau }. \end{aligned} \end{aligned}$$

After substituting (5.18), $R_{3,4}$ can be estimated as

5.22 $$\begin{aligned} \begin{aligned} R_{3,4} =&- \nu \Big \langle A^r e^{\tau A} \bigl ( \int _0^z (\nabla \cdot V (s) ) \,ds \partial _{zz} V \bigr ), A^r e^{\tau A} \partial _z \phi \Big \rangle - \nu \Big \langle A^r e^{\tau A} \bigl ( (\nabla \cdot V ) \partial _z V \bigr ), A^r e^{\tau A} \partial _z \phi \Big \rangle \\&- e^{\mathrm j \Omega i t } \Big \langle A^{r+1} e^{\tau A} \bigl ( \int _0^z (\nabla \cdot V (s) ) \,ds \partial _z V \bigr ), A^{r-1} e^{\tau A} \bigl [\bigl ( \int _0^z \nabla \cdot (\phi + V )(s) \,ds \bigr ) \partial _z (\phi + V ) \bigr ]\Big \rangle \\&- \sum _{\mathrm A, \mathrm B \in \lbrace \phi , V \rbrace }\Big \langle A^{r+1} e^{\tau A} \bigl ( \int _0^z (\nabla \cdot V (s) ) \,ds \partial _z V \bigr ), A^{r-1} e^{\tau A} {\mathcal {B}}(\mathrm A, \nabla \mathrm B) \Big \rangle \\ \le&C_{r} {\nu } \Vert V \Vert _{r+1,0,\tau } \Vert V\Vert _{r,2,\tau } \Vert \partial _z \phi \Vert _{r,0,\tau } \\&+ C_r \Vert V \Vert _{r+2,0,\tau } \Vert V \Vert _{r+1,1,\tau } (\Vert \phi \Vert _{r,0,\tau } + \Vert V \Vert _{r,0,\tau } )(\Vert \phi \Vert _{r-1,1,\tau } + \Vert V \Vert _{r-1,1,\tau } ). \end{aligned} \end{aligned}$$

We emphasize that, in the estimates above, we do not distinguish $V_\pm$ and ${\overline{V}}$, $\phi _\pm$ and ${\overline{\phi }}$, i.e., we treat all *V* and $\phi$ as if they are three-dimensional. The estimates in the case when they are two-dimensional are similar with obvious modifications, and thus omitted. Consequently, combining (5.15)–(5.22) leads to the estimate of *R*.

#### Finishing of Proof of Theorem 5.1

Without loss of generality, we assume $| \Omega | > 1$. Combining the estimates in subsections 5.2.1 and 5.2.2, from (5.9) and (5.10), yields, thanks to (5.11) and the Young inequality,

5.23 $$\begin{aligned} \begin{aligned}&\frac{d}{dt} F + \nu H \le \Big [ \dot{\tau } + C_r K^{\frac{1}{2}} \tau + C_r\big ( \Vert V_+\Vert _{\frac{3}{2}+\delta ,1,0} + \Vert V_-\Vert _{\frac{3}{2}+\delta ,1,0} \big ) \\&\qquad \qquad + C_r F^{\frac{1}{2}} + C_r H^{\frac{1}{2}} \Big ] \times G + C_{r}{(1+\nu +\dfrac{1}{\nu })}\Big ( K^2+ 1 \Big ) F \\&\quad + \frac{C_{r}({1+\nu })}{|\Omega |} K H + \frac{C_{r}}{|\Omega |} \Big ( {\nu \Vert \partial _z V_+\Vert _{r,1,\tau } + \nu \Vert \partial _z V_-\Vert _{r,1,\tau }} \Big ) K^{\frac{1}{2}} H^{\frac{1}{2}} \\&\quad + \frac{C_{r}{(1+\nu )}}{|\Omega |}\Big ( |\dot{\tau }|^2 + K^2 + 1 \Big ) + \dfrac{{1}}{|\Omega |} \partial _t N. \end{aligned} \end{aligned}$$

where $$ \delta \in (0,\frac{1}{2}) $$ and

5.24 $$\begin{aligned} F:=&\, \Vert A^r e^{\tau A} \overline{\phi } \Vert ^2 + \Vert \phi _+ \Vert _{r,0,\tau }^2 + \Vert \phi _- \Vert _{r,0,\tau }^2 , \end{aligned}$$

5.25 $$\begin{aligned} G :=&\, \Vert A^{r+\frac{1}{2}} e^{\tau A} \overline{\phi } \Vert ^2 + \Vert A^{r+\frac{1}{2}} e^{\tau A} \phi _+ \Vert ^2 + \Vert A^{r+\frac{1}{2}} e^{\tau A} \phi _- \Vert ^2 , \end{aligned}$$

5.26 $$\begin{aligned} H:=&\, \Vert \partial _z \phi _+ \Vert _{r,0,\tau }^2 + \Vert \partial _z \phi _- \Vert _{r,0,\tau }^2, \end{aligned}$$

5.27 $$\begin{aligned} K:=&\, \Vert {\overline{V}}\Vert _{r+2,0,\tau }^2 + \Vert V_+\Vert _{r+2,0,\tau }^2 + \Vert V_-\Vert _{r+2,0,\tau }^2 + \Vert V_+\Vert _{r+1,1,\tau }^2+ \Vert V_-\Vert _{r+1,1,\tau }^2 . \end{aligned}$$

Assume that, for the moment, we have

5.28 $$\begin{aligned} \dot{\tau } + C_r K^{\frac{1}{2}} \tau + C_r\big ( \Vert V_+\Vert _{\frac{3}{2}+\delta ,1,0} + \Vert V_-\Vert _{\frac{3}{2}+\delta ,1,0} \big ) + C_r F^{\frac{1}{2}} + C_r H^{\frac{1}{2}} = 0, \end{aligned}$$

which implies $\tau \le \tau _0$ and

$$\begin{aligned} |{\dot{\tau }}|^2 \le C_r (\tau _0^2 + 1) K + C_r (F + H). \end{aligned}$$

On the other hand, recalling *M* as in (5.1), then according to Proposition 4.2, (4.8), and (4.9), there exist $C_{M}, C_r > 1$ such that

5.29 $$\begin{aligned}&K + \int _0^t \Big (\nu \Vert \partial _z V_+ (s) \Vert _{r,1,\tau }^2 + \nu \Vert \partial _z V_-(s) \Vert _{r,1,\tau }^2 \Big ) \,ds \le \exp [ \exp [\exp (C_r t + C_{M})] ] =: {\mathcal {K}}(t) , \end{aligned}$$

5.30 $$\begin{aligned}&\qquad \text {and} \nonumber \\&\int _0^t \bigl ( \Vert V_+(s) \Vert _{\frac{3}{2}+\delta ,1,0}^2 + \Vert V_-(s) \Vert _{\frac{3}{2}+\delta ,1,0}^2 \bigr ) \,ds \le (1+\dfrac{1}{\nu }) \Vert \widetilde{V}_0 \Vert _{\frac{3}{2}+\delta , 0, 0}^2 {\mathcal {K}}(t). \end{aligned}$$

Under these conditions, from (5.23), one can derive that

5.31 $$\begin{aligned}&\frac{d}{dt} F + \dfrac{\nu }{2} H \le C_{r}(1+\nu +\dfrac{1}{\nu })\Big ( {\mathcal {K}}^2+ 1 \Big ) F + \frac{C_{r}(1+\nu )}{|\Omega |} ({\mathcal {K}} + 1 ) H + \dfrac{C_{r}}{|\Omega |^2} \Big (\nu \Vert \partial _z V_+\Vert _{r,1,\tau }^2 + \nu \Vert \partial _z V_-\Vert _{r,1,\tau }^2 \Big ) {\mathcal {K}} \nonumber \\&\quad + \frac{C_{r}(1+\nu )}{|\Omega |}\Big ( {\mathcal {K}}^2 + \tau _0^4 + 1 \Big ) + \dfrac{1}{|\Omega |} \partial _t N. \end{aligned}$$

Therefore, multiplying (5.31) with $e^{- C_{r}(1+\nu +\frac{1}{\nu })\int _0^t ({\mathcal {K}}^2 + 1)(s) \,ds}$ leads to

$$\begin{aligned}&\dfrac{d}{dt} \bigl ( F e^{- C_{r}(1+\nu +\frac{1}{\nu })\int _0^t ({\mathcal {K}}^2 + 1)(s) \,ds} \bigr ) + \bigl [ \dfrac{\nu }{2} - \frac{C_{r}(1+\nu )}{|\Omega |} ({\mathcal {K}} + 1 ) \bigr ] H e^{- C_{r}(1+\nu +\frac{1}{\nu })\int _0^t ({\mathcal {K}}^2 + 1)(s) \,ds} \\&\le \dfrac{C_{r}}{|\Omega |^2} \Big (\nu \Vert \partial _z V_+\Vert _{r,1,\tau }^2 + \nu \Vert \partial _z V_-\Vert _{r,1,\tau }^2 \Big ) {\mathcal {K}} e^{- C_{r}(1+\nu +\frac{1}{\nu })\int _0^t ({\mathcal {K}}^2 + 1)(s) \,ds} \\&\quad + \frac{C_{r}(1+\nu )}{|\Omega |}\Big ( {\mathcal {K}}^2 + \tau _0^4 + 1 \Big ) e^{- C_{r}(1+\nu +\frac{1}{\nu })\int _0^t ({\mathcal {K}}^2 + 1)(s) \,ds} + \dfrac{1}{|\Omega |} \partial _t N \times e^{- C_{r}(1+\nu +\frac{1}{\nu })\int _0^t ({\mathcal {K}}^2 + 1)(s) \,ds}. \end{aligned}$$

Integrating the above equation in time and recalling that $F(t=0)=0$, one obtains

5.32 $$\begin{aligned} \begin{aligned}&\bigl ( F(t) e^{- C_{r}(1+\nu +\frac{1}{\nu })\int _0^t ({\mathcal {K}}^2 + 1)(s) \,ds} \bigr ) + \int _0^t \bigl [ \dfrac{\nu }{2} - \frac{C_{r}(1+\nu )}{|\Omega |} ({\mathcal {K}} (t') + 1 ) \bigr ] H (t') e^{- C_{r}(1+\nu +\frac{1}{\nu })\int _0^{t'} ({\mathcal {K}}^2 + 1)(s) \,ds} \,dt' \\&\le \int _0^t \dfrac{C_{r}}{|\Omega |^2} \Big (\nu \Vert \partial _z V_+(t') \Vert _{r,1,\tau }^2 + \nu \Vert \partial _z V_-(t') \Vert _{r,1,\tau }^2 \Big ) {\mathcal {K}} e^{- C_{r}(1+\nu +\frac{1}{\nu })\int _0^{t'} ({\mathcal {K}}^2 + 1)(s) \,ds} \,dt' \\&\quad + \int _0^t \frac{C_{r}(1+\nu )}{|\Omega |}\Big ( {\mathcal {K}}^2 (t') + \tau _0^4 + 1 \Big ) e^{- C_{r}(1+\nu +\frac{1}{\nu })\int _0^{t'} ({\mathcal {K}}^2 + 1)(s) \,ds} \,dt' \\&\quad + \int _0^t \dfrac{1}{|\Omega |} \partial _t N(t') e^{- C_{r}(1+\nu +\frac{1}{\nu })\int _0^{t'} ({\mathcal {K}}^2 + 1)(s) \,ds} \,dt' \\&\le \dfrac{C_{r}}{|\Omega |^2 } {\mathcal {K}}(t) + \dfrac{C_{r}(1+\nu )}{|\Omega |} \int _0^t ( {\mathcal {K}}(t') + \tau _0^4 + 1 ) \,dt' + \dfrac{1}{|\Omega |} \int _0^t \partial _t N(t') e^{- C_{r}(1+\nu +\frac{1}{\nu })\int _0^{t'} ({\mathcal {K}}^2 + 1)(s) \,ds} \,dt', \end{aligned} \end{aligned}$$

where we have applied (5.29) and, thanks to the definition of ${\mathcal {K}}$,

5.33 $$\begin{aligned} {\mathcal {K}}(t') e^{- C_{r}(1+\nu +\frac{1}{\nu })\int _0^{t'} (\mathcal K^2 + 1)(s) \,ds} < C, \end{aligned}$$

for some constant $C \in (0,\infty )$. On the other hand, thanks to (5.14), (5.29), and (5.33), since $N(t=0)=0$, one can derive that

5.34 $$\begin{aligned} \begin{aligned}&\int _0^t \partial _t N(t') e^{- C_{r}(1+\nu +\frac{1}{\nu })\int _0^{t'} ({\mathcal {K}}^2 + 1)(s) \,ds} \,dt' = N(t) e^{- C_{r}(1+\nu +\frac{1}{\nu })\int _0^{t} ({\mathcal {K}}^2 + 1)(s) \,ds} \\&\qquad + C_{r}(1+\nu + \frac{1}{\nu }) \int _0^t N(t') ({\mathcal {K}}^2(t') + 1) e^{- C_{r}(1+\nu +\frac{1}{\nu })\int _0^{t'} ({\mathcal {K}}^2 + 1)(s) \,ds} \,dt'\\&\quad \le {\mathcal {K}}(t) e^{- C_{r}\left( 1+\nu +\frac{1}{\nu }\right) \int _0^{t} ({\mathcal {K}}^2 + 1)(s) \,ds} F^{\frac{1}{2}}(t) + C_{r}\left( 1+\nu +\dfrac{1}{\nu }\right) \\&\qquad \int _0^t ({\mathcal {K}}^2 (t') + 1 ) {\mathcal {K}}(t') F^{\frac{1}{2}}(t') e^{- C_{r}\left( 1+\nu +\frac{1}{\nu }\right) \int _0^{t'} ({\mathcal {K}}^2 + 1)(s) \,ds} \,dt' \\&\quad \le C_{r}F^{\frac{1}{2}}(t) + C_{r}(1+\nu + \frac{1}{\nu }) \int _0^t ({\mathcal {K}}^2 (t') + 1 )F^{\frac{1}{2}}(t') \,dt'. \end{aligned} \end{aligned}$$

Hence, (5.32) implies that, for $t \in [0,{\mathcal {T}}]$, since $|\Omega |>1$, after applying the young inequality,

5.35 $$\begin{aligned} \begin{aligned}&F(t) + \dfrac{\nu }{4}\int _0^t H (t') \,dt' \le \dfrac{C_{r}}{|\Omega |} {\mathcal {K}}(t) e^{C_{r}(1+\nu +\frac{1}{\nu })\int _0^t ({\mathcal {K}}^2 + 1)(s) \,ds} \\&\qquad + \dfrac{C_{r}(1+\nu +\frac{1}{\nu })^2}{|\Omega |} \biggl ( \int _0^t \bigl ( {\mathcal {K}}^2(t') + 1 \bigr ) \,dt'\biggr )^2 e^{C_{r}(1+\nu +\frac{1}{\nu })\int _0^t ({\mathcal {K}}^2 + 1)(s) \,ds} \\&\qquad + \dfrac{C_{r}(1+\nu )}{|\Omega |} \int _0^t \bigl ( {\mathcal {K}}(t') + \tau _0^4 + 1 \bigr ) \,dt' \times e^{C_{r}(1+\nu +\frac{1}{\nu })\int _0^t ({\mathcal {K}}^2 + 1)(s) \,ds}, \end{aligned} \end{aligned}$$

where ${\mathcal {T}} \in (0,\infty ]$ is given by the following constraints:

5.36 $$\begin{aligned} \tau (s)> 0 \qquad \text {and} \qquad \dfrac{\nu }{2} - \dfrac{C_{r}(1+\nu )}{|\Omega |}({\mathcal {K}}(s) + 1 ) \ge \dfrac{\nu }{4} > 0 \qquad \text {for} \quad s \in [0,{\mathcal {T}}]. \end{aligned}$$

Since $|\Omega |\ge |\Omega _0|$, in particular, there exists a constant ${\mathcal {C}}_{M,r,\tau _0} \in (1,\infty )$ such that, for $t \in (0,{\mathcal {T}}]$,

5.37 $$\begin{aligned} F(t) + \dfrac{\nu }{4}\int _0^t H(t') \,dt' \le \dfrac{1}{|\Omega _0|}(1+\nu +\dfrac{1}{\nu })^2 \exp \biggl [(1+\nu +\frac{1}{\nu })\exp [\exp [\exp [{\mathcal {C}}_{M,r,\tau _0}(t+1)]]]\biggr ] . \end{aligned}$$

Now we will be able to estimate ${\mathcal {T}}$. To ensure $\tau > 0$ in (5.36), from (5.28), (5.29), (5.30), and (5.37), one has

5.38 $$\begin{aligned} \begin{aligned}&\tau (t) = - C_r \int _0^t e^{-C_r \int _{t'}^t K^{\frac{1}{2}}(s)\,ds} \bigl (\Vert V_+\Vert _{3/2+\delta , 1,0} +\Vert V_-\Vert _{3/2+\delta , 1,0} + F^{\frac{1}{2}} + H^{\frac{1}{2}} \bigr ) \,dt'\\&\qquad + \tau _0 e^{-C_r \int _0^t K^{\frac{1}{2}}(t')\,dt'} \ge \tau _0 \exp [\exp [\exp [\exp [-{\mathcal {C}}'_{M,r}(t+1)]]]] \\&\qquad \qquad - C_r \biggl ((1+\dfrac{1}{\nu ^{1/2}})\Vert {\widetilde{V}}_0 \Vert _{\frac{3}{2}+\delta , 0,0} + \dfrac{1}{|\Omega _0|^{1/2}} (1 + \nu + \dfrac{1}{\nu ^{3/2}}) \biggr )\\&\qquad \qquad \exp \biggl [(1+\nu +\frac{1}{\nu })\exp [\exp [\exp [C_{M,r,\tau _0}(t+1)]]] \biggr ] \end{aligned} \end{aligned}$$

for some constant ${\mathcal {C}}'_{M,r}, {\mathcal {C}}'_{M,r,\tau _0} \in (1,\infty )$. Notably, the function $\tau (t)$ we obtain is bounded above by (4.15). Therefore, for $t > 0$ satisfying

5.39 $$\begin{aligned} \begin{aligned} \exp \biggl [(1+\nu +\frac{1}{\nu })\exp [\exp [\exp [ {\mathcal {C}}'_{M,r,\tau _0}(t+1)]]] -\exp [\exp [\exp [-C_{M,r}'(t+1)]]] \biggr ]\\ < \dfrac{\tau _0}{2 C_r \bigl ((1+\dfrac{1}{\nu ^{1/2}})\Vert {\widetilde{V}}_0 \Vert _{\frac{3}{2}+\delta , 0,0} + \frac{1}{|\Omega _0|^{1/2}} (1 + \nu + \dfrac{1}{\nu ^{3/2}}) \bigr )}, \end{aligned} \end{aligned}$$

or equivalently, under the assumption of (5.2), for some $C_{M,r,\tau _0}'' \in (1,\infty )$,

5.40 $$\begin{aligned}&\exp \biggl [ (1+\nu +\frac{1}{\nu })\exp [\exp [\exp [ {\mathcal {C}}''_{M,r,\tau _0}(t+1)]]] -\exp [\exp [\exp [-C_{M,r,\tau _0}''(t+1)]]] \biggr ] \nonumber \\&\quad < \dfrac{C_{M,r,\tau _0}''|\Omega _0|^{1/2}}{1+\nu + \frac{1}{\nu ^{3/2}}}, \end{aligned}$$

it follows that $\tau (t) > 0$. In particular, for $t \in (0,T]$ with

5.41 $$\begin{aligned} T = \dfrac{1}{C''_{M,r,\tau _0}}\log \left[ \frac{1}{e^{C''_{M,r,\tau _0}}}\log \left[ \log \left[ \dfrac{\log \left( \frac{C_{M,r,\tau _0}''|\Omega _0|^{1/2}}{1+\nu + \frac{1}{\nu ^{3/2}}}\right) }{1+\nu +\frac{1}{\nu }}\right] \right] \right] , \end{aligned}$$

the above inequality is satisfied.

Consequently, under condition (5.2), (5.36) and (5.39) imply (5.3), and (5.37) implies (5.4) thanks to (5.5). This completes the proof of Theorem 5.1.

### Proof of Theorem 5.2

In this section, we prove Theorem 5.2. We only sketch the proof for the first two parts, and will provide detailed proof for the third part.

For the first part of the theorem, thanks to Remark 8, we know that when $\sup _{0\le t< \infty }\Vert \overline{V}(t)\Vert _{r+3,0,\tau (t)} \le C_{M,r}$ the growth of $\Vert \widetilde{V}(t)\Vert _{r+2,1,\tau (t)}$ will only be exponentially in time. Thus, the function ${\mathcal {K}}(t)$ appears in the proof of Theorem 5.1 (e.g., (5.29) and (5.35)) becomes only exponentially in time. This reduces two logarithms in the estimate of existence time and gives

$$\begin{aligned} {\mathcal {T}} = \frac{1}{C_{\tau _0, M, r, \nu } } \log (\log (|\Omega _0|) ). \end{aligned}$$

This can be seen as in (5.36) – (5.39).

Similarly, for the second part of Theorem 5.2, thanks to Remark 8, when $\sup _{0\le t < \infty }\Vert \overline{V}(t)\Vert _{r+3,0,\tau } \le \frac{\nu }{4C_{r,\alpha }}$ is small enough $\Vert \widetilde{V}(t)\Vert _{r+2,1,\tau (t)}$ does not grow and thus the function ${\mathcal {K}}(t)$ is uniformly-in-time bounded. This reduces one more logarithm and gives

$$\begin{aligned} {\mathcal {T}} = \frac{1}{C_{\tau _0, M, r, \nu } } \log (|\Omega _0|) ). \end{aligned}$$

To show that the smallness condition (5.2) can be relaxed, recalling *K* in (5.27). Under our new assumption on $\overline{{\mathcal {V}}}$, thanks to Remark 8, we have that $K^{\frac{1}{2}} \le \frac{\nu }{C_{r,\alpha }} + C_M e^{-\frac{\nu }{2}t}$ and $\Vert V_+(t)\Vert _{3/2+\delta , 1,0} +\Vert V_-(t)\Vert _{3/2+\delta , 1,0} \le \frac{\tau _0}{C_{r,\nu ,M}} e^{-\frac{\nu }{2}t}$. Now recall from (5.38) that

$$\begin{aligned} \begin{aligned} \tau (t) = \Big (\tau _0 - C_r \int _0^t e^{C_r \int _0^{t'} K^{\frac{1}{2}}(s)\,ds} \bigl (\Vert V_+\Vert _{3/2+\delta , 1,0} +\Vert V_- \Vert _{3/2+\delta , 1,0} + F^{\frac{1}{2}} + H^{\frac{1}{2}} \bigr ) \,dt' \Big ) e^{-C_r \int _0^t K^{\frac{1}{2}}(t')\,dt'}, \end{aligned} \end{aligned}$$

in which we will ask for

$$\begin{aligned} \tau _0 - C_r \int _0^t e^{C_r \int _0^{t'} K^{\frac{1}{2}}(s)\,ds} \bigl (\Vert V_+\Vert _{3/2+\delta , 1,0} +\Vert V_-\Vert _{3/2+\delta , 1,0}\bigr ) dt' \\ \ge \tau _0 - C_r \int _0^\infty C_{M,\nu } \frac{\tau _0}{C_{r,\nu ,M}} e^{\frac{C_r}{C_{r,\alpha }} \nu t' - \frac{\nu }{2} t'} dt' \ge \frac{\tau _0}{2}, \end{aligned}$$

provided that $C_{r,\nu ,M}$ and $C_{r,\alpha }$ are large enough. From this, one can conclude that the smallness assumption can be relaxed and replaced by $\Vert \widetilde{\mathcal {V}}_0\Vert _{\frac{3}{2}+\delta ,0,0} \le \frac{\tau _0}{C_{r,\nu ,M}}$.

Next we give the detailed proof to the third part of Theorem 5.2. Consider the initial data satisfying $\Vert \overline{{\mathcal {V}}}_0\Vert _{r+3,0,\tau _0} \le \frac{M}{|\Omega |_0}$. We set $\overline{V} = 0$ and replace the initial condition (5.8) of the perturbed system to

$$\begin{aligned} \overline{\phi }_0 = \overline{\mathcal {V}}_0, \quad (\phi _\pm )_0 = 0. \end{aligned}$$

With more careful estimates, (5.23) becomes

5.42 $$\begin{aligned} \begin{aligned}&\frac{d}{dt} F + \nu H \le \Big [ \dot{\tau } + C_r K^{\frac{1}{2}} \tau + C_r \big ( \Vert V_+\Vert _{\frac{3}{2}+\delta ,1,0} + \Vert V_-\Vert _{\frac{3}{2}+\delta ,1,0} \big ) \\&\qquad \qquad + C_r F^{\frac{1}{2}} + C_r H^{\frac{1}{2}} \Big ] \times G + C_{r,\nu }L F \\&\quad + \frac{C_{r,\nu }}{|\Omega |} K H + \frac{C_{r,\nu }}{|\Omega |}\Big (\Vert \partial _z V_+\Vert _{r,1,\tau } + \Vert \partial _z V_-\Vert _{r,1,\tau } \Big ) K^{\frac{1}{2}} H^{\frac{1}{2}} \\&\quad + \frac{C_{r,\nu ,\tau _0}}{|\Omega |} L + \dfrac{C_{r, \nu }}{|\Omega |} \partial _t N, \end{aligned} \end{aligned}$$

where $\delta \in (0,\frac{1}{2})$ and *F*, *G*, *H* are defined as in (5.24)–(5.26),

$$\begin{aligned} K:= \Vert V_+\Vert _{r+2,0,\tau }^2 + \Vert V_-\Vert _{r+2,0,\tau }^2 + \Vert V_+ \Vert _{r+1,1,\tau }^2+ \Vert V_-\Vert _{r+1,1,\tau }^2, \quad L:= K^{\frac{1}{2}} + K + K^2, \end{aligned}$$

and

5.43 $$\begin{aligned} \dot{\tau } + C_r K^{\frac{1}{2}} \tau + C_r\big ( \Vert V_+ \Vert _{\frac{3}{2}+\delta ,1,0} + \Vert V_-\Vert _{\frac{3}{2}+\delta ,1,0} \big ) + C_r F^{\frac{1}{2}} + C_r H^{\frac{1}{2}}=0. \end{aligned}$$

On the other hand, thanks to Remark 8, (4.8), and (4.9), there exist $C_{M,\nu }, C_r, C > 1$ such that

5.44 $$\begin{aligned}&L \le C_{M} e^{-\frac{\nu }{C}t} =: {\mathcal {K}}(t) , \end{aligned}$$

5.45 $$\begin{aligned}&\nu \int _0^t \Big (\Vert \partial _z V_+ (s) \Vert _{r,1,\tau }^2 + \Vert \partial _z V_-(s) \Vert _{r,1,\tau }^2 \Big ) e^{\nu s}\,ds \le C_M \qquad \text {and} \end{aligned}$$

5.46 $$\begin{aligned}&\nu \int _0^t \bigl ( \Vert V_+(s) \Vert _{\frac{3}{2}+\delta ,1,0}^2 + \Vert V_-(s) \Vert _{\frac{3}{2}+\delta ,1,0}^2 \bigr ) e^{\nu s} \,ds \le C\Vert \widetilde{V}_0 \Vert _{\frac{3}{2}+\delta , 0, 0}^2 . \end{aligned}$$

With these conditions, from (5.42), one can derive that

$$\begin{aligned} \begin{aligned}&\frac{d}{dt} F + \nu H \le C_{r,\nu } L F + \frac{C_{r,\nu }}{|\Omega |} ({\mathcal {K}} + 1 ) H + \dfrac{C_{r,\nu }}{|\Omega |^2} \Big (\Vert \partial _z V_+\Vert _{r,1,\tau }^2 + \Vert \partial _z V_-\Vert _{r,1,\tau }^2 \Big ) {\mathcal {K}} + \frac{C_{r,\nu ,\tau _0}}{|\Omega |}L + \dfrac{C_{r, \nu }}{|\Omega |} \partial _t N, \end{aligned} \end{aligned}$$

and thus

5.47 $$\begin{aligned} \begin{aligned}&\frac{d}{dt} F + \frac{\nu }{2} H \le C_{r,\nu } L F + \dfrac{C_{r,\nu }}{|\Omega |^2} \Big (\Vert \partial _z V_+\Vert _{r,1,\tau }^2 + \Vert \partial _z V_-\Vert _{r,1,\tau }^2 \Big ) {\mathcal {K}} + \frac{C_{r,\nu ,\tau _0}}{|\Omega |}L + \dfrac{C_{r, \nu }}{|\Omega |} \partial _t N, \end{aligned} \end{aligned}$$

provided that $|\Omega | > C_{M,r,\nu }$ for some positive constant $C_{M,r,\nu } >0$. Multiplying (5.47) with $e^{- C_{r,\nu }\int _0^t L(s) \,ds}$ leads to

$$\begin{aligned}&\dfrac{d}{dt} \bigl ( F e^{- C_{r,\nu }\int _0^t L(s) \,ds} \bigr ) + \dfrac{\nu }{2} H e^{- C_{r,\nu }\int _0^t L(s) \,ds} \le \dfrac{C_{r,\nu }}{|\Omega |^2} \Big (\Vert \partial _z V_+\Vert _{r,1,\tau }^2 + \Vert \partial _z V_-\Vert _{r,1,\tau }^2 \Big ) {\mathcal {K}} e^{- C_{r,\nu }\int _0^t L(s) \,ds}\\&\qquad \qquad \qquad + \frac{C_{r,\nu ,\tau _0}}{|\Omega |}L e^{- C_{r,\nu }\int _0^t L(s) \,ds} + \dfrac{C_{r, \nu }}{|\Omega |} \partial _t N e^{- C_{r,\nu }\int _0^t L(s) \,ds}. \end{aligned}$$

After integrating the above equation in time and recalling that $F(t=0)\le \frac{M}{|\Omega _0|}$, since $|\Omega |>|\Omega _0|>1$, one obtains

5.48 $$\begin{aligned} \begin{aligned}&\bigl ( F(t) e^{- C_{r,\nu }\int _0^t L(s) \,ds} \bigr ) + \int _0^t \dfrac{\nu }{2} H (t') e^{- C_{r,\nu }\int _0^{t'} L(s) \,ds} \,dt' \\&\quad \le \frac{C_M}{|\Omega _0|} + \int _0^t \dfrac{C_{r,\nu }}{|\Omega _0|} \Big (\Vert \partial _z V_+(t') \Vert _{r,1,\tau }^2 + \Vert \partial _z V_-(t') \Vert _{r,1,\tau }^2 \Big ) {\mathcal {K}} e^{- C_{r,\nu }\int _0^{t'} L(s) \,ds} \,dt' \\&\qquad + \int _0^t \frac{C_{r,\nu ,\tau _0}}{|\Omega _0|}L(t') e^{- C_{r,\nu }\int _0^{t'} L(s) \,ds} \,dt' + \int _0^t \dfrac{C_{r, \nu }}{|\Omega _0|} \partial _t N(t') e^{- C_{r,\nu }\int _0^{t'} L(s) \,ds} \,dt' \\&\quad \le \dfrac{C_{M,r,\nu ,\tau _0}}{|\Omega _0| } + \dfrac{C_{r, \nu }}{|\Omega _0|} \int _0^t \partial _t N(t') e^{- C_{r,\nu }\int _0^{t'} L(s) \,ds} \,dt'. \end{aligned} \end{aligned}$$

According to (5.34), since now $N(0)\ne 0$ due to $\overline{\phi }_0 \ne 0$, the estimate becomes

$$\begin{aligned} \begin{aligned}&\int _0^t \partial _t N(t') e^{- C_{r,\nu }\int _0^{t'} L(s) \,ds} \,dt' = N(t) e^{- C_{r,\nu }\int _0^{t} ({\mathcal {K}}^2 + 1)(s) \,ds} -N(0)\\&\qquad + C_{r,\nu } \int _0^t N(t') L(t') e^{- C_{r,\nu }\int _0^{t'} L(s) \,ds} \,dt'\\&\quad \le C_{M,r,\nu } \Big (F^{\frac{1}{2}}(t)+1 \Big ) + C_{r,\nu } \int _0^t {\mathcal {K}}(t') F^{\frac{1}{2}}(t') \,dt' . \end{aligned} \end{aligned}$$

Hence, (5.48) implies that, for $t \in [0,{\mathcal {T}}]$, after applying the young inequality, one has

5.49 $$\begin{aligned} \begin{aligned}&F(t) + \int _0^t H (t') \,dt' \le \dfrac{C_{M,r,\nu ,\tau _0}}{|\Omega _0|} , \end{aligned} \end{aligned}$$

where ${\mathcal {T}} \in (0,\infty ]$ is given by the constraint

$$\begin{aligned} \tau (s) > 0 \qquad \text {for} \quad s \in [0,{\mathcal {T}}]. \end{aligned}$$

Now we will be able to estimate ${\mathcal {T}}$. To ensure $\tau > 0$, from (5.43), (5.44), (5.46), and (5.49), one has

5.50 $$\begin{aligned} \begin{aligned} \tau (t)&= - C_r \int _0^t e^{-C_r \int _{t'}^t K^{\frac{1}{2}}(s)\,ds} \bigl (\Vert V_+\Vert _{3/2+\delta , 1,0} +\Vert V_-\Vert _{3/2+\delta , 1,0} + F^{\frac{1}{2}} + H^{\frac{1}{2}} \bigr ) \,dt'\\&\qquad \qquad \qquad + \tau _0 e^{-C_r \int _0^t K^{\frac{1}{2}}(t')\,dt'}\\&\ge \tau _0 C'_{M,r,\nu } - C'_{M,r,\nu ,\tau _0} \dfrac{1}{|\Omega _0|^{\frac{1}{2}}} (t+1) - C_{r,\nu } \Vert {\widetilde{V}}_0 \Vert _{\frac{3}{2}+\delta , 0,0} \end{aligned} \end{aligned}$$

for some constant ${\mathcal {C}}'_{M,r,\nu } \in (0,1), C_{r,\nu }, {\mathcal {C}}'_{M,r,\nu , \tau _0} \in (1,\infty )$. Therefore, for $t > 0$ satisfying

5.51 $$\begin{aligned} t+1 < \dfrac{C'_{M,r,\nu } \tau _0 |\Omega _0|^{\frac{1}{2}}}{2{\mathcal {C}}'_{M,r,\nu , \tau _0} } \end{aligned}$$

and $$\Vert {\widetilde{V}}_0 \Vert _{\frac{3}{2}+\delta , 0,0}$$ satisfying

$$\begin{aligned} \Vert {\widetilde{V}}_0 \Vert _{\frac{3}{2}+\delta , 0,0} < \frac{\tau _0 C'_{M,r,\nu }}{2C_{r,\nu }}, \end{aligned}$$

it follows that $\tau (t) > 0$. Consequently, (5.51) implies ${\mathcal {T}} = \frac{|\Omega _0|^{\frac{1}{2}}}{C_{\tau _0, M, r, \nu } }$. This completes the proof of Theorem 5.2.

## Global Existence in 2*D* with $\Omega =0$

In this section, we show that the weak solution obtained in section 3 exists globally in time in the case of 2*D* and $\Omega =0$, provided that the initial data is small. This result is similar to the one in [46], where system (1.1) with Dirichlet boundary condition is considered.

To be more precious, let us consider $\mathcal {V}=(u,v)^\top (x,z,t)$ with (2.7), i.e., the solution to system (1.1) independent of the *y*-variable. It is easy to verify that

6.1a $$\begin{aligned}&\overline{u}=0, \end{aligned}$$

6.1b $$\begin{aligned}&\partial _t \overline{v} + \partial _x {\mathfrak {P}}_0 (\widetilde{u} \widetilde{v})=0, \end{aligned}$$

6.1c $$\begin{aligned}&\partial _t \widetilde{u} + \widetilde{u}\partial _x \widetilde{u} - \partial _x {\mathfrak {P}}_0 (\widetilde{u}^2) - \Big (\int _0^z \partial _x\widetilde{u}(x,s) ds \Big ) \partial _z \widetilde{u} - \Omega \widetilde{v} -\nu \partial _{zz} \widetilde{u} = 0, \end{aligned}$$

6.1d $$\begin{aligned}&\partial _t \widetilde{v} + \widetilde{u}\partial _x \widetilde{v} + \widetilde{u}\partial _x \overline{v} - \partial _x {\mathfrak {P}}_0 (\widetilde{u} \widetilde{v}) - \Big (\int _0^z \partial _x\widetilde{u}(x,s) ds \Big ) \partial _z \widetilde{v} + \Omega \widetilde{u} -\nu \partial _{zz} \widetilde{v}=0. \end{aligned}$$

We remind readers that ${\mathfrak {P}}_0$ is the barotropic projection operator defined in (2.14). In addition, let $\Omega = 0$. Then one can observe that $\overline{v}\equiv 0$ and $\widetilde{v}\equiv 0$ are invariant in time, a property that is not true in the case of $\Omega \ne 0$. Consequently, with $\Omega = 0$ and $\overline{v}_0 = \widetilde{v}_0 = 0$, system (6.1) reduces to

6.2 $$\begin{aligned} \partial _t \widetilde{u} + \widetilde{u}\partial _x \widetilde{u} - \partial _x {\mathfrak {P}}_0 (\widetilde{u}^2) - \Big (\int _0^z \partial _x\widetilde{u}(x,s) ds \Big ) \partial _z \widetilde{u} -\nu \partial _{zz} \widetilde{u} = 0 \qquad \text {with} \qquad \partial _z \widetilde{u}|_{z=0,1}. \end{aligned}$$

We have the following theorem concerning the global existence of the weak solutions to (6.2) with $\Omega = 0$:

### Theorem 6.1

For $r>2$ and $\tau _0 > 0$, suppose that the initial data ${\widetilde{u}}|_{t=0} = \widetilde{u}_0 \in \mathcal {S}_{r,0,\tau _0}$ with $\int _0^1 \widetilde{u}_0(x,z) \,dz = 0$ satisfies the smallness condition

6.3 $$\begin{aligned} \Vert \widetilde{u}_0 \Vert _{r,0,\tau _0} < \frac{\nu \tau _0}{{\mathcal {C}}_r} , \end{aligned}$$

where ${\mathcal {C}}_r > 0$ is a constant as in (6.5), below. Then the unique weak solution to system (6.2) exists globally in time.

### Proof

(Sketch of proof) Similarly to (3.1), we have

$$\begin{aligned} \begin{aligned}&\frac{1}{2} \frac{d}{dt} \Vert \widetilde{u} \Vert _{r,0,\tau }^2 + \nu \Vert \partial _z \widetilde{u} \Vert _{r,0,\tau }^2= \dot{\tau } \Vert A^{r+\frac{1}{2}} e^{\tau A} \widetilde{u}\Vert ^2 - \Big \langle A^r e^{\tau A}\widetilde{u}\partial _x \widetilde{u} , A^r e^{\tau A} \widetilde{u} \Big \rangle \\&\qquad \qquad - \Big \langle A^r e^{\tau A} \Big (\int _0^z \partial _x\widetilde{u}(x,s) ds \Big ) \partial _z \widetilde{u} , A^r e^{\tau A} \widetilde{u} \Big \rangle \\&\qquad \le \Big (\dot{\tau } + C_r(\Vert \widetilde{u} \Vert _{r,0,\tau } + \Vert \partial _z \widetilde{u} \Vert _{r,0,\tau }) \Big ) \Vert A^{r+\frac{1}{2}} e^{\tau A} \widetilde{u}\Vert ^2, \end{aligned} \end{aligned}$$

thanks to Lemma A.1 and Lemma A.2.

It is easy to see that $\int _0^1 \widetilde{u}(x,z) dz = 0$. One can apply the Poincaré inequality to get $\Vert \widetilde{u} \Vert _{r,0,\tau } \le \Vert \partial _z \widetilde{u} \Vert _{r,0,\tau },$ and consequently,

$$\begin{aligned} \frac{1}{2} \frac{d}{dt} \Vert \widetilde{u} \Vert _{r,0,\tau }^2 + \frac{\nu }{2} \Vert \partial _z \widetilde{u} \Vert _{r,0,\tau }^2 \le \Big (\dot{\tau } + C_r \Vert \partial _z \widetilde{u} \Vert _{r,0,\tau } \Big ) \Vert A^{r+\frac{1}{2}} e^{\tau A} \widetilde{u}\Vert ^2 - \frac{\nu }{2}\Vert \widetilde{u} \Vert _{r,0,\tau }^2. \end{aligned}$$

Assuming that

6.4 $$\begin{aligned} \dot{\tau } + C_r \Vert \partial _z \widetilde{u} \Vert _{r,0,\tau } = 0, \end{aligned}$$

one has

$$\begin{aligned} \frac{d}{dt} \Vert \widetilde{u} \Vert _{r,0,\tau }^2 + \nu \Vert \partial _z \widetilde{u} \Vert _{r,0,\tau }^2 \le -\nu \Vert \widetilde{u} \Vert _{r,0,\tau }^2. \end{aligned}$$

After applying the Grönwall inequality, one obtains

$$\begin{aligned} \Vert \widetilde{u}(t) \Vert _{r,0,\tau (t)}^2 e^{\nu t} + \nu \int _0^t \Vert \partial _z \widetilde{u}(s) \Vert _{r,0,\tau (s)}^2 e^{\nu s} ds \le \Vert \widetilde{u}_0 \Vert _{r,0,\tau _0}^2 . \end{aligned}$$

Therefore, integrating (6.4) from 0 to $t\in (0,\infty )$ and applying the Hölder inequality in the resultant lead to

6.5 $$\begin{aligned} \begin{aligned} \tau (t)&= \tau _0 - C_r \int _0^t \Vert \partial _z \widetilde{u}(s) \Vert _{r,0,\tau (s)} ds \\&\ge \tau _0 -C_r \Big ( \int _0^t \Vert \partial _z \widetilde{u}(s) \Vert _{r,0,\tau (s)}^2 e^{\nu s} ds\Big )^{\frac{1}{2}} \Big ( \int _0^t e^{-\nu s} ds\Big )^{\frac{1}{2}} \\&\ge \tau _0 - \frac{{\mathcal {C}}_r}{\nu } \Vert \widetilde{u}_0 \Vert _{r,0,\tau _0}, \end{aligned} \end{aligned}$$

for some positive constant ${\mathcal {C}}_r \in (0,\infty )$.

In summary, for the initial data satisfying (6.3), we have that $\tau (t) > 0$ for all $t>0$, and thus the solution exists for all time. $\square$

## Data Availability

Our manuscript has no associated data.

## Appendix A. Estimates of Nonlinear Terms

In this appendix, we list the estimates of nonlinear terms in the analytic-Sobolev spaces $\mathcal {S}_{r,s,\tau }$. Lemma A.1–A.2 will be used to prove the local well-posedness, and similar estimates can be found in [23, 28].

### Lemma A.1

For $f, g, h\in \mathcal {S}_{r+\frac{1}{2},s,\tau }$, where $r>1$, $s\ge 0$, and $\tau \ge 0$, one has

A.1 $$\begin{aligned} \begin{aligned}&\Big |\Big \langle A^r e^{\tau A} (f\cdot \nabla g), A^r e^{\tau A} h \Big \rangle \Big | \\ \le&\int _0^1 C_r\Big [ (\Vert A^r e^{\tau A} f(z)\Vert _{L^2(\mathbb {T}^2)} + |\hat{f}_0(z)| ) \Vert A^{r+\frac{1}{2}} e^{\tau A} g(z)\Vert _{L^2(\mathbb {T}^2)} \Vert A^{r+\frac{1}{2}} e^{\tau A} h(z)\Vert _{L^2(\mathbb {T}^2)} \\&+ \Vert A^{r+\frac{1}{2}} e^{\tau A} f(z)\Vert _{L^2(\mathbb {T}^2)} \Vert A^{r+\frac{1}{2}} e^{\tau A} g(z)\Vert _{L^2(\mathbb {T}^2)} \Vert A^{r} e^{\tau A} h(z)\Vert _{L^2(\mathbb {T}^2)}\Big ] dz. \end{aligned} \end{aligned}$$

### Proof

First, notice that $\Big |\Big \langle A^r e^{\tau A} (f\cdot \nabla g), A^r e^{\tau A} h \Big \rangle \Big | = \Big |\Big \langle f\cdot \nabla g, A^r e^{\tau A} H \Big \rangle \Big |$, where $H = A^r e^{\tau A} h$. Using the Fourier representation, we have,

A.2a $$\begin{aligned} f(\varvec{x},z)&= \sum \limits _{\varvec{j}\in 2\pi \mathbb {Z}^2} \hat{f}_{\varvec{j}}(z) e^{ i\varvec{j}\cdot \varvec{x}}, \end{aligned}$$

A.2b $$\begin{aligned} g(\varvec{x},z)&= \sum \limits _{\varvec{k}\in 2\pi \mathbb {Z}^2} \hat{g}_{\varvec{k}}(z) e^{ i\varvec{k}\cdot \varvec{x}}, \end{aligned}$$

A.2c $$\begin{aligned} h(\varvec{x},z)&= \sum \limits _{\varvec{l}\in 2\pi \mathbb {Z}^2} \hat{h}_{\varvec{l}}(z) e^{ i\varvec{l}\cdot \varvec{x}}, \qquad \text {and by definition}, \end{aligned}$$

A.2d $$\begin{aligned} A^r e^{\tau A} H(\varvec{x},z)&= \sum \limits _{\varvec{l}\in 2 \pi \mathbb {Z}^2} |\varvec{l}|^r e^{\tau |\varvec{l}|}\hat{H}_{\varvec{l}}(z) e^{i\varvec{l}\cdot \varvec{x}}, \qquad \text {with} \quad {\hat{H}}_{\varvec{l}}(z) = | \varvec{l} |^r e^{\tau |\varvec{l}|} {\hat{h}}_{\varvec{l}}(z). \end{aligned}$$

Therefore,

$$\begin{aligned} \Big |\Big \langle f\cdot \nabla g, A^r e^{\tau A} H \Big \rangle \Big | \le \int _0^1 \sum \limits _{\varvec{j}+\varvec{k}+\varvec{l}=0} |\hat{f}_{\varvec{j}}(z)||\varvec{k}||\hat{g}_{\varvec{k}}(z)||\varvec{l}|^r e^{\tau |\varvec{l}|} |\hat{H}_{\varvec{l}}(z)| dz. \end{aligned}$$

Since $|\varvec{l}| = |\varvec{j}+\varvec{k}| \le |\varvec{j}|+|\varvec{k}|$, we have the following inequalities:

$$\begin{aligned} |\varvec{l}|^r \le (|\varvec{j}|+|\varvec{k}|)^r \le C_r(|\varvec{j}|^r + |\varvec{k}|^r), \;\;\; e^{\tau |\varvec{l}|} \le e^{\tau |\varvec{j}|} e^{\tau |\varvec{k}|}. \end{aligned}$$

Applying these inequalities, we have

$$\begin{aligned} \Big |\Big \langle f\cdot \nabla g, A^r e^{\tau A} H \Big \rangle \Big | \le \int _0^1 \sum \limits _{\varvec{j}+\varvec{k}+\varvec{l}=0} C_r|\hat{f}_{\varvec{j}}(z)||\varvec{k}||\hat{g}_{\varvec{k}}(z)|(|\varvec{j}|^r+|\varvec{k}|^r)e^{\tau |\varvec{j}|}e^{\tau |\varvec{k}|}|\varvec{l}|^r e^{\tau |\varvec{l}|}|\hat{h}_{\varvec{l}}(z)| dz. \end{aligned}$$

Since $|\varvec{k}|, |\varvec{j}|, |\varvec{l}| \ge 0$, we have $|\varvec{k}|^{\frac{1}{2}} \le (|\varvec{j}|+|\varvec{l}|)^{\frac{1}{2}} \le |\varvec{j}|^{\frac{1}{2}} + |\varvec{l}|^{\frac{1}{2}}$, therefore,

$$\begin{aligned} \begin{aligned}&\Big |\Big \langle f\cdot \nabla g, A^r e^{\tau A} H \Big \rangle \Big | \\ \le&\int _0^1 \sum \limits _{\varvec{j}+\varvec{k}+\varvec{l}=0} C_r|\hat{f}_{\varvec{j}}(z)||\varvec{k}|^{\frac{1}{2}}(|\varvec{j}|^{\frac{1}{2}} + |\varvec{l}|^{\frac{1}{2}})|\hat{g}_{\varvec{k}}(z)|(|\varvec{j}|^r+|\varvec{k}|^r)e^{\tau |\varvec{j}|}e^{\tau |\varvec{k}|}|\varvec{l}|^r e^{\tau |\varvec{l}|}|\hat{h}_{\varvec{l}}(z)| dz \\ \le&\int _0^1 \sum \limits _{\varvec{j}+\varvec{k}+\varvec{l}=0} C_r \Big (|\varvec{k}|^{\frac{1}{2}}|\varvec{j}|^{r+\frac{1}{2}} |\varvec{l}|^{r} + |\varvec{k}|^{r+\frac{1}{2}}|\varvec{j}|^{\frac{1}{2}} |\varvec{l}|^{r} + |\varvec{k}|^{\frac{1}{2}}|\varvec{j}|^{r} |\varvec{l}|^{r+\frac{1}{2}} + |\varvec{k}|^{r+\frac{1}{2}}|\varvec{l}|^{r+\frac{1}{2}} \Big ) \\&\qquad \times e^{\tau |\varvec{j}|}e^{\tau |\varvec{k}|} e^{\tau |\varvec{l}|} |\hat{f}_{\varvec{j}}(z)| |\hat{g}_{\varvec{k}}(z)| |\hat{h}_{\varvec{l}}(z)| dz =: \int _0^1 (A_1 + A_2 + A_3 + A_4)(z) dz. \end{aligned} \end{aligned}$$

Thanks to Cauchy-Schwarz inequality, since $r>1$, we have

$$\begin{aligned} \begin{aligned} A_1&= \sum \limits _{\varvec{j}+\varvec{k}+\varvec{l}=0} C_r |\varvec{k}|^{\frac{1}{2}}|\varvec{j}|^{r+\frac{1}{2}} |\varvec{l}|^{r} e^{\tau |\varvec{j}|}e^{\tau |\varvec{k}|} e^{\tau |\varvec{l}|} |\hat{f}_{\varvec{j}}(z)| |\hat{g}_{\varvec{k}}(z)| |\hat{h}_{\varvec{l}}(z)| \\&= C_r \sum \limits _{\begin{array}{c} \varvec{k}\in 2\pi \mathbb {Z}^2 \\ \varvec{k}\ne 0 \end{array} } \biggl [|\varvec{k}|^{\frac{1}{2}} |\hat{g}_{\varvec{k}}(z)| e^{\tau |\varvec{k}|} \sum \limits _{\begin{array}{c} \varvec{j}\in 2\pi \mathbb {Z}^2 \\ \varvec{j}\ne 0, -\varvec{k} \end{array} } |\varvec{j}|^{r+\frac{1}{2}} e^{\tau |\varvec{j}|}|\hat{f}_{\varvec{j}}(z)| |\varvec{j}+\varvec{k}|^{r}e^{\tau |\varvec{j}+\varvec{k}|}|\hat{h}_{-\varvec{j}-\varvec{k}}(z)| \biggr ]\\&\le C_r \Big ( \sum \limits _{\begin{array}{c} \varvec{k}\in 2\pi \mathbb {Z}^2 \\ \varvec{k}\ne 0 \end{array} } |\varvec{k}|^{-2r}\Big )^{\frac{1}{2}} \Big ( \sum \limits _{\begin{array}{c} \varvec{k}\in 2\pi \mathbb {Z}^2 \\ \varvec{k}\ne 0 \end{array} } |\varvec{k}|^{2r+1} e^{2\tau |\varvec{k}|} |\hat{g}_{\varvec{k}}(z)|^2\Big )^{\frac{1}{2}} \\&\qquad \times \sup \limits _{\varvec{k}\in 2\pi \mathbb {Z}^2}\biggl [\Big ( \sum \limits _{\begin{array}{c} \varvec{j}\in 2\pi \mathbb {Z}^2 \\ \varvec{j}\ne 0, -\varvec{k} \end{array} } |\varvec{j}|^{2r+1}e^{2\tau |\varvec{j}|} |\hat{f}_{\varvec{j}}(z)|^2\Big )^{\frac{1}{2}} \Big ( \sum \limits _{\begin{array}{c} \varvec{j}\in 2\pi \mathbb {Z}^2 \\ \varvec{j}\ne 0, -\varvec{k} \end{array} } |\varvec{j}+\varvec{k}|^{2r}e^{2\tau |\varvec{j}+\varvec{k}|} |\hat{h}_{-\varvec{j}-\varvec{k}}(z)|^2\Big )^{\frac{1}{2}} \biggr ]\\&\le C_r \Vert A^{r+\frac{1}{2}} e^{\tau A} f(z)\Vert _{L^2(\mathbb {T}^2)} \Vert A^{r+\frac{1}{2}} e^{\tau A} g(z)\Vert _{L^2(\mathbb {T}^2)} \Vert A^{r} e^{\tau A} h(z)\Vert _{L^2(\mathbb {T}^2)}. \end{aligned} \end{aligned}$$

Similarly, we have

$$\begin{aligned} \begin{aligned} A_2&= \sum \limits _{\varvec{j}+\varvec{k}+\varvec{l}=0} C_r |\varvec{k}|^{r+\frac{1}{2}}|\varvec{j}|^{\frac{1}{2}} |\varvec{l}|^{r} e^{\tau |\varvec{j}|}e^{\tau |\varvec{k}|} e^{\tau |\varvec{l}|} |\hat{f}_{\varvec{j}}(z)| |\hat{g}_{\varvec{k}}(z)| |\hat{h}_{\varvec{l}}(z)| \\&\le C_r \Vert A^{r+\frac{1}{2}} e^{\tau A} f(z)\Vert _{L^2(\mathbb {T}^2)} \Vert A^{r+\frac{1}{2}} e^{\tau A} g(z)\Vert _{L^2(\mathbb {T}^2)} \Vert A^{r} e^{\tau A} h(z)\Vert _{L^2(\mathbb {T}^2)}, \end{aligned} \end{aligned}$$

and

$$\begin{aligned} \begin{aligned} A_3&= \sum \limits _{\varvec{j}+\varvec{k}+\varvec{l}=0} C_r |\varvec{k}|^{\frac{1}{2}}|\varvec{j}|^{r} |\varvec{l}|^{r+\frac{1}{2}} e^{\tau |\varvec{j}|}e^{\tau |\varvec{k}|} e^{\tau |\varvec{l}|} |\hat{f}_{\varvec{j}}(z)| |\hat{g}_{\varvec{k}}(z)| |\hat{h}_{\varvec{l}}(z)| \\&\le C_r \Vert A^{r} e^{\tau A} f(z)\Vert _{L^2(\mathbb {T}^2)} \Vert A^{r+\frac{1}{2}} e^{\tau A} g(z)\Vert _{L^2(\mathbb {T}^2)} \Vert A^{r+\frac{1}{2}} e^{\tau A} h(z)\Vert _{L^2(\mathbb {T}^2)}. \end{aligned} \end{aligned}$$

For $A_4$, thanks to Cauchy-Schwarz inequality, since $r>1$, we have

$$\begin{aligned} \begin{aligned} A_4&= \sum \limits _{\varvec{j}+\varvec{k}+\varvec{l}=0} C_r |\varvec{k}|^{r+\frac{1}{2}}|\varvec{l}|^{r+\frac{1}{2}} e^{\tau |\varvec{j}|}e^{\tau |\varvec{k}|} e^{\tau |\varvec{l}|} |\hat{f}_{\varvec{j}}(z)| |\hat{g}_{\varvec{k}}(z)| |\hat{h}_{\varvec{l}}(z)| \\&= C_r \sum \limits _{\varvec{j}\in 2\pi \mathbb {Z}^2 } \biggl [e^{\tau |\varvec{j}|}|\hat{f}_{\varvec{j}}(z)| \sum \limits _{\begin{array}{c} \varvec{k}\in 2\pi \mathbb {Z}^2 \\ \varvec{k}\ne 0, -\varvec{j} \end{array} } |\varvec{k}|^{r+\frac{1}{2}} |\hat{g}_{\varvec{k}}(z)| e^{\tau |\varvec{k}|} |\varvec{j}+\varvec{k}|^{r+\frac{1}{2}}e^{\tau |\varvec{j}+\varvec{k}|}|\hat{h}_{-\varvec{j}-\varvec{k}}| \biggr ]\\&\le C_r \Big \{ |\hat{f}_0(z)| + \Big ( \sum \limits _{\begin{array}{c} \varvec{j}\in 2\pi \mathbb {Z}^2 \\ \varvec{j}\ne 0 \end{array} } |\varvec{j}|^{-2r}\Big )^{\frac{1}{2}} \Big ( \sum \limits _{\begin{array}{c} \varvec{j}\in 2\pi \mathbb {Z}^2 \\ \varvec{j}\ne 0 \end{array} } |\varvec{j}|^{2r} e^{2\tau |\varvec{j}|} |\hat{f}_{\varvec{j}}(z)|^2\Big )^{\frac{1}{2}} \Big \} \\&\qquad \times \sup \limits _{\varvec{j}\in 2\pi \mathbb {Z}^2} \biggl [\Big ( \sum \limits _{\begin{array}{c} \varvec{k}\in 2\pi \mathbb {Z}^2 \\ \varvec{k}\ne 0, -\varvec{j} \end{array} } |\varvec{k}|^{2r+1}e^{2\tau |\varvec{k}|} |\hat{g}_{\varvec{k}}(z)|^2\Big )^{\frac{1}{2}} \Big ( \sum \limits _{\begin{array}{c} \varvec{k}\in 2\pi \mathbb {Z}^2 \\ \varvec{k}\ne 0, -\varvec{j} \end{array} } |\varvec{j}+\varvec{k}|^{2r+1}e^{2\tau |\varvec{j}+\varvec{k}|} |\hat{h}_{-\varvec{j}-\varvec{k}}|^2\Big )^{\frac{1}{2}} \biggr ]\\&\le C_r (\Vert A^{r} e^{\tau A} f(z)\Vert _{L^2(\mathbb {T}^2)} + |\hat{f}_0(z)|) \Vert A^{r+\frac{1}{2}} e^{\tau A} g(z)\Vert _{L^2(\mathbb {T}^2)} \Vert A^{r+\frac{1}{2}} e^{\tau A} h(z)\Vert _{L^2(\mathbb {T}^2)}. \end{aligned} \end{aligned}$$

Combining the estimates for $A_1$ to $A_4$, we achieve the desired inequality. $\square$

### Lemma A.2

For $f, h\in \mathcal {S}_{r+\frac{1}{2},s,\tau }$ and $g, \partial _z g \in \mathcal {S}_{r,s,\tau }$, where $r > \frac{3}{2}$, $s\ge 0$, and $\tau \ge 0$, one has

$$\begin{aligned} \begin{aligned}&\Big |\Big \langle A^r e^{\tau A} \big ((\int _0^z \nabla \cdot f(\varvec{x},s)ds)\partial _z g\big ), A^r e^{\tau A} h \Big \rangle \Big |\\ \le&C_r\Vert A^{r+\frac{1}{2}} e^{\tau A} f\Vert \Vert \partial _z g\Vert _{r,0,\tau } \Vert A^{r+\frac{1}{2}} e^{\tau A} h\Vert . \end{aligned} \end{aligned}$$

### Proof

First, $\Big |\Big \langle A^r e^{\tau A} \big ((\int _0^z \nabla \cdot f(\varvec{x},s)ds)\partial _z g\big ), A^r e^{\tau A} h \Big \rangle \Big | = \Big |\Big \langle (\int _0^z \nabla \cdot f(\varvec{x},s)ds)\partial _z g, A^r e^{\tau A} H \Big \rangle \Big |$. Owing to the Fourier representation in (A.2) , we have

$$\begin{aligned} \begin{aligned}&\Big |\Big \langle (\int _0^z \nabla \cdot f(\varvec{x},s)ds)\partial _z g, A^r e^{\tau A} H \Big \rangle \Big | = \Big |\Big \langle \int _0^z \sum \limits _{\varvec{j}\in 2\pi \mathbb {Z}^2 } \varvec{j} \cdot \hat{f}_{\varvec{j}}(s) e^{i\varvec{j} \cdot \varvec{x}} ds)\partial _z g, A^r e^{\tau A} H \Big \rangle \Big | \\ \le&\int _0^1 \sum \limits _{\varvec{j}+\varvec{k}+\varvec{l}=0} C_r|\varvec{j}|\Big (\int _0^z|\hat{f}_{\varvec{j}}(s)|ds\Big )|\partial _z \hat{g}_{\varvec{k}}(z)|(|\varvec{j}|^r+|\varvec{k}|^r)e^{\tau |\varvec{j}|}e^{\tau |\varvec{k}|}|\varvec{l}|^r e^{\tau |\varvec{l}|}|\hat{h}_{\varvec{l}}(z)| dz \\ \le&\int _0^1 \sum \limits _{\varvec{j}+\varvec{k}+\varvec{l}=0} C_r \Big (|\varvec{k}|^{\frac{1}{2}}|\varvec{j}|^{r+\frac{1}{2}} |\varvec{l}|^{r} + |\varvec{j}|^{r+\frac{1}{2}} |\varvec{l}|^{r+\frac{1}{2}} + |\varvec{j}||\varvec{k}|^{r} |\varvec{l}|^{r} \Big ) \\&\qquad \qquad \times e^{\tau |\varvec{j}|}e^{\tau |\varvec{k}|} e^{\tau |\varvec{l}|} \Big (\int _0^z|\hat{f}_{\varvec{j}}(s)|ds\Big )|\partial _z \hat{g}_{\varvec{k}}(z)| |\hat{h}_{\varvec{l}}(z)| dz =: B_1 + B_2 + B_3 . \end{aligned} \end{aligned}$$

where we have substituted the following inequalities: for $\varvec{j}+\varvec{k}+ \varvec{l}=0$,

$$\begin{aligned} |\varvec{j}|^{\frac{1}{2}} \le (|\varvec{k}|^{\frac{1}{2}} + |\varvec{l}|^{\frac{1}{2}}), \;\;\; |\varvec{l}|^r \le C_r (|\varvec{j}|^r+|\varvec{k}|^r). \end{aligned}$$

Thanks to the Cauchy-Schwarz inequality, since $r > \frac{3}{2}$, we have

$$\begin{aligned} \begin{aligned} B_1&= \int _0^1 \sum _{\varvec{j}+\varvec{k}+\varvec{l}=0} C_r |\varvec{k}|^{\frac{1}{2}}|\varvec{j}|^{r+\frac{1}{2}} |\varvec{l}|^{r} e^{\tau |\varvec{j}|}e^{\tau |\varvec{k}|} e^{\tau |\varvec{l}|} \Big (\int _0^z|\hat{f}_{\varvec{j}}(s)|ds\Big )|\partial _z \hat{g}_{\varvec{k}}(z)| |\hat{h}_{\varvec{l}}(z)| dz \\&= C_r \int _0^1 \sum _{\begin{array}{c} \varvec{k}\in 2\pi \mathbb {Z}^2 \\ \varvec{k} \ne 0 \end{array}} \biggl [|\varvec{k}|^{\frac{1}{2}} |\partial _z \hat{g}_{\varvec{k}}(z)| e^{\tau |\varvec{k}|} \sum _{\varvec{j}\in 2\pi \mathbb {Z}^2 } |\varvec{j}|^{r+\frac{1}{2}} e^{\tau |\varvec{j}|}\Big (\int _0^z|\hat{f}_{\varvec{j}}(s)|ds\Big ) |\varvec{j}+\varvec{k}|^{r}e^{\tau |\varvec{j}+\varvec{k}|}|\hat{h}_{-\varvec{j}-\varvec{k}}(z)| \biggr ]dz \\&\le C_r \int _0^1 \Big (\sum _{\varvec{k}\ne 0} |\varvec{k}|^{1-2r}\Big )^{\frac{1}{2}} \Big (\sum _{\varvec{k}\ne 0} |\varvec{k}|^{2r} |\partial _z \hat{g}_{\varvec{k}}(z)|^2 e^{2\tau |\varvec{k}|}\Big )^{\frac{1}{2}} \sup _{\varvec{k}\ne 0}\biggl [\Big ( \sum _{\varvec{j}\in 2\pi \mathbb {Z}^2} |\varvec{j}|^{2r+1} e^{2\tau |\varvec{j}|} \Vert \hat{f}_{\varvec{j}}\Vert _{L^2_z}^2\Big )^{\frac{1}{2}} \\&\qquad \qquad \times \Big ( \sum _{\varvec{j}\in 2\pi \mathbb {Z}^2} |\varvec{j}+\varvec{k}|^{2r}e^{2\tau |\varvec{j}+\varvec{k}|}|\hat{h}_{-\varvec{j}-\varvec{k}}(z)|^2\Big )^{\frac{1}{2}} \biggr ]dz \\&\le C_r \Vert A^{r+\frac{1}{2}} e^{\tau A} f\Vert \int _0^1 \Vert A^{r} e^{\tau A} \partial _z g(z)\Vert _{L^2(\mathbb {T}^2)} \Vert A^{r} e^{\tau A} h(z)\Vert _{L^2(\mathbb {T}^2)} dz \\&\le C_r \Vert A^{r+\frac{1}{2}} e^{\tau A} f\Vert \Vert A^{r} e^{\tau A} \partial _z g\Vert \Vert A^{r} e^{\tau A} h\Vert , \end{aligned} \end{aligned}$$

For $B_2$, we have

$$\begin{aligned} \begin{aligned} B_2&= \int _0^1 \sum \limits _{\varvec{j}+\varvec{k}+\varvec{l}=0} C_r |\varvec{j}|^{r+\frac{1}{2}} |\varvec{l}|^{r+\frac{1}{2}} e^{\tau |\varvec{j}|}e^{\tau |\varvec{k}|} e^{\tau |\varvec{l}|} \Big (\int _0^z|\hat{f}_{\varvec{j}}(s)|ds\Big )|\partial _z \hat{g}_{\varvec{k}}(z)| |\hat{h}_{\varvec{l}}(z)| dz \\&= C_r \int _0^1 \sum \limits _{\varvec{k}\in 2\pi \mathbb {Z}^2} \biggl [|\partial _z \hat{g}_{\varvec{k}}(z)| e^{\tau |\varvec{k}|} \sum \limits _{\varvec{j}\in 2\pi \mathbb {Z}^2 } |\varvec{j}|^{r+\frac{1}{2}} e^{\tau |\varvec{j}|}\Big (\int _0^z|\hat{f}_{\varvec{j}}(s)|ds\Big ) |\varvec{j}+\varvec{k}|^{r+\frac{1}{2}}e^{\tau |\varvec{j}+\varvec{k}|}|\hat{h}_{-\varvec{j}-\varvec{k}}(z)| \biggr ]dz \\&\le \int _0^1 C_r \Big \{|\partial _z \hat{g}_0(z)|+\Big (\sum \limits _{\varvec{k}\ne 0} |\varvec{k}|^{-2r}\Big )^{\frac{1}{2}} \Big (\sum \limits _{\varvec{k}\ne 0} |\varvec{k}|^{2r} |\partial _z \hat{g}_{\varvec{k}}(z)|^2 e^{2\tau |\varvec{k}|}\Big )^{\frac{1}{2}} \Big \}\\&\qquad \qquad \times \sup \limits _{\varvec{k}\in 2\pi \mathbb {Z}^2}\biggl [\Big ( \sum \limits _{\varvec{j}\in 2\pi \mathbb {Z}^2} |\varvec{j}|^{2r+1} e^{2\tau |\varvec{j}|} \Vert \hat{f}_{\varvec{j}}\Vert _{L^2_z}^2\Big )^{\frac{1}{2}} \Big ( \sum \limits _{\varvec{j}\in 2\pi \mathbb {Z}^2} |\varvec{j}+\varvec{k}|^{2r+1}e^{2\tau |\varvec{j}+\varvec{k}|}|\hat{h}_{-\varvec{j}-\varvec{k}}(z)|^2\Big )^{\frac{1}{2}} \biggr ]dz \\&\le C_r \Vert A^{r+\frac{1}{2}} e^{\tau A} f\Vert \int _0^1 \Big (|\partial _z \hat{g}_0(z)| + \Vert A^{r} e^{\tau A} \partial _z g(z)\Vert _{L^2(\mathbb {T}^2)}\Big ) \Vert A^{r+\frac{1}{2}} e^{\tau A} h(z)\Vert _{L^2(\mathbb {T}^2)} dz \\&\le C_r \Vert A^{r+\frac{1}{2}} e^{\tau A} f\Vert \Vert \partial _z g\Vert _{r,0,\tau } \Vert A^{r+\frac{1}{2}} e^{\tau A} h\Vert . \end{aligned} \end{aligned}$$

The estimate of $B_3$ is similar to that of $B_1$, and one can obtain that

$$\begin{aligned} B_3 \le C_r \Vert A^{r+\frac{1}{2}} e^{\tau A} f\Vert \Vert A^{r} e^{\tau A} \partial _z g\Vert \Vert A^{r} e^{\tau A} h\Vert . \end{aligned}$$

Combine the estimates of $B_1$, $B_2$, and $B_3$, we obtain the desired result. $\square$

### Lemma A.3

For $f, g, h\in \mathcal {S}_{r+\frac{1}{2},s,\tau }$, where $r>1$, $s\ge 0$, and $\tau \ge 0$, one has

$$\begin{aligned} \begin{aligned}&\Big |\Big \langle A^r e^{\tau A} \big ((\nabla \cdot f)g\big ), A^r e^{\tau A} h \Big \rangle \Big | \\ \le&\int _0^1 C_r\Big [ (\Vert A^r e^{\tau A} g(z)\Vert _{L^2(\mathbb {T}^2)} + |\hat{g}_0(z)| ) \Vert A^{r+\frac{1}{2}} e^{\tau A} f(z)\Vert _{L^2(\mathbb {T}^2)} \Vert A^{r+\frac{1}{2}} e^{\tau A} h(z)\Vert _{L^2(\mathbb {T}^2)} \\&+ \Vert A^{r+\frac{1}{2}} e^{\tau A} f(z)\Vert _{L^2(\mathbb {T}^2)} \Vert A^{r+\frac{1}{2}} e^{\tau A} g(z)\Vert _{L^2(\mathbb {T}^2)} \Vert A^{r} e^{\tau A} h(z)\Vert _{L^2(\mathbb {T}^2)}\Big ] dz. \end{aligned} \end{aligned}$$

The proof of Lemma A.3 is almost the same as Lemma A.1, so we omit it.

We will show lemmas which are essential in the study of effect of rotation. Lemma A.4 to Lemma A.6 are concerning the commutator estimates.

### Lemma A.4

For $$f, g, h\in \mathcal {S}_{r+\frac{1}{2},s,\tau }$$, where $$r > 2 $$, $$s\ge 0$$, and $$\tau \ge 0$$, one has

$$\begin{aligned}&\Big |\Big \langle A^r e^{\tau A} (f\cdot \nabla g), A^r e^{\tau A} h \Big \rangle - \Big \langle f\cdot \nabla A^r e^{\tau A} g, A^r e^{\tau A} h \Big \rangle \Big | \nonumber \\&\le C_r \int _0^1 \Vert A^r f(z)\Vert _{L^2(\mathbb {T}^2)} \Vert A^r g(z)\Vert _{L^2(\mathbb {T}^2)} \Vert A^r h(z)\Vert _{L^2(\mathbb {T}^2)} dz \nonumber \\&+ C_r \tau \int _0^1 \Vert A^{r+\frac{1}{2}} e^{\tau A} f(z)\Vert _{L^2(\mathbb {T}^2)} \Vert A^{r+\frac{1}{2}} e^{\tau A} g(z)\Vert _{L^2(\mathbb {T}^2)} \Vert A^{r+\frac{1}{2}} e^{\tau A} h(z)\Vert _{L^2(\mathbb {T}^2)} dz. \end{aligned}$$

Next, we have

### Lemma A.5

For $$f, g, h\in \mathcal {S}_{r+\frac{1}{2},s,\tau }$$, where $$r > 2 $$, $$s\ge 0$$, and $$\tau \ge 0$$, one has

$$\begin{aligned}&\Big |\Big \langle A^r e^{\tau A} \big ( (\nabla \cdot f)g\big ), A^r e^{\tau A} h \Big \rangle - \Big \langle (\nabla \cdot A^r e^{\tau A} f)g, A^r e^{\tau A} h \Big \rangle \Big | \nonumber \\&\le C_r \int _0^1 \Vert A^r f(z)\Vert _{L^2(\mathbb {T}^2)} \Vert A^r g(z)\Vert _{L^2(\mathbb {T}^2)} \Vert A^r h(z)\Vert _{L^2(\mathbb {T}^2)} dz \nonumber \\&+ C_r \tau \int _0^1 \Vert A^{r+\frac{1}{2}} e^{\tau A} f(z)\Vert _{L^2(\mathbb {T}^2)} \Vert A^{r+\frac{1}{2}} e^{\tau A} g(z)\Vert _{L^2(\mathbb {T}^2)} \Vert A^{r+\frac{1}{2}} e^{\tau A} h(z)\Vert _{L^2(\mathbb {T}^2)} dz. \end{aligned}$$

We start with the proof of Theorem A.4. The proof of Theorem A.5 will be similarly.

### Proof of Lemma A.4

First, notice that $\Big |\Big \langle A^r e^{\tau A} (f\cdot \nabla g), A^r e^{\tau A} h \Big \rangle \Big | = \Big |\Big \langle f\cdot \nabla g, A^r e^{\tau A} H \Big \rangle \Big |$, where $H = A^r e^{\tau A} h$. We use Fourier representation of *f*, *g* and *H*, in which we can write

$$\begin{aligned}&f(\varvec{x},z) = \sum \limits _{\varvec{j}\in 2\pi \mathbb {Z}^2} \hat{f}_{\varvec{j}}(z) e^{i\varvec{j}\cdot \varvec{x}}, \\&g(\varvec{x},z) = \sum \limits _{\varvec{k}\in 2\pi \mathbb {Z}^2} \hat{g}_{\varvec{k}}(z) e^{i\varvec{k}\cdot \varvec{x}}, \\&A^r e^{\tau A} H(\varvec{x},z) = \sum \limits _{\varvec{l}\in 2\pi \mathbb {Z}^2} |\varvec{l}|^r e^{\tau |\varvec{l}|} \hat{H}_{\varvec{l}}(z) e^{i\varvec{l}\cdot \varvec{x}}. \end{aligned}$$

Therefore,

$$\begin{aligned}&I :=\Big |\Big \langle A^r e^{\tau A} (f\cdot \nabla g), A^r e^{\tau A} h \Big \rangle - \Big \langle f\cdot \nabla A^r e^{\tau A} g, A^r e^{\tau A} h \Big \rangle \Big | \nonumber \\&= \Big |\Big \langle (f\cdot \nabla g), A^r e^{\tau A} H \Big \rangle - \Big \langle f\cdot \nabla A^r e^{\tau A} g, H \Big \rangle \Big |\nonumber \\&\le \sum \limits _{\varvec{j}+\varvec{k}+\varvec{l}=0} \int _0^1 |\hat{f}_{\varvec{j}}(z)||\varvec{k}||\hat{g}_{\varvec{k}}(z) ||\hat{H}_{\varvec{l}}(z)| \Big | |\varvec{l}|^r e^{\tau |\varvec{l}|} - |\varvec{k}|^r e^{\tau |\varvec{k}|} \Big | dz. \end{aligned}$$

By virtue of the following observation [40]:

For $r\ge 1$ and $\tau \ge 0$, and for all positive $\xi ,\eta \in \mathbb {R}$, we have

A.3 $$\begin{aligned}&|\xi ^r e^{\tau \xi } - \eta ^r e^{\tau \eta }| \le C_r|\xi - \eta |\Big ( |\xi - \eta |^{r-1} + \eta ^{r-1} + \tau (|\xi - \eta |^{r} + \eta ^r)e^{\tau |\xi -\eta |}e^{\tau \eta } \Big ); \end{aligned}$$

with $\xi = |\varvec{l}|$, $\eta = |\varvec{k}|$, and $|\xi - \eta | \le |\varvec{j}|$, inequality (A.3) implies

$$\begin{aligned} I \le C_r \sum \limits _{\varvec{j}+\varvec{k}+\varvec{l}=0} \int _0^1 |\hat{f}_{\varvec{j}}(z)||\varvec{k}||\hat{g}_{\varvec{k}} (z)||\hat{H}_{\varvec{l}}(z)| |\varvec{j}|\Big ( |\varvec{j}|^{r-1} + |\varvec{k}|^{r-1} + \tau (|\varvec{j}|^{r} + |\varvec{k}|^r)e^{\tau |\varvec{j}|}e^{\tau |\varvec{k}|} \Big ) dz. \end{aligned}$$

By the definition of *H*, and since $e^x \le 1+xe^x$ for any $x\ge 0$, we have

$$\begin{aligned} |\hat{H}_{\varvec{l}}(z)| = |\varvec{l}|^r e^{\tau |\varvec{l}|} |\hat{h}_{\varvec{l}}(z)| \le |\varvec{l}|^r(1+\tau |\varvec{l}|e^{\tau |\varvec{l}|}) |\hat{h}_{\varvec{l}}(z)| \le |\varvec{l}|^r |\hat{h}_{\varvec{l}}(z)| + \tau (|\varvec{j}|+|\varvec{k}|) |\hat{H}_{\varvec{l}}(z)|. \end{aligned}$$

Therefore, one obtains that

$$\begin{aligned}&|\hat{H}_{\varvec{l}}(z)| \Big ( |\varvec{j}|^{r-1} + |\varvec{k}|^{r-1} + \tau (|\varvec{j}|^{r} + |\varvec{k}|^{r})e^{\tau |\varvec{k}|}e^{\tau |\varvec{j}|} \Big )\nonumber \\&\le \Big (|\varvec{l}|^r |\hat{h}_{\varvec{l}}(z)| + \tau (|\varvec{j}|+|\varvec{k}|) |\hat{H}_{\varvec{l}}(z)|\Big ) \Big ( |\varvec{k}|^{r-1} + |\varvec{j}|^{r-1} \Big ) + |\hat{H}_{\varvec{l}}(z)| \Big (\tau (|\varvec{k}|^{r} + |\varvec{j}|^{r})e^{\tau |\varvec{k}|}e^{\tau |\varvec{j}|} \Big )\nonumber \\&\le |\hat{h}_{\varvec{l}}(z)| |\varvec{l}|^r(|\varvec{k}|^{r-1} + |\varvec{j}|^{r-1}) + \tau C_r |\hat{H}_{\varvec{l}}(z)|(|\varvec{k}|^{r} + |\varvec{j}|^{r})e^{\tau |\varvec{k}|}e^{\tau |\varvec{j}|}. \end{aligned}$$

Based on this, one has

$$\begin{aligned}&I \le C_r \sum \limits _{\varvec{j}+\varvec{k}+\varvec{l}=0} \int _0^1 |\hat{f}_{\varvec{j}}(z)||\varvec{k}||\hat{g}_{\varvec{k}}(z)| |\varvec{j}||\hat{h}_{\varvec{l}}(z)| |\varvec{l}|^r(|\varvec{k}|^{r-1} + |\varvec{j}|^{r-1}) dz \nonumber \\&+ \tau C_r \sum \limits _{\varvec{j}+\varvec{k}+\varvec{l}=0} \int _0^1 |\hat{f}_{\varvec{j}}(z)||\varvec{k}||\hat{g}_{\varvec{k}}(z)| |\varvec{j}| |\hat{H}_{\varvec{l}}(z)|(|\varvec{k}|^{r} + |\varvec{j}|^{r})e^{\tau |\varvec{k}|}e^{\tau |\varvec{j}|} dz:= I_{1} + I_{2}. \end{aligned}$$

Here

$$\begin{aligned} I_1 = C_r \sum \limits _{\varvec{j}+\varvec{k}+\varvec{l}=0} \int _0^1 \Big ( |\hat{f}_{\varvec{j}}(z)||\varvec{k}|^r|\hat{g}_{\varvec{k}}(z)| |\varvec{j}||\hat{h}_{\varvec{l}}(z)| |\varvec{l}|^r + |\hat{f}_{\varvec{j}}(z)||\varvec{k}||\hat{g}_{\varvec{k}}(z)| |\varvec{j}|^r|\hat{h}_{\varvec{l}}(z)| |\varvec{l}|^r \Big ) dz := \int _0^1 I_{11} + I_{12} dz. \end{aligned}$$

Thanks to Cauchy-Schwarz inequality, since $r> 2$, we have

$$\begin{aligned}&I_{11} = C_r \sum \limits _{\varvec{j}+\varvec{k}+\varvec{l}=0} |\varvec{j}||\hat{f}_{\varvec{j}}(z)||\varvec{k}|^r|\hat{g}_{\varvec{k}}(z)| |\varvec{l}|^r|\hat{h}_{\varvec{l}}(z)| \nonumber \\&= C_r \sum \limits _{\begin{array}{c} \varvec{j}\in 2\pi \mathbb {Z}^2 \\ \varvec{j}\ne 0 \end{array} } |\varvec{j}| |\hat{f}_{\varvec{j}}(z)| \sum \limits _{\begin{array}{c} \varvec{k}\in 2\pi \mathbb {Z}^2 \\ \varvec{k}\ne 0, -\varvec{j} \end{array} } |\varvec{k}|^{r} |\hat{g}_{\varvec{k}}(z)| |\varvec{j}+\varvec{k}|^{r}|\hat{h}_{-\varvec{j}-\varvec{k}}(z)| \nonumber \\&\le C_r \Big ( \sum \limits _{\begin{array}{c} \varvec{j}\in 2\pi \mathbb {Z}^2 \\ \varvec{j}\ne 0 \end{array} } |\varvec{j}|^{2-2r}\Big )^{\frac{1}{2}} \Big ( \sum \limits _{\begin{array}{c} \varvec{j}\in 2\pi \mathbb {Z}^2 \\ \varvec{j}\ne 0 \end{array} } |\varvec{j}|^{2r} |\hat{f}_{\varvec{j}}(z)|^2\Big )^{\frac{1}{2}} \nonumber \\&\times \sup \limits _{\varvec{j}\in 2\pi \mathbb {Z}^2}\Big ( \sum \limits _{\begin{array}{c} \varvec{k}\in 2\pi \mathbb {Z}^2 \\ \varvec{k}\ne 0, -\varvec{j} \end{array} } |\varvec{k}|^{2r} |\hat{g}_{\varvec{k}}(z)|^2\Big )^{\frac{1}{2}} \Big ( \sum \limits _{\begin{array}{c} \varvec{k}\in 2\pi \mathbb {Z}^2 \\ \varvec{k}\ne 0, -\varvec{j} \end{array} } |\varvec{j}+\varvec{k}|^{2r} |\hat{h}_{-\varvec{j}-\varvec{k}}(z)|^2\Big )^{\frac{1}{2}} \nonumber \\&\le C_r \Vert A^r f(z)\Vert _{L^2(\mathbb {T}^2)} \Vert A^r g(z)\Vert _{L^2(\mathbb {T}^2)} \Vert A^r h(z)\Vert _{L^2(\mathbb {T}^2)}. \end{aligned}$$

Similarly, one gets

$$\begin{aligned} I_{12} \le C_r \Vert A^r f(z)\Vert _{L^2(\mathbb {T}^2)} \Vert A^r g(z)\Vert _{L^2(\mathbb {T}^2)} \Vert A^r h(z)\Vert _{L^2(\mathbb {T}^2)}. \end{aligned}$$

Therefore,

$$\begin{aligned} I_1 \le C_r \int _0^1 \Vert A^r f(z)\Vert _{L^2(\mathbb {T}^2)} \Vert A^r g(z)\Vert _{L^2(\mathbb {T}^2)} \Vert A^r h(z)\Vert _{L^2(\mathbb {T}^2)} dz. \end{aligned}$$

Next, we estimate

$$\begin{aligned} \begin{aligned} I_2 = \tau C_r \sum \limits _{\varvec{j}+\varvec{k}+\varvec{l}=0} \int _0^1&\Big ( |\varvec{j}|^{r+1}e^{\tau |\varvec{j}|}|\hat{f}_{\varvec{j}}(z)||\varvec{k}|e^{\tau |\varvec{k}|}|\hat{g}_{\varvec{k}}(z)| |\hat{H}_{\varvec{l}}(z)| \\&+ |\varvec{j}|e^{\tau |\varvec{j}|}|\hat{f}_{\varvec{j}}(z)||\varvec{k}|^{r+1}e^{\tau |\varvec{k}|}|\hat{g}_{\varvec{k}}(z)| |\hat{H}_{\varvec{l}}(z)| \Big ) dz := \int _0^1 I_{21} + I_{22} dz. \end{aligned} \end{aligned}$$

Thanks to Cauchy-Schwarz inequality, since $r> 2$, by using $|\varvec{j}|^{\frac{1}{2}} \le |\varvec{k}|^{\frac{1}{2}} + |\varvec{l}|^{\frac{1}{2}}$, and $|\varvec{k}|^{\frac{1}{2}} + |\varvec{l}|^{\frac{1}{2}} \le 2 |\varvec{k}|^{\frac{1}{2}} |\varvec{l}|^{\frac{1}{2}}$ when $|\varvec{k}|\ge 1$ and $|\varvec{l}|\ge 1$, we have

$$\begin{aligned}&I_{21} = \tau C_r \sum \limits _{\varvec{j}+\varvec{k}+\varvec{l}=0} |\varvec{j}|^{r+1}e^{\tau |\varvec{j}|}|\hat{f}_{\varvec{j}}(z)||\varvec{k}|e^{\tau |\varvec{k}|}|\hat{g}_{\varvec{k}}(z)| |\hat{H}_{\varvec{l}}(z)| \nonumber \\&\le \tau C_r \sum \limits _{\begin{array}{c} \varvec{j}+\varvec{k}+\varvec{l}=0\\ \varvec{j}, \varvec{k}, \varvec{l} \ne 0 \end{array}} |\varvec{j}|^{r+\frac{1}{2}}e^{\tau |\varvec{j}|}|\hat{f}_{\varvec{j}}(z)||\varvec{k}|^{\frac{3}{2}}e^{\tau |\varvec{k}|}|\hat{g}_{\varvec{k}}(z)| |\varvec{l}|^{r+\frac{1}{2}}e^{\tau |\varvec{l}|}|\hat{h}_{\varvec{l}}(z)| \nonumber \\&\le C_r \tau \sum \limits _{\begin{array}{c} \varvec{k}\in 2\pi \mathbb {Z}^2 \\ \varvec{k}\ne 0 \end{array} } |\varvec{k}|^{\frac{3}{2}} |\hat{g}_{\varvec{k}}(z)| e^{\tau |\varvec{k}|} \sum \limits _{\begin{array}{c} \varvec{j}\in 2\pi \mathbb {Z}^2 \\ \varvec{j}\ne 0, -\varvec{k} \end{array} } |\varvec{j}|^{r+\frac{1}{2}} e^{\tau |\varvec{j}|}|\hat{f}_{\varvec{j}}(z)| |\varvec{j}+\varvec{k}|^{r+\frac{1}{2}}e^{\tau |\varvec{j}+\varvec{k}|}|\hat{h}_{-\varvec{j}-\varvec{k}}(z)| \nonumber \\&\le C_r \tau \Big ( \sum \limits _{\begin{array}{c} \varvec{k}\in 2\pi \mathbb {Z}^2 \\ \varvec{k}\ne 0 \end{array} } |\varvec{k}|^{2-2r}\Big )^{\frac{1}{2}} \Big ( \sum \limits _{\begin{array}{c} \varvec{k}\in 2\pi \mathbb {Z}^2 \\ \varvec{k}\ne 0 \end{array} } |\varvec{k}|^{2r+1} e^{2\tau |\varvec{k}|} |\hat{g}_{\varvec{k}}(z)|^2\Big )^{\frac{1}{2}} \nonumber \\&\times \sup \limits _{\varvec{k}\in 2\pi \mathbb {Z}^2}\Big ( \sum \limits _{\begin{array}{c} \varvec{j}\in 2\pi \mathbb {Z}^2 \\ \varvec{j}\ne 0, -\varvec{k} \end{array} } |\varvec{j}|^{2r+1}e^{2\tau |\varvec{j}|} |\hat{f}_{\varvec{j}}(z)|^2\Big )^{\frac{1}{2}} \Big ( \sum \limits _{\begin{array}{c} \varvec{j}\in 2\pi \mathbb {Z}^2 \\ \varvec{j}\ne 0, -\varvec{k} \end{array} } |\varvec{j}+\varvec{k}|^{2r+1}e^{2\tau |\varvec{j}+\varvec{k}|} |\hat{h}_{-\varvec{j}-\varvec{k}}(z)|^2\Big )^{\frac{1}{2}} \nonumber \\&\le C_r \tau \Vert A^{r+\frac{1}{2}} e^{\tau A} f(z)\Vert _{L^2(\mathbb {T}^2)} \Vert A^{r+\frac{1}{2}} e^{\tau A} g(z)\Vert _{L^2(\mathbb {T}^2)} \Vert A^{r+\frac{1}{2}} e^{\tau A} h(z)\Vert _{L^2(\mathbb {T}^2)}. \end{aligned}$$

Similarly, one gets

$$\begin{aligned} I_{22} \le C_r \tau \Vert A^{r+\frac{1}{2}} e^{\tau A} f(z)\Vert _{L^2(\mathbb {T}^2)} \Vert A^{r+\frac{1}{2}} e^{\tau A} g(z)\Vert _{L^2(\mathbb {T}^2)} \Vert A^{r+\frac{1}{2}} e^{\tau A} h(z)\Vert _{L^2(\mathbb {T}^2)}. \end{aligned}$$

Therefore,

$$\begin{aligned} I_2 \le C_r \tau \int _0^1 \Vert A^{r+\frac{1}{2}} e^{\tau A} f(z)\Vert _{L^2(\mathbb {T}^2)} \Vert A^{r+\frac{1}{2}} e^{\tau A} g(z)\Vert _{L^2(\mathbb {T}^2)} \Vert A^{r+\frac{1}{2}} e^{\tau A} h(z)\Vert _{L^2(\mathbb {T}^2)} dz. \end{aligned}$$

$$\square $$

### Lemma A.6

For $$f, g, \partial _z g, h\in \mathcal {S}_{r+\frac{1}{2},s,\tau }$$, where $$r > 2 $$, $$s\ge 0$$, and $$\tau \ge 0$$, one has

$$\begin{aligned}&\Big |\Big \langle A^r e^{\tau A} \Big ( (\int _0^z \nabla \cdot f(\varvec{x},s)ds) \partial _z g \Big ), A^r e^{\tau A} h \Big \rangle - \Big \langle \partial _z g A^r e^{\tau A} (\int _0^z \nabla \cdot f(\varvec{x},s)ds) , A^r e^{\tau A} h \Big \rangle \Big | \\&\le C_r \Vert A^{r} \partial _z g\Vert \Vert A^{r} f\Vert \Vert A^{r} h\Vert + C_r \tau \Vert A^{r+\frac{1}{2}} e^{\tau A} \partial _z g\Vert \Vert A^{r+\frac{1}{2}} e^{\tau A} f\Vert \Vert A^{r+\frac{1}{2}} e^{\tau A} h\Vert . \end{aligned}$$

### Proof

Observe that Lemma A.6 follows directly from Lemma A.5. Indeed, if one replaces *f* by $\int _0^z f(\varvec{x}, s) ds$ and *g* by $\partial _z g$ in Lemma A.5, by the Hölder inequality, one obtains that

$$\begin{aligned} \begin{aligned}&\Big |\Big \langle A^r e^{\tau A} \Big ( (\int _0^z \nabla \cdot f(\varvec{x},s)ds) \partial _z g \Big ), A^r e^{\tau A} h \Big \rangle - \Big \langle \partial _z g A^r e^{\tau A} (\int _0^z \nabla \cdot f(\varvec{x},s)ds) , A^r e^{\tau A} h \Big \rangle \Big | \\ \le&C_r \int _0^1 \Vert A^r \int _0^z f(\varvec{x}, s) ds\Vert _{L^2(\mathbb {T}^2)} \Vert A^r \partial _z g(z)\Vert _{L^2(\mathbb {T}^2)} \Vert A^r h(z)\Vert _{L^2(\mathbb {T}^2)} dz \\&+ C_r \tau \int _0^1 \Vert A^{r+\frac{1}{2}} e^{\tau A} \int _0^z f(\varvec{x}, s) ds\Vert _{L^2(\mathbb {T}^2)} \Vert A^{r+\frac{1}{2}} e^{\tau A} \partial _z g(z) \Vert _{L^2(\mathbb {T}^2)} \Vert A^{r+\frac{1}{2}} e^{\tau A} h(z)\Vert _{L^2(\mathbb {T}^2)} dz \\ \le&C_r \Vert A^{r} \partial _z g\Vert \Vert A^{r} f\Vert \Vert A^{r} h\Vert + C_r \tau \Vert A^{r+\frac{1}{2}} e^{\tau A} \partial _z g\Vert \Vert A^{r+\frac{1}{2}} e^{\tau A} f\Vert \Vert A^{r+\frac{1}{2}} e^{\tau A} h\Vert . \end{aligned} \end{aligned}$$

$$\square $$
